# A progeria syndrome links DNA hypermethylation to age-related pathology

**DOI:** 10.1038/s41588-026-02633-8

**Published:** 2026-06-12

**Authors:** Dan Sarni, Gráinne Neary, Paula L. Carroll, Chris S. Vink, Caroline V. Billard, Tomoya Isobe, Xiong Weng, Jordan R. Portman, Daniel L. McCartney, Patricia Heyn, Rob J. van ‘t Hof, Linda R. Morrison, Carol-Anne Martin, Colin Stok, Margaret E. Harley, Andrea Leitch, Maarten van den Ancker, Nic Robertson, Laura Kitto, Richard Clark, Michael Rennie, Anna Popravko, Jessica J. McClure, David A. Parry, Giuseppina Camiolo, Tom Leah, Hélène Jakobczyk, Roly Megaw, Lisa McKie, Grant F. Marshall, Nika Balkic, Jeanne Amiel, Tania Barragán Arévalo, Grace Bronken McCarthy, Catherine A. Buchanan, Alexandre Buffet, Alberto Cascón, Benjamin Cogne, Solene Conrad, Anna Maria Cueto-González, Maria Currás-Freixes, Gunnar Douzgos Houge, Chin-To Fong, Jaya K. George-Abraham, Kate Gibson, Lourdes Ibáñez, Nicola Longo, Charlotte Lussey-Lepoutre, Bradley S. Miller, Alejandro Moles-Fernandez, Nishitha R. Pillai, Tatiana Tvrdik, Marie Vincent, Emiy Yokoyama, Catherine M. Abbott, Francisco Jose Sanchez-Luque, Katrin Ottersbach, Cosimo De Bari, Anke J. Roelofs, Rebekah Tillotson, Kamil R. Kranc, Sara J. Brown, Riccardo E. Marioni, Mihaela Crisan, Berthold Göttgens, Neil C. Henderson, Robert K. Semple, Kevin B. Myant, Elaine Dzierzak, Martin A. M. Reijns, Duncan Sproul, Andrew P. Jackson

**Affiliations:** 1 MRC Human Genetics Unit, Institute of Genetics and Cancer, The University of Edinburgh, Edinburgh, UK; 2 Easter Bush Pathology, The Royal (Dick) School of Veterinary Studies and the Roslin Institute, The University of Edinburgh, Edinburgh, UK; 3 Centre for Inflammation Research, Institute for Regeneration and Repair, The University of Edinburgh, Edinburgh, UK; 4 CRUK Scotland Centre, Institute of Genetics and Cancer, The University of Edinburgh, Edinburgh, UK; 5 Cambridge Stem Cell Institute, University of Cambridge, Jeffrey Cheah Biomedical Centre, Cambridge, UK; 6 Institute for Neuroscience and Cardiovascular Research, The University of Edinburgh, Edinburgh, UK; 7 Institute of Genetics and Cancer, The University of Edinburgh, Edinburgh, UK; 8 Vanthof Scientific, Rozgarty, Poland; 9 Edinburgh Clinical Research Facility, The University of Edinburgh, Edinburgh, UK; 10 Rheumatology Research Group, Institute of Genetics and Cancer, The University of Edinburgh, Edinburgh, UK; 11 Centre for Regenerative Medicine, Institute for Regeneration and Repair, The University of Edinburgh, Edinburgh, UK; 12 Princess Alexandra Eye Pavilion, NHS Lothian, Edinburgh, UK; 13 Simons Initiative for the Developing Brain, The University of Edinburgh, Edinburgh, UK; 14 Service de Médecine Génomique des Maladies Rares, Hôpital Necker-Enfants Malades, Assistance Publique des Hôpitaux de Paris (AP-HP), Paris, France; 15 Human Genetics Department, National Institute of Pediatrics, Mexico City, Mexico; 16 Genetic Counselling, M Health Fairview, Minneapolis, MN, USA; 17 Dell Children’s Medical Group, Dell Medical School, Austin, TX, USA; 18 Université Paris Cité, Inserm, PARCC, Équipe Labellisée Ligue contre le Cancer, Paris, France; 19 Assistance Publique-Hôpitaux de Paris (AP-HP) Centre, Hôpital Européen Georges Pompidou, Département de Médecine Génomique des Tumeurs et des Cancers, Fédération de Génétique et de Médecine Génomique, Paris, France; 20 Hereditary Endocrine Cancer Group, Spanish National Cancer Research Centre (CNIO), Centro de Investigación Biomédica en Red de Enfermedades Raras (CIBERER), Madrid, Spain; 21 Nantes Université, CHU de Nantes, CNRS, INSERM, l’institut du thorax, Nantes, France; 22 Nantes Université, CHU de Nantes, Service de Génétique Médicale, Nantes, France; 23 Àrea de Genètica Clínica i Molecular (HUVH), Medicine Genetics Research Group (VHIR), Mòdul de Consultes de Genètica Clínica i Malalties Minoritàries, Hospital Universitari Vall d’Hebron, Barcelona, Spain; 24 Familial Cancer Clinical Unit, Human Cancer Genetics Program, Spanish National Cancer Research Centre (CNIO), Madrid, Spain; 25 Department of Medical Genetics, Haukeland University Hospital, Bergen, Norway; 26 Department of Pediatrics, University of Rochester School of Medicine and Dentistry, Rochester, NY, USA; 27 Genetic Health Service New Zealand, Christchurch Hospital, Christchurch, New Zealand; 28 Department of Endocrinology, Pediatric Research Institute Sant Joan de Déu, University of Barcelona, Barcelona, Spain; 29 Centro de Investigación Biomédica en Red de Diabetes y Enfermedades Metabólicas Asociadas (CIBERDEM), ISCIII, Madrid, Spain; 30 Division of Clinical Genetics, Department of Human Genetics, University of California, Los Angeles, Los Angeles, CA, USA; 31 Sorbonne University, Nuclear Medicine Department, Pitié-Salpêtrière Hospital, Assistance Publique Hôpitaux de Paris, Centre de recherche des Cordeliers, Université Paris-Cité, Sorbonne Université, Inserm, U1138, Equipe Labellisée Ligue contre le Cancer, Paris, France; 32 Pediatric Endocrinology, Department of Pediatrics, University of Minnesota Medical School, Minneapolis, MN, USA; 33 Division of Genetics and Metabolism, Department of Pediatrics, University of Minnesota, Minneapolis, MN, USA; 34 Department of Pathology, University of Utah, Salt Lake City, UT, USA; 35 ARUP Laboratories, Salt Lake City, UT, USA; 36 Institute of Parasitology and Biomedicine ‘Lopez-Neyra’, Spanish National Research Council, Granada, Spain; 37 Institute of Cancer Research, London, UK; 38 Centre for Haemato-Oncology, Barts Cancer Institute, Queen Mary University of London, London, UK

## Abstract

Declining tissue function and regenerative capacity underlie many chronic diseases. Experimentally establishing the mechanistic basis for such tissue aging presents substantial challenges, given decades-long timescales and multifactorial origins. Epigenetic alterations have been proposed to have a key etiological role, but whether they are correlative or causal remains a key unanswered question, as does their contribution to specific age-related pathologies. Here we describe an epigenetically driven accelerated aging syndrome. We demonstrate that DNMT3A gain-of-function mutations in Heyn–Sproul–Jackson syndrome recapitulate age-related gains in DNA methylation (DNAme), cause multilineage stem cell dysfunction, and phenocopy aspects of aging in humans and mice. We also show that region-specific DNA hypermethylation at lineage-specific genes can explain reduced stem cell output and lineage skewing. Hence, starting from a Mendelian disorder, we implicate DNAme-mediated stem cell dysfunction in the etiology of medically important age-related hematological, bone and metabolic pathologies, which might be targetable by future therapies.

Aging and age-related disorders develop over decades and are multifactorial in origin, making them challenging to investigate experimentally. Hallmark processes provide a framework for understanding age-related pathologies^1,2^. ‘Epigenetic alterations’ are one such hallmark process, with alterations in DNA methylation (DNAme; both gains and losses) arising within the genome with advancing years. Altered DNAme may correlate with age without functional significance. However, circumstantial evidence suggests causal links. Life-extending manipulations in mice reduce age-associated DNAme changes^3,4^, as does induced pluripotent stem cell reprogramming^3^. In addition, statistical DNAme clocks accurately predict chronological age across tissues and organisms^5^. Deviations in age predictions predict age-associated morbidity and mortality (biological age)^6^, leading to a presumption that DNAme change is causal. Nevertheless, demonstrating a direct causal relationship remains an important outstanding question^2,7^. Furthermore, how DNAme alterations impact at the cellular level and which age-related phenotypes they are responsible for remain to be determined^8^.

Age-related gains in DNAme (hereafter referred to as ‘DNA hypermethylation’) predominantly occur within Polycomb-marked domains^5,9–11^. These regions are usually maintained in a hypomethylated state, with Polycomb complexes, histone and DNA demethylases^12–14^ counteracting DNA methyltransferases.

DNA hypermethylation also occurs in Heyn–Sproul–Jackson syndrome (HESJAS)^15,16^, in which gain-of-function mutations in DNA methyltransferase 3 α, *DNMT3A* (hereafter denoted DNMT3A^GOF^), cause microcephalic dwarfism. These mutations disrupt PWWP-mediated binding to H3K36me2/H3K36me3-marked chromatin^15,17–20^, resulting in enhanced localization to Polycomb-marked regions mediated by interaction of the DNMT3A N-terminal region with H2AK119ub^21–25^. In patients with HESJAS, aberrant DNA hypermethylation is observed in Polycomb-marked DNA methylation valleys (DMVs) containing conserved lineage-defining genes^20^. DNMT3A^GOF^-induced DNA hypermethylation at DMVs does not occur in pluripotent stem cells and accumulates in a time-dependent manner^15,17^.

Here we report that individuals with HESJAS also display multiple age-associated phenotypes, as does a mouse model of this disorder. DNA hypermethylation accumulates in *Dnmt3a*^W326R/+^ adult stem cells, mirroring hypermethylation of these sites during physiological aging. This is accompanied by decreased multilineage adult stem cell output. Using B cell differentiation as an exemplar, we show that DNAme at the promoter of *Pax5*, encoding a key transcription factor, is associated with reduced cellular output in this lineage to account for lymphopenia, one of the age-related pathologies observed. Together, these findings provide experimental evidence that links hypermethylation to physiological aging.

## Results

### Age-related phenotypes in DNMT3A^GOF^ individuals

**Extended Data Fig. 1:**
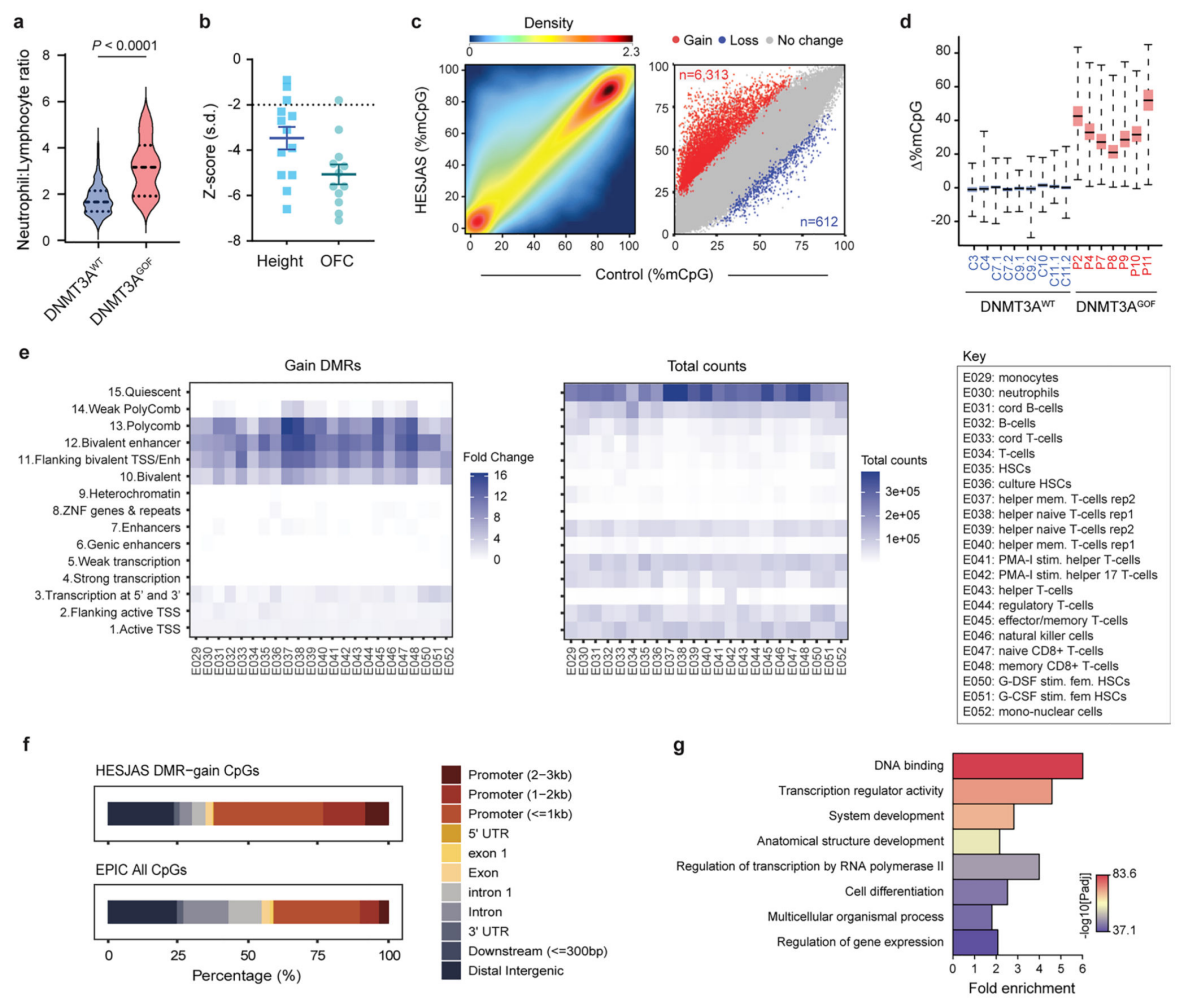
HESJAS DNMT3A^GOF^ mutations cause hypermethylation at Polycomb-marked regions. **a**, Neutrophil:lymphocyte ratio is significantly increased in peripheral blood of HESJAS patients. Controls (DNMT3A^WT^, *n* = 413 from ref. 122), HESJAS (DNMT3A^GOF^, *n* = 10). Middle dashed line, median; upper and lower dotted lines, quartiles Q1 and Q3, respectively. *P* value, two-sided Mann–Whitney U test. **b**, DNMT3A^GOF^ mutations cause growth retardation and microcephaly. Height (*n* = 13) and head circumference (OFC, *n* = 12) are plotted as z-scores (s.d. from population mean adjusted for age and sex). Dashed line at −2 s.d. indicates 95% confidence interval for general population. Mean ± s.e.m. **c**–**e**, EPIC DNA methylation array analysis of peripheral blood leukocytes (PBLs). **c**, Left, kernel density estimate (KDE) plot; right, scatterplot of mean DNA methylation per CpG site (%mCpG) for controls (DNMT3A^WT^) versus HESJAS cases (DNMT3A^GOF^). Significantly differentially methylated CpGs are colored: gain, hypermethylated HESJAS (red, *n* = 6,313); loss, hypomethylated HESJAS (blue, *n* = 612). Nonsignificant, gray. **d**, Mean DNA methylation levels of hypermethylated DMR-associated CpGs depicted in **c**. Box, 25th–75th percentile; whiskers, full data range; centerline, median; Δ%mCpG, percent change of DNA methylation relative to mean of control, DNMT3A^WT^. **e**, Hypermethylated CpGs are enriched for Polycomb-associated ChromHMM states in PBLs. Heatmap, fold enrichment CpGs at hypermethylated HESJAS DMRs relative to all assayed CpGs for that ChromHMM category. Right panel, total count of CpGs for each ChromHMM state. **f**, HESJAS hyper DMRs are significantly enriched for promoter regions. %DMRs intersecting UCSC genome knownGene annotations. **g**, Gene Ontology analysis of hypermethylated DMR-associated genes. Top hits ranked by Benjamini–Hochberg adjusted FDR significance level. DMRs mapped to their closest annotated TSS. Genes associated with DMR probes (*n* = 623 genes) versus genes associated with all array probes (*n* = 20,028), two-sided Fisher’s exact tests.

**Fig. 1:**
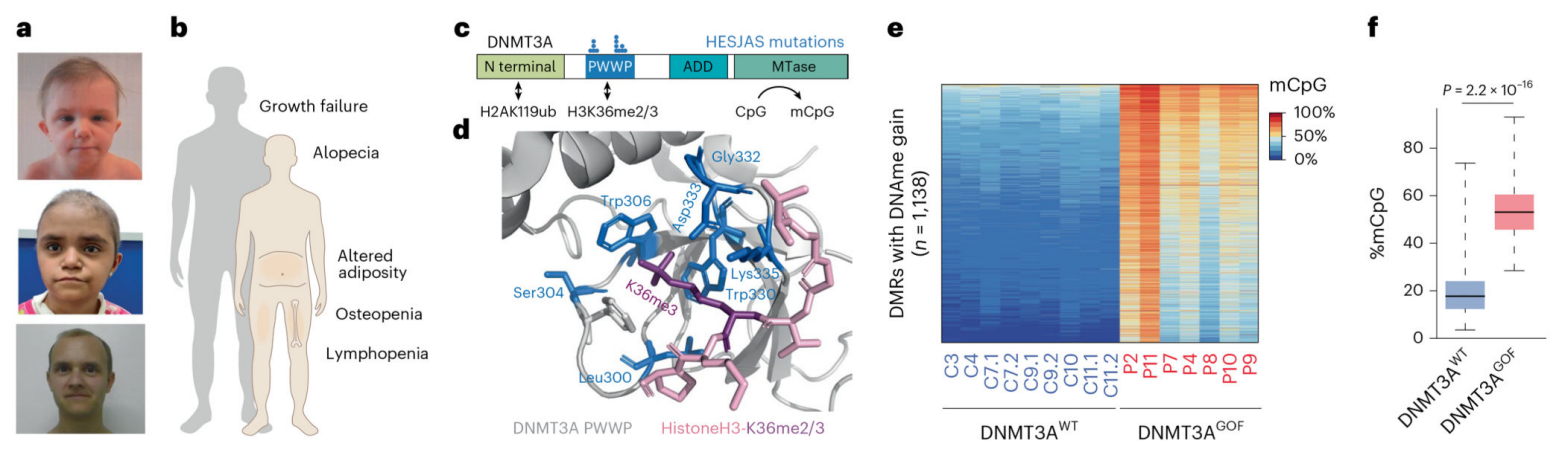
HESJAS syndrome. **a**, Photographs of individuals with HESJAS; aged (top to bottom)—6 years, 11 years, 33 years. Alopecia evident from childhood onwards. **b**, Schematic, age-associated features in HESJAS (Supplementary Table 1). **c**, HESJAS gain-of-function mutations cluster in the PWWP domain. Schematic of DNTM3A domain structure and chromatin interactions. **d**, Structural model of binding pocket of the PWWP domain predicts the HESJAS mutations to disrupt interaction with H3K36me2/H3K36me3. Residues mutated in HESJAS highlighted in blue, backbone of PWWP domain (gray), histone H3 N-terminal tail (pink) and H3K36me3 (purple). **e,f**, HESJAS mutations cause hypermethylation. **e**, Heatmap, hypermethylated DMRs in PB (*n* = 1,138) identified by EPIC DNAme bead array. **f**, Quantification of DNAme in **e**. Plotted mean DNAme per DMR for DNMT3A^WT^ controls (*n* = 9) and DNMT3A^GOF^ HESJAS cases (*n* = 7). Box represents 25th–75th percentile, whiskers represent full data range, centerline represents median and %mCpG represents percent of methylation. *P* value, Wilcoxon rank-sum test, two sided. MTase, methyltransferase domain; C, controls; P, HESJAS cases.

We identified ten individuals (median age = 11 years) with DNMT3A^GOF^ mutations located in the PWWP domain (Fig. 1, Extended Data Fig. 1 and Supplementary Tables 1 and 2). Combined with previously reported cases^15,26,27^, this cohort enabled the redefinition of HESJAS as an accelerated aging syndrome. Notable features include alopecia evident from the first decade (Fig. 1a), severe osteopenia with nontraumatic fractures, lymphopenia and a lipodystrophic appearance (reduced subcutaneous adipose tissue; Supplementary Table 2). Overt lymphopenia was evident in six cases and was associated with an increased propensity for infections. At its most severe, in P11, progressive lymphopenia manifested as a T^−^ B^−^ NK^+^ severe combined immunodeficiency requiring bone marrow (BM) transplantation. Furthermore, the original index case (P1)^15^, re-assessed at a later age, developed BM failure and succumbed to systemic sepsis in her twenties. Across the group, an elevated neutrophil-to-lymphocyte ratio in peripheral blood (PB) counts suggested myeloid-skewed hematopoiesis (Extended Data Fig. 1a). In four cases, loss of subcutaneous fat in the limbs and increased abdominal adiposity were noted, suggestive of lipodystrophy. In P9, this was accompanied by acanthosis nigricans, abnormal lipid profile and liver function tests, consistent with functional impairment of adipose tissue. Asymptomatic paragangliomas, previously associated with *DNMT3A* PWWP mutations^28,29^, were also detected in three older cases. P11 died at the age of 7 years from metastatic osteosarcoma.

All mutations occurred at amino-acid residues directly surrounding the H3K36me2/H3K36me3 binding pocket (Fig. 1c,d). Bead-array DNAme analysis demonstrated the same pattern of hypermethylation at Polycomb-marked regions in all participants for whom PB DNA was available (Fig. 1e,f and Extended Data Fig. 1c–g), confirming that these mutations act by the same gain-of-function mechanism previously reported^15^.

Alopecia, osteopenia, centripetal adipose redistribution (loss of subcutaneous, gain of abdominal fat) and insulin resistance are all age-associated phenotypes in the general population. These are also features of premature aging seen in several monogenic segmental progeroid syndromes, in which subsets of tissues show extreme and accelerated forms of such age-related phenotypes^30,31^. We therefore considered whether HESJAS was also an accelerated aging syndrome, phenocopying a subset of age-related diseases as a ‘segmental progeria’ syndrome. Notably, like HESJAS, other progerias also have restricted growth^30–32^.

**Extended Data Fig. 2:**
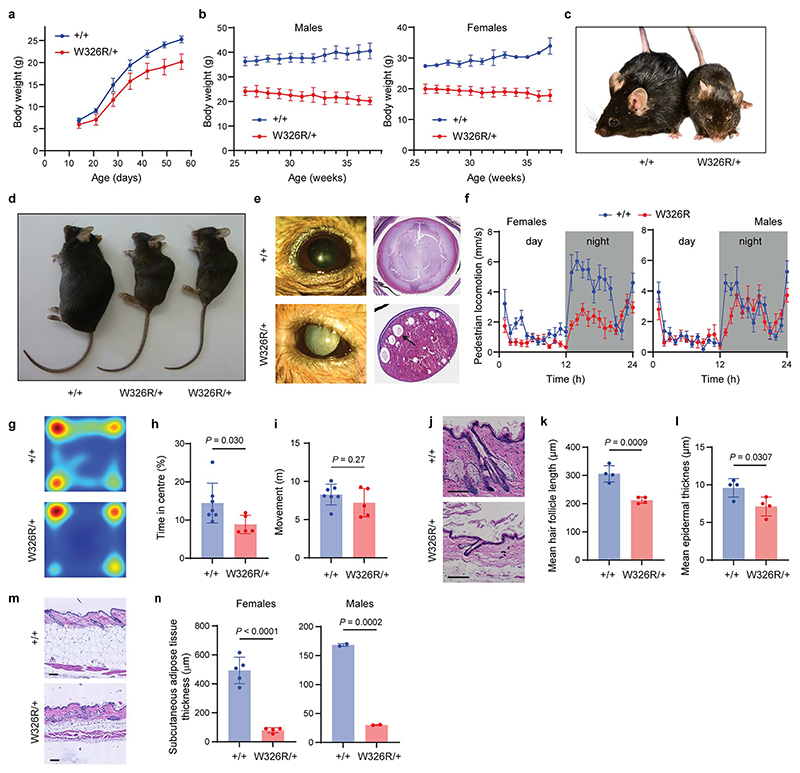
Dnmt3a^W326R/+^ mice model HESJAS syndrome and exhibit premature aging. **a**, Body weight of Dnmt3a^W326R/+^ mice is significantly decreased postweaning. Weight by age, mean ± 95% confidence intervals, *n* = 8 wild-type (+/+), *n* = 11 mutant (W326R/+) male C57BL/6J mice. *P* = 0.0006 by genotype, two-sided 2-way ANOVA. **b**, After 6 months W326R/+ mice of both sexes show a gradual decline in body weight. Males (*n* = 7 +/+, *n* = 6 W326R/+), females (*n* = 3 +/+, *n* = 5 W326R/+). **c**–**e**, Features of premature aging at 1 year of age in the HESJAS mouse model. **c**, 1-year-old W326R/+ mouse next to C57BL/6J wild-type littermate. **d**, Kyphosis. **e**, Senile cataracts with vacuoles and disorganization of fibers in the lens nucleus. **f**–**i**, Neurocognitive deficits. **f**, Reduced nocturnal activity (at 6 months). **g**–**i**, Open field avoidance (positive thigmotaxis). **g**, Density plots (2D heatmaps) of open field positions. **h**, Time in center of field. **i**, Total movement over 20 min (*n* = 7 +/+, *n* = 5 W326R/+ 10–12-month-old male mice). **j**–**l**, Reduced hair follicle length and epidermal thickness in W326R/+ mice. Representative images (**j**) and quantification of mean hair follicle length (**k**) and epidermal thickness (**l**). Data points, individual 6-month-old male mice (*n* = 4 +/+ and *n* = 4 W326R/+). **m,n**, Reduced subcutaneous adipose tissue in old W326R/+ mice. **m**, Representative images. **n**, Quantification (data points, individual 1-year-old females (*n* = 5 +/+, *n* = 4 W326R/+) and 13-month-old males (*n* = 2 +/+, *n* = 2 W326R/+)). For **h, k, l** and **n**, mean ± s.d., unpaired two-sided t-test. For **i**, mean ± s.d., two-sided Mann–Whitney test. For **j** and **m**, scale bars, 100 μm.

**Fig. 2:**
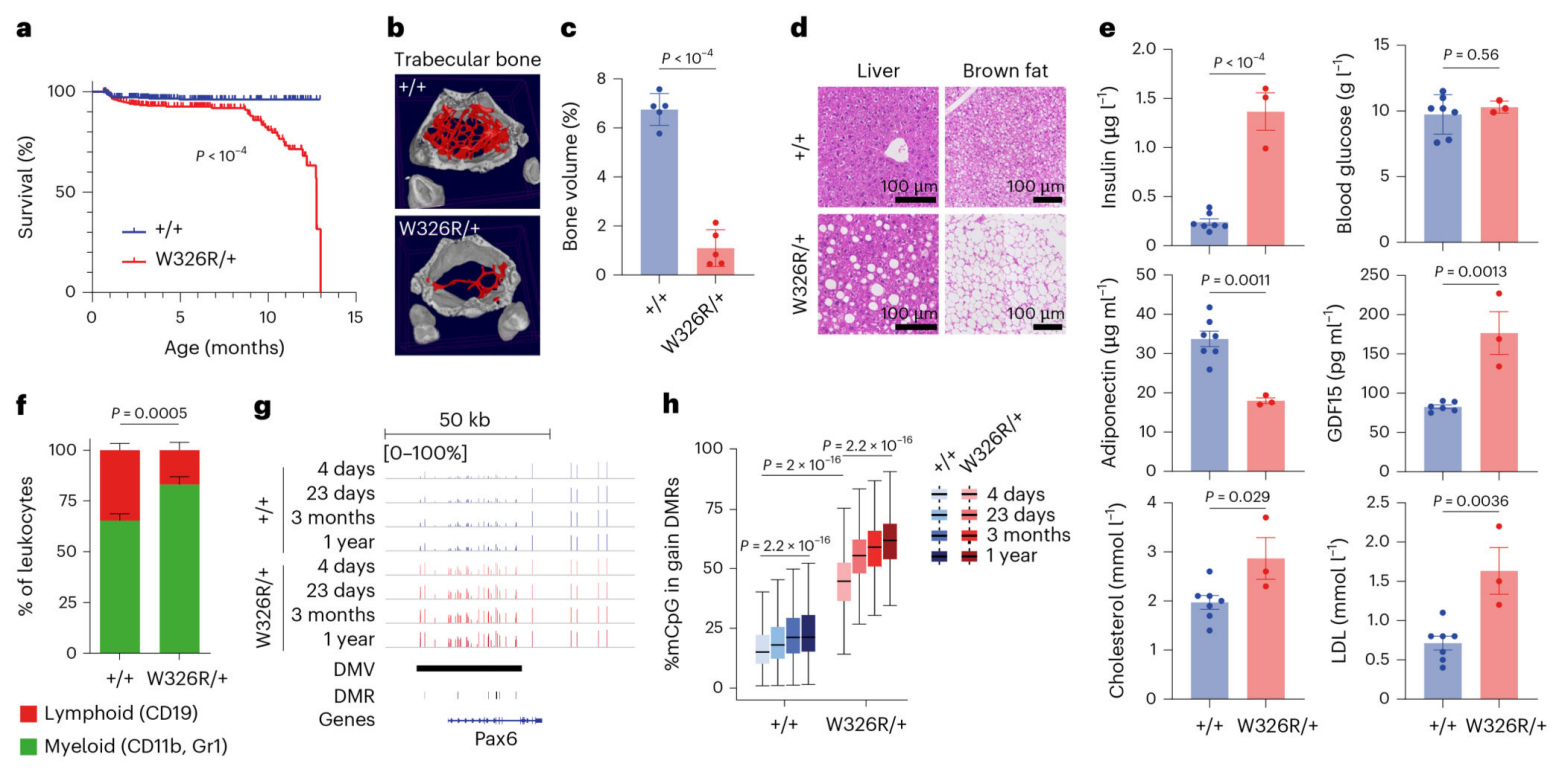
*Dnmt3a*^W326R/+^ mice model HESJAS syndrome and show multiple age-associated phenotypes. **a**, Lifespan of *Dnmt3a*^W326R/+^ mice is significantly decreased; *n* = 380 mutant (W326R/+), *n* = 414 WT (+/+) mice. Log-rank Mantel–Cox test. **b,c**, Severe osteoporosis with marked loss of trabecular bone. **b**, Representative micro-CT cross-section of femoral shaft. Cortical bone in white and trabecular bone in red. **c**, Quantification of trabecular bone volume as percentage of tissue volume; *n* = 5 +/+, *n* = 5 W326R/+ 6-month-old female mice. **d,e**, Lipodystrophic phenotypes are evident at 6 months of age in W326R/+ mice. **d**, Representative images, hepatic steatosis and ‘whitening’ of brown fat. Scale bars, 100 μm. **e**, Hyperinsulinemia, accompanied by elevated GDF15, LDL and cholesterol in W326R/+ mice. For **c** and **e** data points, individual 6-month-old females. Mean ± s.d., unpaired two-sided *t* tests. Male mice similarly affected (Extended Data Fig. 3). **f**, W326R/+ BM populations are myeloid skewed; *n* = 6 mice at 6 months; % myeloid +/+ versus W326R/+, unpaired two-sided *t* test. **g,h**, DNA hypermethylation in W326R/+ BM is present before HSC dysfunction (characterized in Fig. 3) assessed by DNAme array. **g**, Representative genome browser view. Increasing DNAme at the *Pax6* DMV with age. Tracks scaled = 0–100%. **h**, Quantification of DNAme levels in hypermethylated DMRs; *n* = 3 mice per age group per genotype. Box represents 25th–75th percentile, whiskers represent full data range; centerline represents median and two-sided Kruskal–Wallis test for +/+ or W326R/+, respectively, by age, two-sided Wilcoxon rank-sum test for 4-day +/+ versus 4-day W326R/+.

We established a *Dnmt3a*^W326R/*+*^ HESJAS mouse model on a C57BL/6J background. The W326R substitution corresponds to the orthologous residue in several human cases (p.W330R/S/L, P1, P2, P7, P13; Supplementary Table 1). *Dnmt3a*^W326R/+^ mice from 6 months showed increasing frailty with significantly reduced survival (Fig. 2a; median life expectancy of 12.8 months, half that of the expected 26–29 months for C57BL/6J animals^33^). Postnatal growth failure (Extended Data Fig. 2a) and gradual weight loss from 6 months of age were observed (Extended Data Fig. 2b). At endpoint, cataracts, reduced coat condition and kyphosis were also evident (Extended Data Fig. 2c–e). Neurobehaviourally, reduced nocturnal activity was seen at 6 months and positive thigmotaxis was noted on open field testing at 1 year (Extended Data Fig. 2f–i). In 6–12-month-old mice, we also observed decreased hair follicle length, reduced epidermal thickness and loss of subcutaneous fat (Extended Data Fig. 2j–n).

**Extended Data Fig. 3:**
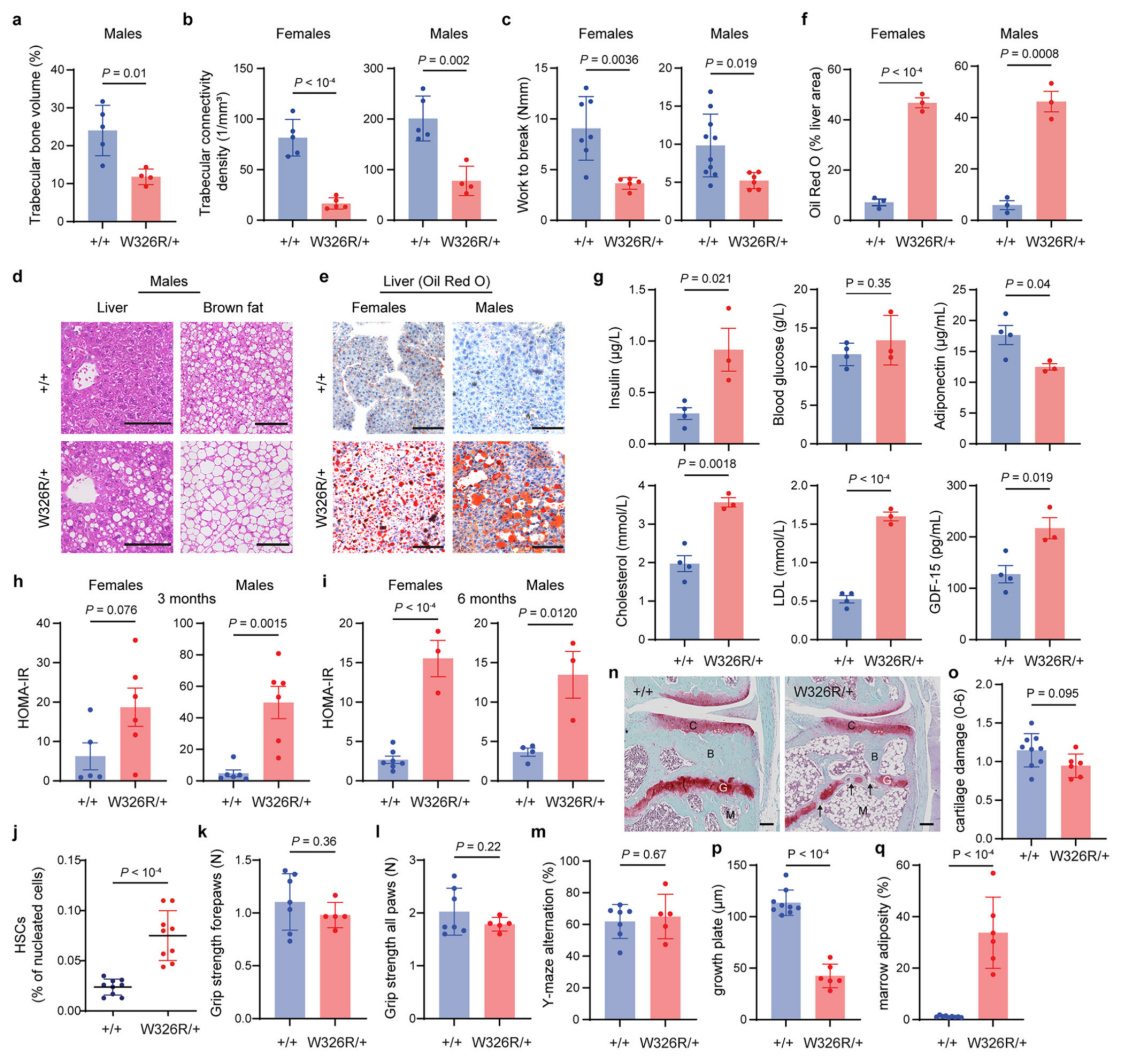
Dnmt3a^W326R/+^ gain-of-function mutation leads to features of segmental progeria in mice. **a**–**c**, Severe osteoporosis and reduced bone strength in 6-month-old W326R/+ mice. **a**, Reduced trabecular bone volume as percentage of tissue volume in male femurs. **b**, Reduced trabecular connectivity density in femurs of both sexes (males, *n* = 5 +/+, *n* = 4 W326R/+; females, *n* = 5 +/+, *n* = 5 W326R/+). **c**, Reduced work to break in mutant mouse femurs of both sexes (females, *n* = 7 +/+, *n* = 5 W326R/+; males, *n* = 10 +/+, *n* = 6 W326R/+). **d**–**i**, Metabolic phenotypes evident at 6 months of age in W326R/+ mice. **d**, Representative images, hepatic steatosis and ‘whitening’ of brown fat in males. **e**, Oil Red O staining shows increased liver fat in male and female W326R/+ mice. **f**, Fatty liver (Oil Red O quantification). **g**, Hyperinsulinemia, accompanied by elevated GDF15 and LDL/cholesterol with normal blood glucose in W326R/+ mice. **h,i**, Insulin resistance in W326R/+ mice. HOMA-IR scores at 3 months (**h**) and 6 months (**i**). For **g** and **i**, data points, individual *n* = 4 +/+ and *n* = 3 W326R/+ male mice; for **h**, *n* = 5 +/+, *n* = 6 W326R/+ females, *n* = 6 +/+, *n* = 6 W326R/+ males; for **i**, *n* = 7 +/+ and *n* = 3 W326R/+ females. **j**, SLAM marker analysis of LSK cells. Increased proportion of HSCs in W326R/+ mice. Data points, percent HSCs of total nucleated cells recovered from two femurs and two tibias from 3-month-old females. *n* = 9 +/+, *n* = 9 W326R/+ mice. **k,l**, No significant change in grip strength. **m**, Y-maze alternations. **n**–**q**, No osteoarthritis, but reduced bone/growth plate and increased bone marrow adiposity in 10–12-month-old W326R/+ mice. **n**, Representative images of the medial tibiofemoral articulation in the knee stained with Safranin O (red, cartilage) and Fast Green (green, bone), showing partially closed growth plate (G, arrows), decreased bone (B) and increased adiposity in marrow (M), while articular cartilage (C) appears normal. **o**, No increased articular cartilage damage characteristic of osteoarthritis, assessed by OARSI scoring (scale 0–6), average score of medial and lateral tibial plateau and femoral condyles. *P* value, Mann–Whitney U test. **p**, Reduced thickness of proximal tibial growth plate. **q**, Increased adiposity, % distal femoral metaphyseal bone marrow space occupied by adipocytes. *P* value, unpaired t-test after log transformation. Data points, individual 10–12-month-old males (*n* = 9 +/+, *n* = 6 W326R/+). For **a**–**c, f**–**m** and **o**–**q**, mean ± s.d., unpaired two-sided t-tests. For **d, e** and **n**, scale bars, 100 μm.

Consistent with accelerated aging, micro-CT on *Dnmt3a*^W326R/+^ femurs identified significant osteoporosis with dramatic loss of trabecular bone and increasing bone fragility in 6-month-old mice (Fig. 2b,c, Extended Data Fig. 3a–c and Supplementary Tables 3–5). In older mutant mice (10–12 months), growth plate thickness was reduced, and BM cellularity decreased alongside increased BM adipos-ity (Extended Data Fig. 3p,q).

Metabolic assessment demonstrated threefold higher plasma insulin in 6-month-old *Dnmt3a*^W326R/*+*^ mice compared to wild-type (WT) littermate controls, despite no difference in blood glucose, indicating marked insulin resistance (Fig. 2e and Extended Data Fig. 3g). This was evident already at 3 months of age as measured by increased HOMA-IR score^34^ (Homeostatic Model Assessment for Insulin Resistance; Extended Data Fig. 3h,i). Reduced serum concentrations of adiponectin, a marker of adipose health, together with increased lipid content of liver (hepatic steatosis), and brown adipose tissue (Fig. 2d,e and Extended Data Fig. 3d–f) suggest functional adipose failure as the likely cause of insulin resistance. These features were observed in mice fed a normal chow diet, which is particularly striking because many mouse models of insulin resistance require a high-fat diet to develop a metabolic phenotype^35^. Metabolic phenotypes appeared to be progressive in both males and females.

Hematological age-associated features were also observed; myeloid skewing was evident in BM at 6 months (Fig. 2f) and was accompanied by an increased percentage of hematopoietic stem cells (HSCs; Extended Data Fig. 3j).

As with other monogenic progeria syndromes, not all features of aging are present in HESJAS. For instance, osteoarthritis, hair graying and reduction in grip strength were not detected nor was performance decreased with Y-maze testing in the *Dnmt3a*^W326R/+^ mouse (Extended Data Figs. 2c,d and 3k–o).

**Extended Data Fig. 4:**
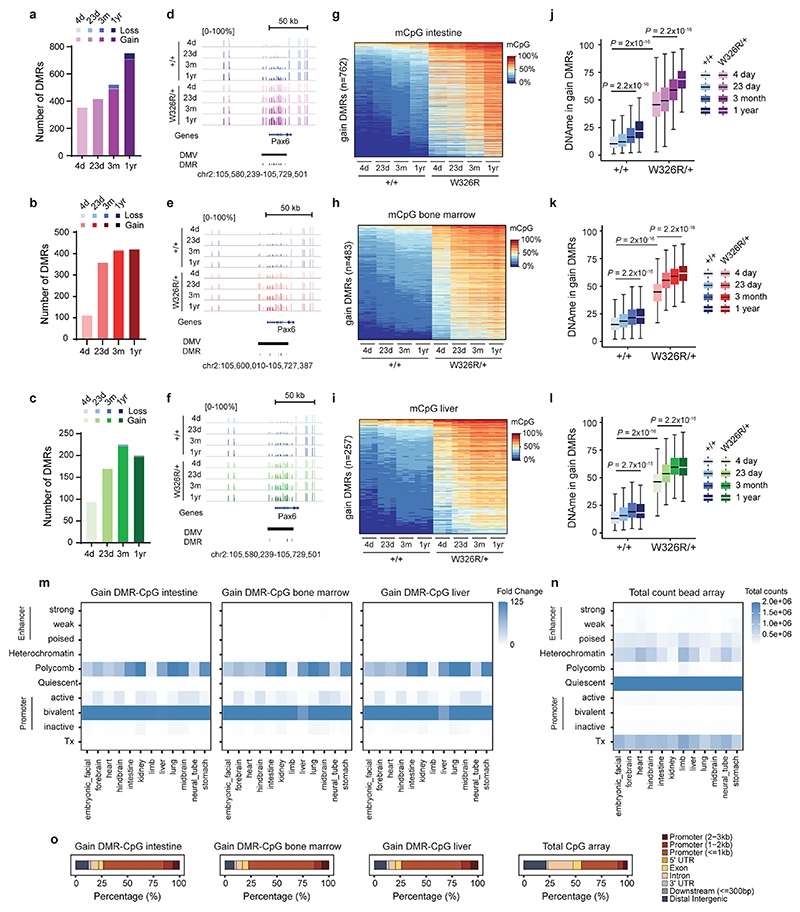
Dnmt3a^W326R/+^ leads to progressive hypermethylation in adult mouse tissues. **a**–**c**, Quantification of differentially methylated regions (DMRs) detected by DNA methylation array shows progressive hypermethylation (gain) in W326R/+. **a**, Intestine. **b**, Bone marrow. **c**, Liver. **d**–**f**, Genome browser view of the hypermethylated *Pax6* locus. All tracks scaled 0–100%. DMV, DNA methylation valley; DMR, differentially methylated region. **d**, Intestine. **e**, Bone marrow. **f**, Liver. **g**–**i**, Heatmap of DMRs hypermethylated in W326R/+. **g**, Intestine (*n* = 762). **h**, Bone marrow (*n* = 483). **i**, Liver (*n* = 257). **j**–**l**, Quantification of DNA methylation at DMRs in *n* = 3 animals per age point per genotype depicted in **g**–**i. j**, Intestine. **k**, Bone marrow. **l**, Liver. Box, 25th–75th percentile; whiskers, full data range; centerline, median; mCpG proportion of DNA methylation along DMRs. *P* value, two-sided, paired Wilcoxon rank-sum tests for 4-day-old +/+ and W326R/+; two-sided Kruskal–Wallis rank-sum test for +/+ by age or W326R/+ by age. **m**, Hypermethylated DMRs in Dnmt3a^W326R/+^ tissues are enriched at Polycomb-marked (Polycomb; bivalent promoter) regions. Heatmap of fold change of CpG frequency at chromatin state categories identified by ChromHMM relative to total CpG count for state. **n**, Total CpG counts for ChromHMM features represented in mouse Illumina bead arrays. **o**, Hypermethylated DMR CpGs are enriched at UCSC genome-annotated promoter regions, relative to total CpG count for annotated feature on array.

Methylation BeadChip profiling demonstrated progressive DNA hypermethylation, in advance of the observed phenotypes, from 4 days of age, in BM, intestine and liver (Fig. 2g,h and Extended Data Fig. 4). Consistent with previous findings in postnatal brain and liver^15,17^, DNA hypermethylation was predominantly present in Polycomb-marked domains and at promoters (Extended Data Fig. 4m–o), as measured by differentially methylated region (DMR) enrichment (corrected for CpG numbers per feature representation given DNAme array biases in genomic feature representation). DNA hypermethylation of the same regions was also apparent with increasing age in WT samples, but at lower levels than in *Dnmt3a*^W326R/+^ tissues.

Hence, *Dnmt3a*^W326R/+^ mice recapitulated human HESJAS, manifesting as a segmental progeria syndrome with multiple accelerated age-related pathologies. Next, we investigated the cellular basis for these phenotypes.

### Lineage-committed blood cells are depleted in *Dnmt3a*^W326R/+^ BM

We had previously proposed that *DNMT3A*^*GOF*^ mutations accelerate differentiation and reduce stem cell pools to explain the microcephalic dwarfism phenotype^15^. Consistent with this notion, the deletion of *Dnmt3a* in adult stem cells causes the opposite phenotype, with impaired differentiation and enhanced stem cell renewal^36,37^ and immortalization^38^. Given hematological abnormalities in our patient cohort, including lymphopenia and BM hypocellularity, we focused our investigation on HSCs and their progeny. As detailed interrogation of HSCs and their function was not feasible in human participants, we examined the *Dnmt3a*^W326R/+^ mouse model.

**Extended Data Fig. 5:**
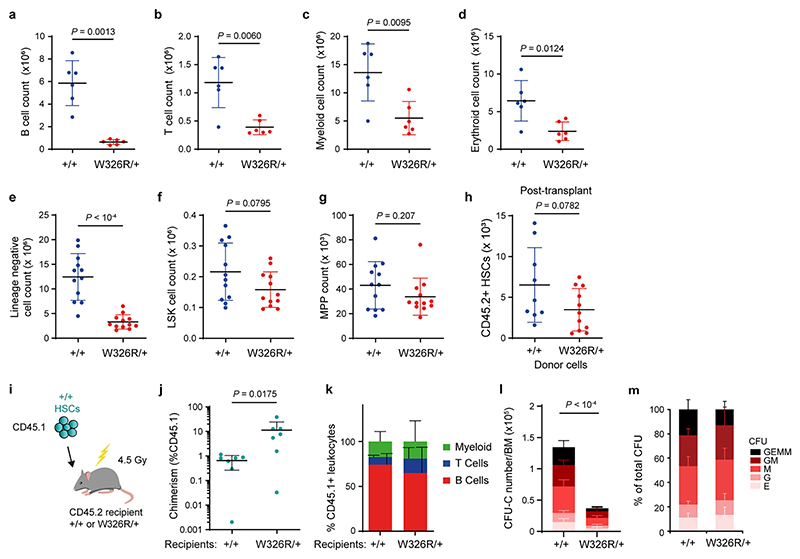
Hematopoietic stem cell function is perturbed in Dnmt3a^W326R/+^ mice. **a**–**g**, Reduced number of mature blood and lineage-negative cells, but not LSK cells or MPPs in W326R/+ mice. **a**–**d**, Quantification of total number of mature B cells (**a**), T cells (**b**), myeloid cells (**c**) and erythroid cells (**d**) in bone marrow from +/+ (*n* = 6) and W326R/+ (*n* = 6) 6-month-old mice. **e**–**g**, Quantification of total number of Lin^−^ cells (**e**), LSK cells (**f**), and MPPs (**g**) in bone marrow from +/+ (*n* = 12) and W326R/+ (*n* = 12) 3–6-month-old mice. **h**, Reduction in transplant W326R/+ HSC numbers is not significant or sufficient to account for depleted levels of circulating CD45.2+ leukocytes from W326R/+ donors (Fig. 3e). Quantification of total CD45.2+ HSCs in bone marrow for +/+ (*n* = 9) and W326R/+ (*n* = 11) donors 4 months post-transplant. For **a**–**h**, cells recovered from two femurs and two tibia per mouse. **i**–**k**, Dnmt3a^W326R/+^ HSC functional deficit is cell autonomous. **i**, Schematic of noncompetitive reverse transplantation experiment. **j**, Wild-type HSCs reconstitute sublethally irradiated W326R/+ mice more efficiently than +/+ mice. Donor chimerism at 4 months in peripheral blood. Data points pooled from 2 experiments, +/+ (*n* = 7) and W326R/+ (*n* = 7) CD45.2 male recipients each receiving 250 HSCs from 2–4-month-old CD45.1 male donors. **k**, Leukocyte populations in peripheral blood are not skewed when wild-type CD45.1 HSCs are transplanted into W326R/+ mice (*n* = 4 +/+; *n* = 6 W326R/+; recipients with <0.7% chimerism were excluded from analysis). **l**, Quantification of colony-forming unit-culture (CFU-C) from 3-month-old bone marrow. +/+ (*n* = 8) and W326R/+ (*n* = 8) mice. E, erythroid; G, granulocyte; M, macrophage; GM, granulocyte–macrophage progenitor; GEMM, common myeloid progenitor. Unpaired t-test for total CFU-C counts. **m**, CFU categories as percentage of total CFU. For **a**–**h**, unpaired two-sided t-test (Welch’s correction for **a** and **b**); for **j**, two-sided Mann–Whitney test, for **l**, two-sided 2-way ANOVA.For **a**–**h** and **j**–**l**, mean ± s.d.

**Fig. 3:**
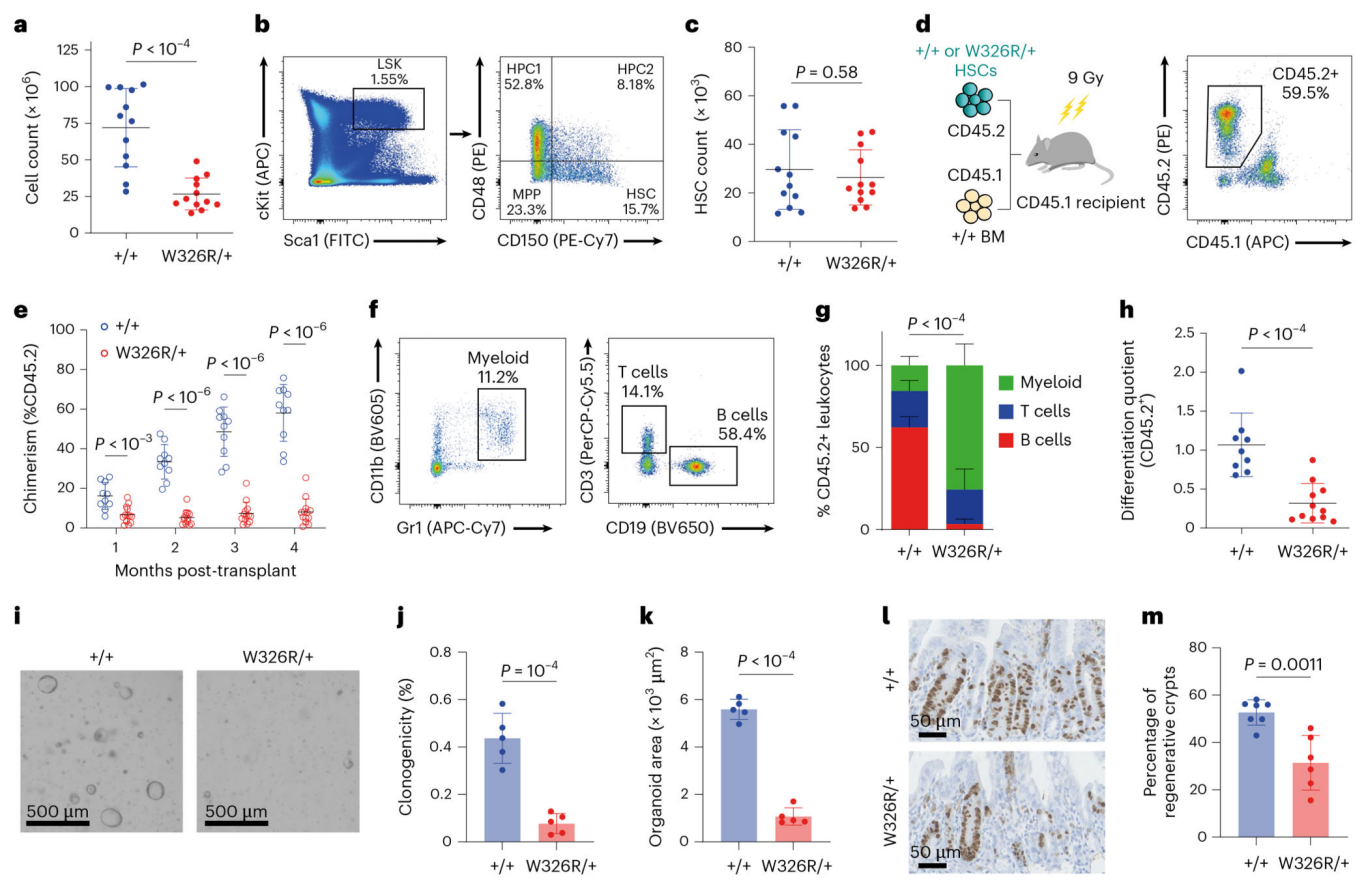
Multilineage functional impairment of adult stem cells in *Dnmt3a*^W326R/+^ mice. **a**–**c**, Total cellularity is reduced in W326R/+ BM, while HSC cell numbers are unchanged. **a**, BM cell number. Data points, total nucleated cells recovered from two femurs and two tibias from *n* = 12 Dnmt3a WT (+/+) and 12 mutant (W326R/+) mice aged 3–6 months. **b**, SLAM marker analysis of LSK cells. Representative FACS plots for WT. HSCs are defined as Lin^−^ Sca^+^ Kit^+^ CD150^+^ CD48^−^. Representative gating strategy is shown in Supplementary Fig. 1. **c**, Total HSC number is similar in +/+ and W326R/+ mice. Data points, as for **a. d**–**h**, Transplanted W326R/+ HSCs are functionally compromised with myeloid skewed and overall reduced output. **d**, Schematic of competitive transplantation of 250 CD45.2 LSK-SLAM HSCs (+/+ or W326R/+ 2–3-month-old donors) into irradiated (9 Gy) CD45.1 recipients (2–4 months old), along with 250,000 CD45.1 BM-derived support cells. Right: representative FACS plot depicting gating of donor-derived WT CD45.2 PB lymphocytes to determine chimerism. **e**, W326R/+ HSCs fail to reconstitute hematopoiesis in competitive BM transplantation. Time course, PB chimerism post-transplant. Data points, *n* = 10 and *n* = 13 recipients of +/+ and W326R/+ HSC donor cells, pooled from four experiments; four male donors per genotype. **f,g**, Hematopoietic progeny of transplanted W326R/+ HSCs show marked myeloid bias. **f**, Representative flow cytometry plots for WT donor, gating of PB cell types at experiment endpoint. Representative gating strategy is shown in Supplementary Fig. 2. **g**, Quantification, donor-derived PBLs at 4 months post-transplant (*n* as for **e**; mean ± s.d., unpaired two-sided *t* test for % myeloid). **h**, Reduced output by donor-derived W326R/+ CD45.2^+^ HSCs in recipient animals is the predominant cause of lower levels of circulating CD45.2^+^ leukocytes. Differentiation quotient, %CD45.2 PB chimerism divided by %CD45.2 BM HSCs at 4 months post-transplant. **i**–**k**, W326R/+ small intestinal crypt cells have substantially lower clonogenic capacity and generate organoids of reduced size. **i**, Representative images of 4-day cultures of single-crypt cells isolated from 3-month to 4-month-old mice. Scale bars, 500 μm. **j,k**, Quantification of colony formation (**j**) and colony size (**k**). Data points, *n* = 5 mice per genotype. **l,m**, W326R/+ small intestinal crypts have reduced regenerative potential in vivo. **l**, Representative images, BrdU labeling of regenerating small intestinal crypts in +/+ and W326R/+ mice 72 h after treatment with oxaliplatin. Scale bars, 50 μm. **m**, Quantification, percentage regenerating crypts. Data points from individual mice (*n* = 7 +/+ and *n* = 6 W326R/+, 6 weeks old) were pooled from two independent experiments. For **a, c, e, h, j, k** and **m**, mean ± s.d. and unpaired two-sided *t* tests. PBLs, peripheral blood leukocytes.

In keeping with our previously proposed model, total cellularity of BM was substantially reduced in *Dnmt3a*^W326R/+^ mice (Fig. 3a). Mature blood cells (lymphocytes, myeloid cells and erythrocytes) were most severely depleted (Extended Data Fig. 5a–d), reflecting decreased output of differentiated cells. Likewise, lineage-negative (Lin^−^) populations were reduced (Extended Data Fig. 5e). However, analysis of LSK (Lin^−^ Sca1^+^ Kit^+^) progenitor populations demonstrated no reduction of either long-term HSCs or multipotent progenitor (MPP) populations (Fig. 3b,c and Extended Data Fig. 5f,g). This led us to reject our hypothesis of stem cell depletion due to accelerated differentiation and instead consider functional HSC impairment as the cause of reduced BM hematopoietic output.

### Transplanted *Dnmt3a*^W326R/+^ HSCs fail to reconstitute hematopoiesis

To assess the functional capacity of *Dnmt3a*^W326R/+^ HSCs, competitive BM transplantation was performed in high-dose irradiated adult mice (Fig. 3d). The transplanted *Dnmt3a*^W326R/+^ HSCs showed a profound impairment in generating mature blood cell output (Fig. 3e). Consistent with steady-state BM analysis of *Dnmt3a*^W326R/+^ mice (Fig. 2f), output from transplanted *Dnmt3a*^W326R/+^ HSCs was also myeloid biased compared to WT HSCs (Fig. 3f,g). Notably, this skewing was opposite to that seen with *Dnmt3a*^−^/^−^ HSC transplantation, where lymphoid lineages were found to be favored^36^. *Dnmt3a*^W326R/+^ HSCs had markedly reduced output, as measured by their differentiation quotient, consistent with compromised stem cell output being the predominant cause of failed PB reconstitution on transplantation (Fig. 3h and Extended Data Fig. 5h).

To address dependency on the BM niche, WT HSCs were transplanted into sublethally irradiated WT and mutant mice (Extended Data Fig. 5i). Engraftment was superior in *Dnmt3a*^W326R/+^ compared to *Dnmt3a*^*+/+*^ recipient animals, and there was no significant difference in the myeloid:lymphoid output of transplanted HSCs between WT and mutant recipients (Extended Data Fig. 5j,k). Assessment of hematopoietic stem/progenitor cell (HSPC) output in a nontransplantation context with ex vivo colony formation assays revealed diminished colony formation from primary cultured *Dnmt3a*^W326R/+^ BM cells across progenitor lineages (Extended Data Fig. 5l,m). Therefore, we conclude that reduced BM hematopoietic cell output and lymphoid depletion are due to cell-autonomous dysfunction in *Dnmt3a*^W326R/+^ HSCs.

### *Dnmt3a*^W326R/+^ intestinal stem cells are functionally compromised

We then considered whether other stem cell populations might also be compromised. Intestinal stem cells (ISCs) have been extensively characterized, and functional assays have been well-established. As in the BM, progressive DNA hypermethylation was observed in the intestine of mutant mice postnatally (Extended Data Fig. 4). Therefore, we assessed the ability of *Dnmt3a*^W326R/+^ ISCs to form intestinal organoid spheroids ex vivo within matrigel^39^. These clonogenic assays demonstrated a significantly reduced number and size of organoids generated from *Dnmt3a*^W326R/+^ single-crypt cells (Fig. 3i–k). As an in vivo assay of ISC function, the regenerative capacity of the intestine was evaluated after chemotherapy treatment. At 72 h after oxaliplatin-mediated ablation of replicating progenitor and stem cells, epithelial regeneration was markedly reduced in *Dnmt3a*^W326R/+^ crypts (Fig. 3l,m). Thus, we conclude that *Dnmt3a*^W326R/+^ ISC function is also impaired. Taken together with the observed HSC phenotype (Fig. 3 and Extended Data Fig. 4), these findings are consistent with multilineage stem cell dysfunction in adult *Dnmt3a*^W326R/+^ mice in at least two distinct tissues.

### Hypermethylation of Polycomb-marked regions in HSCs

**Extended Data Fig. 6:**
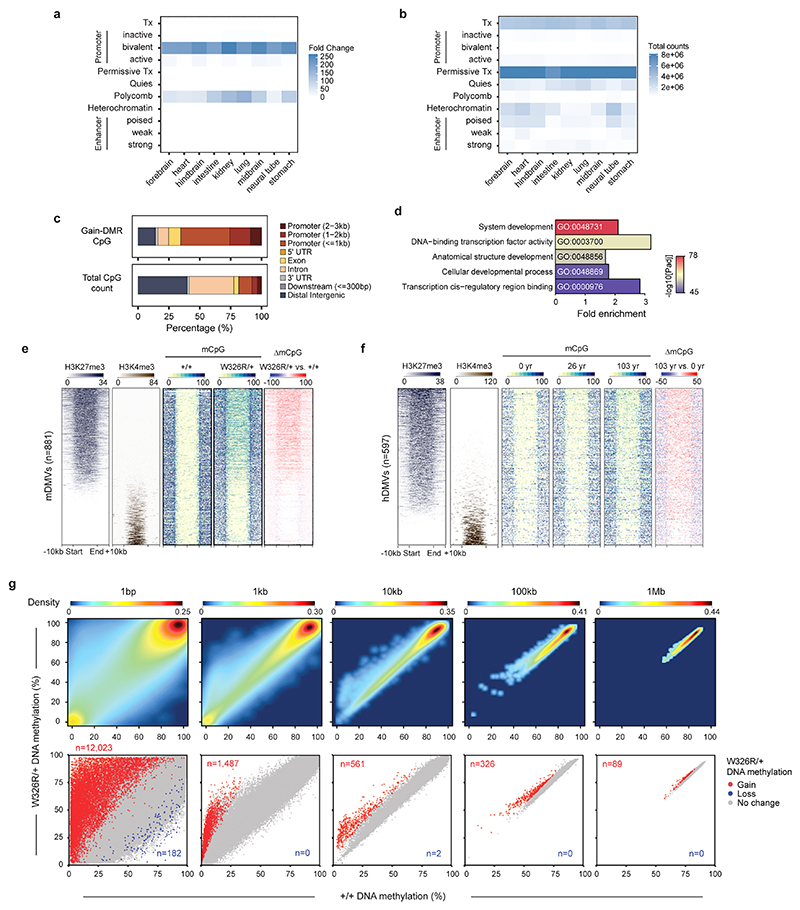
Hypermethylation at Polycomb-associated regions in Dnmt3a^W326R/+^ mouse adult stem cells. **a**–**c**, Hypermethylated DMRs in Dnmt3a^W326R/+^ HSCs detected by EM-seq are enriched at Polycomb-marked (Polycomb; bivalent promoter) regions. **a**, Heatmap of the fold enrichment of CpG frequency at ChromHMM chromatin state categories in HSC hypermethylated DMRs in EM-seq dataset relative to all CpGs for that category. **b**, Heatmap of total CpG site counts for each ChromHMM state in EM-seq dataset. **c**, Hypermethylated DMR CpGs in HSCs are enriched at UCSC genome-annotated promoter regions. **d**, Gene Ontology analysis of HSC hypermethylation DMR associated genes. Highest ranked biological processes and molecular function categories by Benjamini–Hochberg adjusted FDR significance level. Genes associated with DMRs (*n* = 1,715) versus genes associated with all CpGs detected by EM-seq (*n* = 22,860), two-sided Fisher’s exact tests. **e,f**, Dnmt3a^GOF^ mutations in humans and mice lead to hypermethylation in Polycomb-associated DMVs. **e**, Heatmaps of H3K27me3 (GSM8027450), H3K4me3 (GSM8027451), DNA methylation (mCpG) of wild-type (+/+), mutant (W326R/+) HSCs and difference in DNA methylation between mutant and wild-type (ΔmCpG), along mouse DMVs (*n* = 881). **f**, Heatmaps of H3K27me3 (GSM772868), H3K4me3 (GSM997240), DNA methylation (mCpG) of newborn (0 year), 26 years, 103 years leukocytes, and the delta of DNA methylation between 103 years and 0 year (ΔmCpG), along human DMVs (*n* = 597). **g**, Dnmt3a^W326R/+^ gain-of-function mutation in HSCs results almost entirely in DNA hypermethylation assessed by genome-wide EM-seq, with no evidence of hypomethylation of age-associated PMDs. Top: kernel density estimate (KDE) plots of individual CpGs or binned windows. Bottom: scatterplots of the same genomic bins, with significantly differentially methylated CpGs/regions indicated; W326R/+ methylation gain (red); methylation loss (blue); nonsignificant (gray); n, number of significantly differentially methylated regions.

**Fig. 4:**
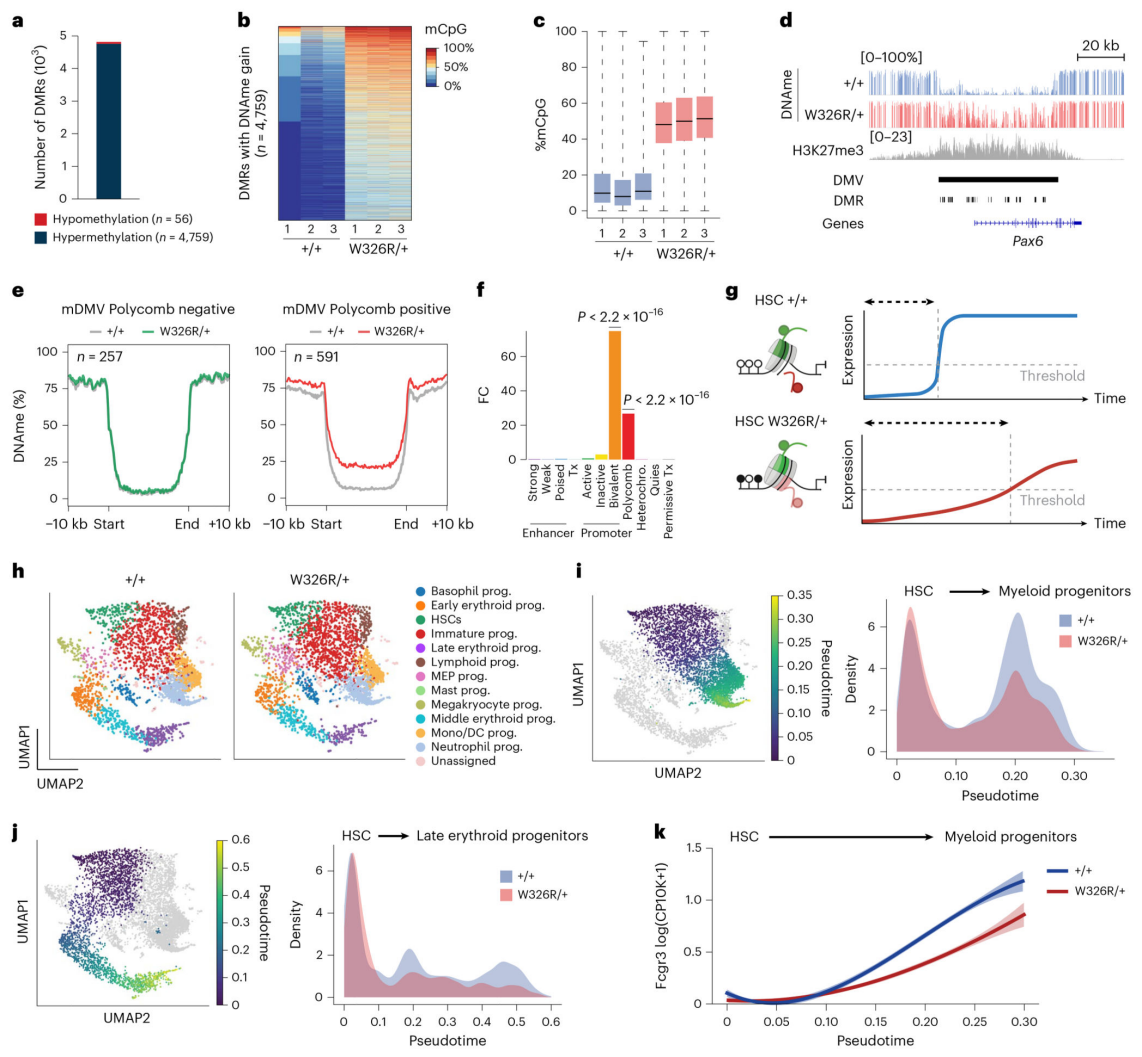
Hypermethylation of *Dnmt3a*^W326R/+^ HSCs impacts multilineage differentiation trajectories. **a**–**c**, DNA hypermethylation detected by EM-seq in *Dnmt3a*^W326R/+^ HSCs. **a**, Number of DMRs identified. **b**, Heatmap, hypermethylated DMRs; *n* = 3 experiments. Columns represent LSK-SLAM HSCs pooled from three mice (3–9-month-old, age matched across genotypes). **c**, Quantification, percent DNAme (%mCpG) for DMRs depicted in **b**. Box represents 25th–75th percentile, whiskers represent full data range and centerline represents median. **d**, Genome browser view, *Pax6* locus, chr2: 105,640,000–105,710,000. Tracks scaled = 0–100%; H3K27me3 track (GSM8027453). **e**, Mean DNAme profile along scaled DMVs with flanking ±10 kb. Left: Polycomb-negative DMVs (*n* = 257). Right: Polycomb-positive DMVs (*n* = 591). **f**, Hypermethylated DMRs in *Dnmt3a*^W326R/+^ HSCs are highly enriched for Polycomb-marked regions and bivalent promoters. HSC hypermethylated DMRs intersected with chromatin state categories defined by ChromHMM for normal mouse fetal liver E16.5 (ref. 40). Fold-change (FC) *P* values, two-sided Fisher’s exact tests, hypermethylated DMR CpGs (*n* = 62,460) versus all CpGs (*n* = 13,627,678). heterochro, heterochromatin; Tx, transcribed. **g**, Model based on ref. 42. **h–k**, scRNA-seq of Lin^−^Kit^+^ BM cells demonstrates delayed pseudotime trajectories in *Dnmt3a*^W326R/+^ progenitors from 2-month-old mice (three pooled per genotype). **h**, UMAP projections for WT and mutant stem cell/progenitor populations onto a reference mouse hematopoietic atlas. W326R/+ plot downsampled to 3,214 data points to provide a direct comparison to WT. prog., progenitors; mono, monocyte; DC, dendritic cell; MEP, megakaryocyte-erythroid progenitors. **i,j**, UMAP pseudotime heatmaps and pseudotime trajectory density graphs for myeloid (**i**) and erythroid differentiation (**j**), respectively. **k**, Pseudotemporal reduction in Fcgr3 expression along the myeloid differentiation trajectory in Dnmt3a mutant mice. Polynomial regression curves individually fitted to mutant (W326R/+) and WT (+/+); 95% confidence intervals; Fcgr3 (CD16), a DMR and myeloid lineage expressed gene. Schematic in **g** created in BioRender; Jackson, A. https://biorender.com/f65p796 (2026).

DNA hypermethylation occurs across adult tissues in HESJAS humans and mice (Figs. 1 and 2 and Extended Data Figs. 1 and 3; refs. 9,10). However, it is not detectable in mouse embryonic stem cells^15^. This raises the question as to whether it is present in adult stem cell populations, which could account for their dysfunction. To address this, we performed enzymatic methyl-seq (EM-seq) on pools of FACS-sorted *Dnmt3a*^*+/+*^ and *Dnmt3a*^W326R/+^ HSCs. This confirmed hypermethylation of Polycomb-marked regions in this adult stem cell population—median DNAme at DMRs was increased to 49.9% in mutants from a baseline in WT of 9.7%. These DMRs were strongly enriched at Polycomb-marked DMVs that contain many lineage-critical genes (Fig. 4a–f). There was 85-fold mean enrichment of hypermethylation at Polycomb sites and 210-fold mean enrichment at bivalent transcription start sites (TSS)/promoters marked by H3K4me3 and H3K27me3 (Extended Data Fig. 6; DMRs intersected with ChromHMM-defined chromatin states^40^). As DMR analysis is based on close proximity of methylated CpGs, widely dispersed hypomethylation in CpG poor regions characteristic of aging^41^ might be overlooked. Therefore, we also analyzed our EM-seq dataset (that identifies CpG methylation sites genome wide at single-nucleotide resolution) independent of DMR calling, at single-CpG-site, 1-kbp, 10-kbp, 100-kbp and 1-Mbp resolution. This identified a very limited number of hypomethylated sites, but notably we did not discover larger-scale regions with reduced methylation that are characteristic of partially methylated domains seen in aging (Extended Data Fig. 6g). We therefore concluded that the *Dnmt3a*^W326R^ mutation causes Polycomb-region-specific hypermethylation in HSCs.

### Transcriptional consequences for hematopoiesis from hypermethylated HSCs

**Extended Data Fig. 7:**
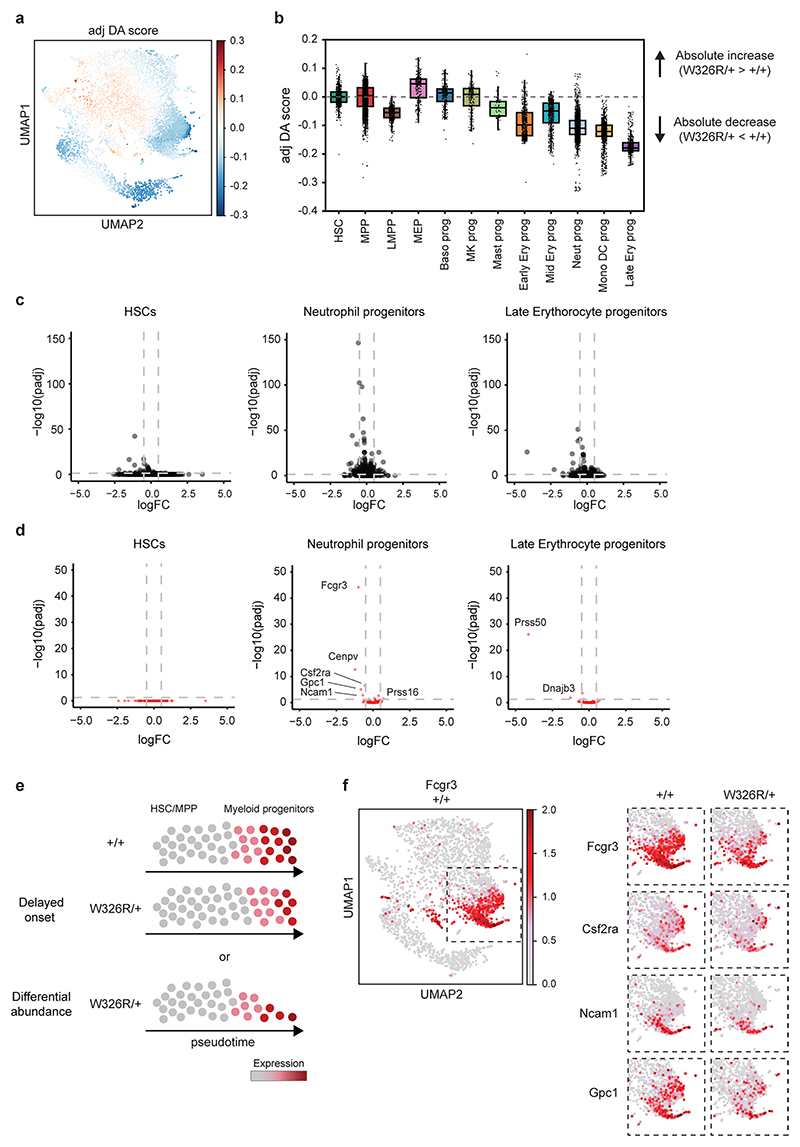
scRNA-seq analysis of Dnmt3a^W326R/+^ HPSCs. **a**, UMAP of differential abundance (DA) of Dnmt3a^W326/R+^ mutant vs wild-type from 2-month-old mice (3 pooled per genotype). W326R/+ plot downsampled to 3,214 data points to provide direct comparison to wild-type. Differential abundance is color coded, higher abundance in mutant, red; lower abundance in mutant, blue. **b**, Quantification of differential abundance presented in **a**. Box, 25th–75th percentile; whiskers, smallest to largest values within 1.5× IQR from the box hinges; centerline, median. HSC, hematopoietic stem cell; prog, progenitors; MPP, multipotent progenitors; LMPP, lympho-myeloid primed progenitors; MEP, megakaryocyte-erythroid progenitors; Baso, Basophil; MK, megakaryocyte; Ery, erythroid; Neut, neutrophil; Mono, monocyte; DC, dendritic cell. **c**, Volcano plots of differentially expressed genes in HSCs, neutrophil progenitors and erythroid progenitors. **d**, Volcano plots of hypermethylated DMR genes in HSCs, neutrophil progenitors and erythroid progenitors. **e**, Model: differentially expressed genes along pseudotime could be due to delayed onset of expression (middle panel) or differential abundance (lower panel). **f**, UMAPs showing differentially expressed genes, Fcgr3, Csf2ra, Ncam1 and Gpc1 within myeloid progenitor populations (dashed box). These demonstrate that decreased expression does occur in individual cells at same reference position in UMAP space, additional to the reduced number of expressing cells.

In contrast to stable transcriptional repression by DNAme, Polycomb repressive marks may be reversed, allowing for the rapid switching on or off of key genes during differentiation in pluripotent stem cells, conceptualized as bivalency^42,43^ and bistability^44^. Given that DNA hypermethylation can disrupt Polycomb regions^15,17,25,45^, we hypothesized that this impairs transcriptional activation of DMR/DMV genes in response to differentiation cues (model; Fig. 4g). To assess these predicted transcriptional consequences, single-cell RNA sequencing (scRNA-seq) was performed on HSPCs (Lin^−^ Kit^+^) from BM of mutant and WT mice. This identified the same stem cell and progenitor populations in both WT and mutant BM, with no *Dnmt3a*^W326R/+^-specific clusters evident (Fig. 4h). However, differential abundance analysis demonstrated significant reductions in later *Dnmt3a*^W326R/+^ progenitor stages, in particular neutrophil and late erythrocyte progenitors (Extended Data Fig. 7a,b), the most differentiated cell types in the Lin^−^ Kit^+^ population analyzed here.

**Extended Data Fig. 8:**
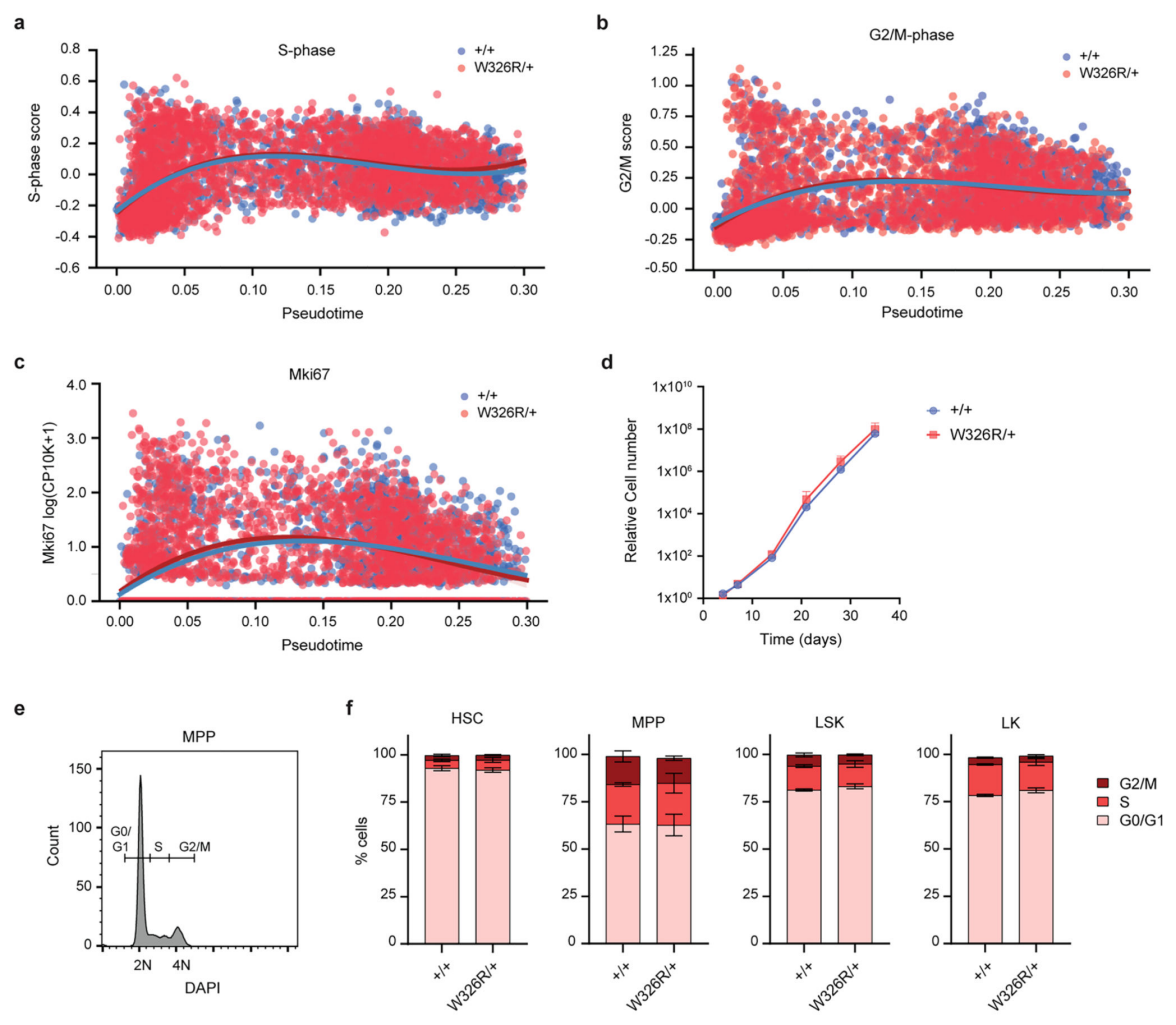
Dnmt3a^W326R/+^ cell cycle dynamics are similar to wild-type HSPCs. **a,b**, scRNA-seq analysis of cell cycle signature genes across the myeloid differentiation trajectory. Scatterplots of transcriptional cell cycle scores for S-phase signature genes across pseudotime (**a**) and G2/M signature genes (**b**). Permutation test for W326R/+ vs +/+: p = 0.54 (>0.05, ns) for **a**; p = 0.76 (>0.05, ns) for **b. c**, Scatterplot of Mki67 gene expression along myeloid differentiation trajectory. Permutation test for W326R/+ vs +/+: p = 0.33 (>0.05, ns). **d**, Growth curves: cell proliferation is not decreased in Dnmt3a^W326R/+^ cultured primary dermal fibroblasts (*n* = 3 mice) compared with isogenic wild-type controls (*n* = 4), geometric mean ± s.d. **e,f**, HSPC FACS analysis of cell cycle. **e**, Representative DNA content histogram of cell populations defined by LSK-SLAM markers. **f**, Quantification of cell cycle profiles in HSC, MPP, LSK and LK populations, as indicated. No significant differences between genotypes were detected by two-sided 2-way ANOVA for HSCs, MPPs, LSK or LK populations; *n* = 3–5-month-old mice per genotype, mean ± s.d.

Gene expression changes were observed for a limited subset of genes in the annotated populations (Extended Data Fig. 7c). Even in the most differentiated cell types analyzed, neutrophil progenitors and erythrocyte progenitors, only 58 and 38 genes, respectively, were differentially expressed (*P*_adj_ < 0.05; and ±0.5 log_2_ fold change (FC)). In contrast, fate trajectory analysis demonstrated markedly reduced frequency of *Dnmt3a*^W326R/+^ cells at later pseudotime points along both myeloid and erythrocyte progenitor differentiation pathways (Fig. 4i,j). Such pseudotime trajectories were therefore consistent with delayed differentiation programs. In addition, several DMR genes encoding lineage-associated maturation markers showed significantly reduced expression in late erythrocyte and neutrophil (myeloid) progenitors (Extended Data Fig. 7d), consistent with a slower rate of transcriptional activation, when plotted against pseudotime and lineage trajectory (Fig. 4k and Extended Data Fig. 7e,f). No changes in cell cycle or cell proliferation, as measured by S-phase, G_2_/M scores and Ki67 expression, were detected in the scRNA-seq analysis, nor were cell cycle changes evident with DNA-content FACS analysis of HSPC subpopulations (Extended Data Fig. 8). Likewise, *Dnmt3a*^W326R/+^ primary dermal fibroblasts grew as well as WT fibroblasts in culture, excluding an intrinsic cell proliferation defect across cell types (Extended Data Fig. 8d). We therefore concluded that impaired transcriptional responses were likely the main reason for reduced cellularity.

### Hypermethylation of *Pax5* impairs activation in early B cell differentiation

**Extended Data Fig. 9:**
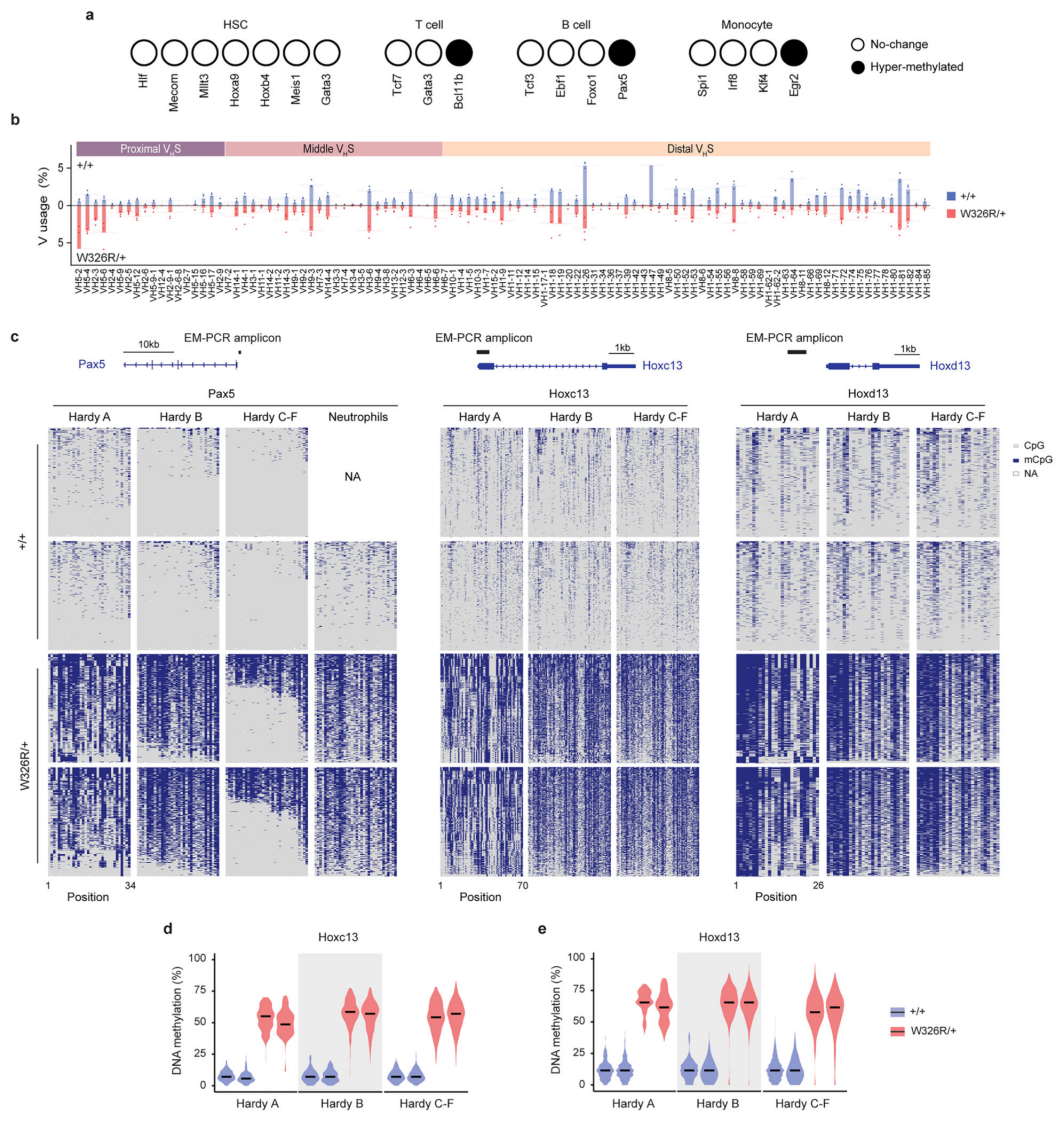
*Pax5* DNA hypermethylation is selectively lost in many B lymphocytes that progress to later differentiation stages. **a**, Hematopoietic lineage-defining transcription factors hypermethylated in the Dnmt3a^W326R/+^ mouse. Filled circles, transcription factors with hyper DMRs identified by EM-Seq of HSCs. **b**, V gene usage repertoire with productive VDJ joins in pro-B cells exhibit a bias towards proximal V_H_ usage in W326R/+. **c**–**e**, EM-PCR of hypermethylation-associated genes in sorted B cell populations (Hardy fractions) from mouse bone marrow. DNA hypermethylation at the *Pax5* promoter in *Dnmt3a*^W326R/*+*^ B lymphocytes is reduced at later stages of B cell differentiation (Hardy C–F), while hypermethylation is not reduced for *Hoxc13* and *Hoxd13* that are not transcriptionally activated in the B-cell lineage. **c**, CpG methylation state matrices of sorted cell populations from individual mice, ages 2–3 months. Blue, mCpG; gray, CpG; white, methylation status not defined. Columns, individual CpGs, numbered from start of amplicon. Rows, individual sequencing reads, *n* = 2 mice per genotype. Reads per sample range 835–4847. Amplicon size: *Pax5* 562 bp, *Hoxc13* 684 bp, *Hoxd13* 666 bp. **d,e**, Quantification from **c**, percentage DNA methylation of individual amplicons for *Hoxc13* (**d**), and *Hoxd13* (**e**). Centerline, median.

**Fig. 5:**
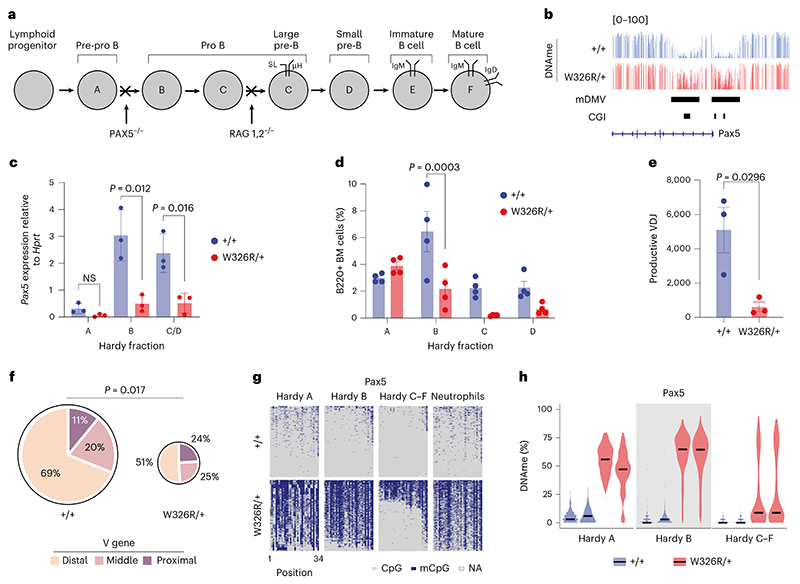
HESJAS B cell differentiation is delayed at the same stage as *Pax5*^−^/^−^ BM. **a**, Schematic of B cell differentiation stages^85^. **b**, DMV hypermethylation at the 5′ end and promoter region of *Pax5* in *Dnmt3a*^W326R/+^ HSCs. Genome browser view, mean DNAme from three sets of HSCs each pooled from three mice (3–9-month-old, aged matched between genotypes); chr4: 44,685,675– 44,729,761; EM-seq. **c**, RT–qPCR analysis demonstrates reduced *Pax5* mRNA expression levels in B cell Hardy fractions B and C/D in 3-month-old *Dnmt3a*^W326R/+^ mice. **d**, B cell numbers are reduced from Hardy B stage onwards. Data points represent 2–3-month-old mice. **e**, Reduced productive VDJ recombination events. Data points represent 2–3-month-old mice. For **c**–**e**, mean ± s.d. and unpaired two-sided *t* tests. **f**, VDJ recombination is biased toward proximal V gene usage, chi-squared test. Percentage VDJ recombination events with proximal, middle and distal V genes. **g,h**, *Pax5* promoter hypermethylation is reduced at later stages of B cell differentiation. Targeted EM-PCR analysis of DNAme of a 562 bp amplicon at the *Pax5* promoter region. **g**, Representative CpG methylation state matrices of sorted cell populations from individual mice, ages 2–3 months. Blue, mCpG; gray, CpG. Columns represent individual CpGs numbered from start of amplicon. Rows represent individual sequencing reads (panels reproduced in EDF9 alongside replicate data at higher magnification). **h**, Violin plot. Percentage DNAme of individual amplicons. Centerline, median *n* = 2. Schematic in **a** is adapted with permission from ref. 86; EMBO Press.

To gain further insight into how BM hypocellularity arose, we examined later differentiation, where the greatest changes in cell number were observed (Extended Data Fig. 5a–e). HESJAS DMRs intersect with lineage-defining genes, with the highest-ranking categories from Gene Ontology (GO) analysis containing many transcription factor genes (Extended Data Fig. 6d and Supplementary Table 6). Most of these transcription factors will not be activated along a specific differentiation trajectory. Indeed, the expectation would be that only one or, at most, a few would be expressed in a given lineage. We identified several candidate genes from the hematopoiesis literature^46–62^, mapping to different myeloid and lymphoid lineages (Extended Data Fig. 9a). As B lymphocyte numbers are the most severely impacted in HESJAS, we then focused our attention on early lymphogenesis. The transcriptional program for B lymphocyte differentiation involves several transcription factors, including Pax5, a master regulator essential for the transition from pre-pro-B cells to pro-B cells^63^ (Hardy A and B, Fig. 5a). The *Pax5* promoter and regulatory regions are hypermethylated in HSCs from *Dnmt3a*^W326R/+^ mice (Fig. 5b). According to dogma, this would be expected to transcriptionally silence the *Pax5* gene. To confirm this prediction, early B cell populations were FACS sorted using established cell-surface markers into Hardy stage fractions (Fig. 5a and Supplementary Fig. 3), which represent sequential steps in early B cell differentiation during which *Pax5* transcription is activated. RT–qPCR demonstrated that, while WT pro-B cells (Hardy B) robustly activated *Pax5* transcription, this was severely compromised in *Dnmt3a*^W326R/+^ pro-B cells (Fig. 5c). In contrast to normal pre-pro-B (Hardy A) cell numbers for W326R/+ mice, there was a marked reduction in pro-B and pre-B cells (Hardy B–D), suggesting that differentiation was largely blocked at this transition (Fig. 5d), consistent with insufficient *Pax5* expression in mutant cells. Furthermore, in line with a key role for Pax5 in promoting distal V gene segment recombination^64,65^, productive VDJ recombination was reduced, with remaining VDJ events strongly biased toward proximal V gene incorporation (Fig. 5e,f). Finally, while DNA hypermethylation was evident in Hardy A and B fractions, this methylation was notably absent from the majority of *Dnmt3a*^W326R/+^ pre-B, immature and mature B cells (Hardy fractions C to F; Fig. 5g,h), whereas hypermethylation remained at *Pax5* in neutrophils and at other loci in B cells (Extended Data Fig. 9c–e). This indicates selective pressure against *Pax5* DNAme for the cells that did progress to later differentiation stages, consistent with cells that have the least abrogated expression of *Pax5* being able to survive the pro-B to pre-B transition. Taken together, we conclude that DNA hypermethylation of a key lineage transcription factor in adult stem cells is associated with impaired differentiation, promoting an age-related phenotype.

### *DNMT3A*^*GOF*^-related DNA hypermethylation corresponds to DNAme gains in human aging

**Extended Data Fig. 10:**
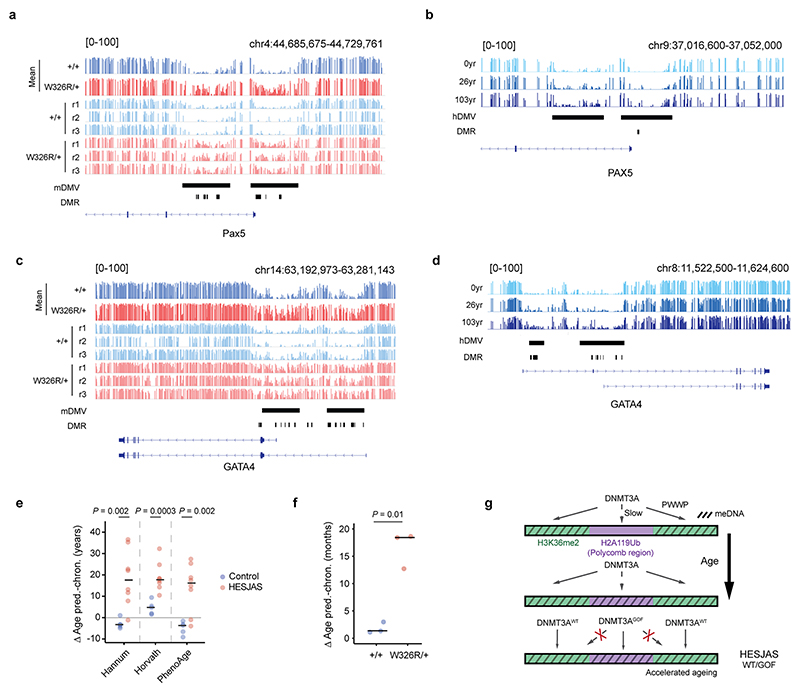
Examples of hypermethylated transcription factor promoter regions; accelerated epigenetic clock aging in HESJAS. **a,b**, Genome browser view of *Pax5* promoter region. **a**, Dnmt3a^W326R/+^ mouse HSCs. Tracks scaled 0–100%. EM-seq of HSCs pooled from 3 mice with experimental replicates, r1-3. Mean, mean DNA methylation of three replicates. mDMV, mouse DNA methylation valleys; DMR, differentially methylated regions. **b**, Human leukocytes, three healthy individuals aged 0 (newborn), 26 and 103 years. hDMVs, human DNA methyation valleys; **c,d**, Genome browser view of the *Gata4* locus in mouse HSCs (**c**) and human lymphocytes (**d**). **e,f**, DNMT3A^GOF^ leads to increased predicted age in HESJAS and mice. **e**, Epi-clock age prediction for HESJAS patients is significantly increased compared to controls. Difference between chronological age and age calculated by indicated epi-clocks plotted for individual cases and controls. Data points, individual samples. **f**, Mouse epi-clock ages for Dnmt3a^W326R/+^ HSCs are predicted to be significantly older compared with age-matched wild-type controls (+/+). Data points, difference from chronological age plotted for HSCs pooled from 3 mice. Black line, median. For **e** and **f**, *P* values, Welch’s two-sided two-sample t-test. **g**, Model: hypermethylation of Polycomb regions by wild-type DNMT3A during physiological aging. Wild-type DNMT3A1 is competitively attracted by two distinct chromatin marks, H3K36me2/3 and H2AK119Ub that are present in mutually exclusive genomic regions^10,21^. The PWWP-H3K36me2/3 interaction normally restrains DNMT3A1 methylation of Polycomb regions containing H2AK119Ub marks. Overexpression of DNMT3A1 can be sufficient to methylate Polycomb regions^123^ and wild-type DNMT3A1 is distinctive from the other de novo DNA methyltransferase, DNMT3B, in having an N-terminal UDR domain mediating this K119Ub interaction. Therefore, low-level wild-type DNMT3A could gradually methylate CpGs at Polycomb sites during physiological aging. In HESJAS, PWWP mutations disrupt the H3K36me2/3 enhancing the attraction of DNMT3A to Polycomb-marked regions, accelerating the rate of DNA methylation, and the onset of associated pathologies. Hence, HESJAS provides an accelerated model for wild-type Dnmt3a’s action at Polycomb regions, by tipping the balance in favor of the UDR–K119Ub interaction.

**Fig. 6:**
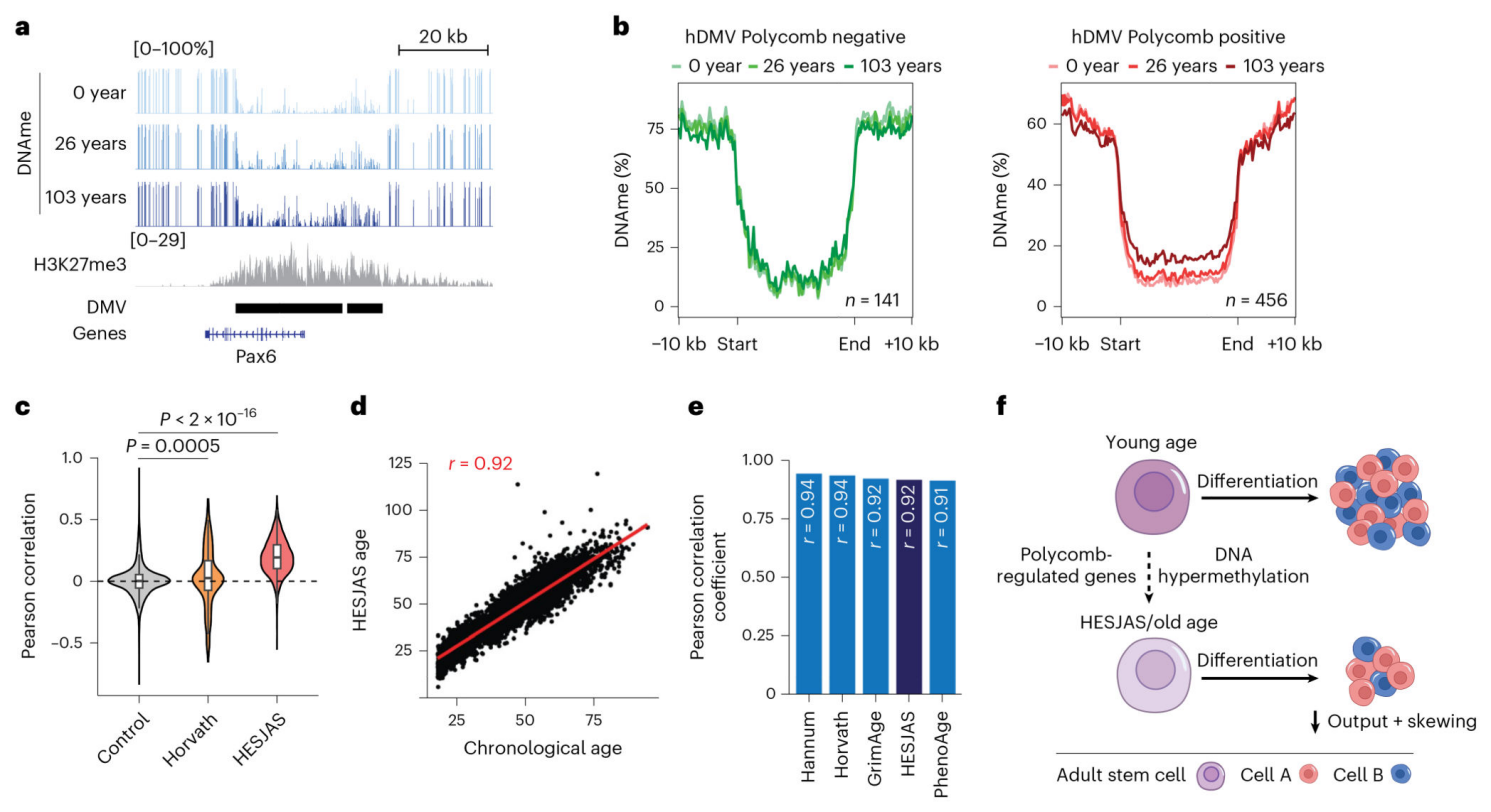
DNMT3A^GOF^ hypermethylation corresponds to sites of DNAme gains in human aging. **a,b**, Polycomb-marked DMVs are hypermethylated in lymphocytes from a 103-year-old individual. **a**, Genome browser view, *PAX6* locus, chr11: 31,795,000–31,875,000. Tracks scaled = 0–100%. DNAme of three healthy individuals aged 0 (newborn), 26 and 103 years. WGBS data reanalyzed from ref. 66. H3K27me3 track = GSM772868; 0-year, 103-year CD4^+^ lymphocytes; 26-year PBLs. **b**, Mean DNAme across scaled DMVs with flanking ±10 kb. Left: Polycomb-negative DMVs (*n* = 141). Right: Polycomb-positive DMVs (*n* = 456). **c**, Pearson correlation DNAme at individual CpG sites versus age in individuals from GS. Control, all CpG probes in Illumina 450K array (*n* = 433,801); Horvath, CpGs used in Horvath clock (*n* = 332); HESJAS, hypermethylated HESJAS CpG sites (*n* = 2,646). *P* values, two-sided Wilcoxon rank-sum test. Centerline represents median, box represents 25th–75th percentile and whiskers represent full data range. **d**, Scatterplot of chronological age versus HESJAS clock predicted age for individuals from GS. **e**, Pearson correlation coefficients for Epi-clocks prediction of age versus chronological age for *n* = 5,085 individuals from the GS population study. **f**, Increased DNAme at evolutionary conserved regions links hallmarks of aging. Model, in HESJAS and aging, DNA hypermethylation at Polycomb-regulated genes in adult stem cells reduces stem cell output, altering the balance of mature cells generated by multilineage stem cells. Schematic in **f** created in BioRender; Jackson, A. https://biorender.com/b15c152 (2026).

Over 90% of sites that gain DNAme with age occur within Polycomb-marked regions^9^, suggesting substantial intersection with HESJAS hypermethylation. Indeed, in mice, at *Dnmt3a*^W326R/+^ hyper-DMRs, we found DNAme to be significantly higher in aged mice (Fig. 2g,h and Extended Data Fig. 4). Likewise, in humans, HESJAS DNA hypermethylation paralleled aging, with reanalysis of a published WGBS dataset derived from neonatal, young and centenarian leukocytes^66^ demonstrating elevated DNAme with age, specifically at Polycomb-marked DMVs (Fig. 6a,b and Extended Data Fig. 6f). Similarly, hypermethylation occurred at the DMVs of lineage-defining genes in mouse *Dnmt3a*^W326R/+^ HSCs (Extended Data Fig. 9a). DNAme levels at individual HESJAS hyper-DMR CpGs correlated positively with age, performing just as well as the CpGs used to derive Horvath’s pan-tissue DNAme clock^67^, when examined in 18,869 individuals from the Generation Scotland (GS) population cohort (Fig. 6c). Also, a DNAme clock derived from HESJAS hyper-DMR CpGs performed comparably to several published clocks^67–70^ in the GS dataset for predicting chronological age (Fig. 6d,e). In addition, existing clocks predicted accelerated age in both patients with HESJAS and the mouse model^67–71^ (Extended Data Fig. 10e,f). Hence, HESJAS and age-related DNA hypermethylation intersect in humans and mice. So, HESJAS and *Dnmt3a*^W326R/+^ mice represent an accelerated and accentuated model for normal age-associated DNA hypermethylation at such sites.

## Discussion

Here we report HESJAS as a new monogenic progeria driven by gain-of-function *DNMT3A* mutations. In apparent contrast, HESJAS was previously identified as a disorder of extreme size reduction (microcephalic dwarfism)^15^. However, reduced size is also seen in other monogenic progerias and mouse models^30,31,72,73^. Furthermore, multilineage reductions in stem cell output could account for both its impact on developmental growth and for reduced regenerative capacity in adult life.

Several medically important pathologies associated with HESJAS also underlie old-age morbidity. Dyslipidemia and insulin resistance predispose to atherosclerosis and type 2 diabetes, respectively^74^. Likewise, attenuated lymphoid lineage output contributes to age-related decline in adaptive immunity^75^; and bone loss is a major factor in the pathogenesis of osteoporotic fractures^76^. Hence, our findings point to age-related gains in DNAme as contributors to the etiology of common diseases in old age. In physiological aging, gradual DNA hypermethylation by WT DNMT3A may act through the interaction of DNMT3A1’s N-terminal region with H2AK119ub^21,22^ (model, Extended Data Fig. 10g). Cumulative hypermethylation would then be predicted to contribute to similar tissue pathologies in later life as those seen in HESJAS. Notably, differing DNAme rates across species correlate with longevity^77^, raising the possibility that altered DNMT3A activity, as well as contributing to human tissue pathologies, could affect mammalian lifespan. Our findings also provide experimental evidence supporting epidemiological associations between DNA hypermethylation, morbidity and mortality^5,69^. Genes encoding DNMT3A and opposing DNA and histone demethylases^12–14^ have GWAS associations with metabolic, bone and hematological traits^78^. Hence, polymorphisms in these genes may account for aspects of aging in human populations.

Our work on *Dnmt3a*^W326R/+^ hematopoiesis suggests that age-related hypermethylation reduces regenerative capacity and increases lineage skewing in BM, and would similarly predict hypermethylation to reduce mesenchymal progenitor output, leading to reduced osteoblast and adipocyte numbers. Consequent decreases in bone formation and adipose tissue reservoirs would then result in osteoporosis and insulin resistance with dyslipidemia, respectively. Previous experimental approaches to address the role of age-related DNAme at Polycomb sites in HSCs were confounded by co-occurrence of DNA hypomethylation or induction of DNA damage^79,80^. Here HESJAS gain-of-function mutations in DNMT3A specifically cause DNA hypermethylation in HSCs, providing a direct link between hypermethylation in these regions and stem cell dysfunction.

How might hypermethylation negatively impact stem cell output? A role for Polycomb in HSC self-renewal and hematopoiesis has been established through knockout and overexpression studies^81–83^. However, loss of core Polycomb proteins leads to derepression of Polycomb-target genes and activation of pro-apoptotic factors, resulting in depletion of HSC or progenitor numbers and early failure of postnatal hematopoiesis. Here the consequences of replacing Polycomb marks with DNAme in *Dnmt3a*^W326R/+^ HSCs are distinct—HSC and early progenitor numbers remain constant and Polycomb-target genes are not de-repressed, with few transcriptional differences seen during HSPC differentiation. The dogma is that DNA promoter methylation is repressive, so DNA hypermethylation, substituting for Polycomb, may act as an alternative repressive mark. Our transcriptomic analyses indicate that DNA hypermethylation could then impair transcriptional activation dynamics during differentiation. Specifically, hypermethylation of *Pax5*, and reduced activation of this gene during B cell development, implicates it in *Dnmt3a*^W326R/+^ B cell lymphopenia. Dnmt3a may also have noncatalytic functions^84^, meaning that effects of HESJAS mutations might be independent from observed DNAme gains. So, future experiments will be necessary to confirm that Pax5 methylation directly accounts for reduced lymphocyte number. Nevertheless, the strong selection against *Pax5* hypermethylation in late stage differentiation and mature B cells (Fig. 5g,h) argues in favor of a direct effect of DNA hypermethylation.

The *Dnmt3a*^W326R/+^ mouse, as an epigenetic progeroid mouse model, should in future provide further insights into the cellular mechanisms and phenotypic consequences of age-related gains in DNAme. In addition, it could provide a preclinical model for development of anti-aging therapies through epigenetic reprogramming.

In conclusion, we connect DNA hypermethylation to accelerated age-related pathologies in man and mouse, addressing a key knowledge gap in the field.

## Methods

### Research participants

Participants were recruited to the research studies by their local clinician. P1–P3 were previously described^15^. The research studies were approved by the Scottish Multicentre Research Ethics Committee (05/MRE00/74), the University of Utah medical review board (IRB00085855), the Bergen Hospital Trust ethical review board and the Ethics Committee of the Instituto de Salud Carlos III (CEI PI 93_2022). Informed written consent was obtained from all participating families. Families provided written consent for the publication of clinical photographs. Diagnostic exome sequencing was performed on P4, P5, P6, P7, P9, P10 and P13, whole-genome sequencing on P8 and P11 and custom NGS gene panel on P12 to identify pathogenic variants in DNMT3A (accession number NM_175629.2, NP_783328.1).

### Structural modeling

HESJAS DNMT3A mutations were modeled as described^15^. Using the crystal structure of the DNMT3B PWWP domain bound to H3K36me3 (Protein Data Bank ID: 5CIU) and visualized with PyMol (v2.4.0). The residues associated with HESJAS DNMT3A mutations are conserved between DNMT3A and DNMT3B, apart from DNMT3A L300, corresponding to I233 in DNMT3B. For visualization, I233 in the DNMT3B crystal structure was mutated to leucine using PyMol’s mutagenesis function.

### Infinium Methylation BeadChip assays

Human Infinium Methylation EPIC BeadChip and Infinium Mouse Methylation BeadChip assays were performed as described^15^. For mouse tissues, genomic DNA was extracted using a QIAamp UCP DNA micro kit (QIAGEN) using the manufacturer’s cell extraction protocol for BM, and tissue-specific protocol for intestine. DNA was bisulfite converted using the EZ DNA Methylation kit (Zymo Research, Infinium assay protocol). Infinium BeadChip assays (Illumina) were performed according to the manufacturer’s instructions by Edinburgh Clinical Research Facility using an Illumina iScan. For analysis, the minfi package (v1.50.0) was used to process raw Infinium idat files (‘single-sample normal exponential out of band’, ssNoob method)^87,88^ to *β* values. IlluminaMouse-Methylationanno.12.v1.mm10 (https://github.com/chiaraherzog/IlluminaMouseMethylationanno.12.v1.mm10) was used to annotate probes to mm10.

### EM-seq

BM was flushed from both tibia and femur, and HSCs were isolated by LSK-SLAM FACS sorting (‘Cell sorting’). Genomic DNA from HSCs was isolated with QIAamp UCP DNA micro kit (QIAGEN) and quantified by Qubit dsDNA High Sensitivity Assay (Thermo Fisher Scientific). DNA from three individual mice was pooled to acquire sufficient genomic DNA for library preparation. Libraries were prepared from 0.5 to 1 ng of each sample using the NEBNext Enzymatic Methyl-seq Kit (NEB, E7120) according to the manufacturer’s instructions for low-input samples. Briefly, DNA controls were spiked in—unmethylated λ DNA and CpG-methylated pUC19 DNA. Controls were diluted 1:500 and 1 ml of the relevant diluted controls was added to each DNA sample. Then, DNA samples were sheared to an average size of 240–290 bp using Covaris E220 Evolution Focused-Ultrasonicator (Covaris, 3814). Sheared DNA samples were end-repaired, 5′ phosphorylated and 3′ dA-tailed. After adaptor ligation, DNA fragments were purified with NEBNext Sample Purification Beads followed by EM-seq two-step conversion. Fragments were then purified with AMPure XP beads (1× volume) before PCR with unique dual-index sequences to selectively enrich DNA fragments with adaptor sequences and to allow multiplex sequencing. Libraries were quantified by Qubit. Sequencing was performed on NextSeq 2000 (Illumina, 20038897) using P3 Reagents (20040560).

Sequencing quality was assessed with FASTQC (v0.11.4); low-quality reads and remaining adaptors were removed using TrimGalore (v0.4.1; settings: ‘--adapter AGATCGGAAGAGC --adapter2 AAATCAAAAAAAC’). PCR duplicates were identified and removed using Bismark’s ‘deduplicate_bismark’ command. Aligned BAM files were processed to report coverage and the number of methylated reads for each CpG observed. Forward and reverse strands were combined using Bismark’s ‘methylation extractor’ and ‘bismark2bedgraph’ modules with custom Python and AWK scripts. Processed EM-seq files were assessed for conversion efficiency based on the proportion of methylated reads mapping to the CpG-methylated pUC19 DNA (>96% in all cases) and λ genome spike-ins (<0.5% in all cases). BigWigs for visualization of EM-seq data were generated using BEDTools (v2.30.0)^89^ and converted to bigWigs using UCSC tools ‘bedGraphToBigWig’ (v369). Windows with a total coverage <5 were excluded before conversion to bigWigs.

### Enzymatic methyl conversion PCR analysis

Genomic DNA (200 ng) was converted with NEBNext Enzymatic Methyl-seq Conversion Module (New England Biolabs, E7125) following the manufacturer’s instructions, with AMPure XP magnetic beads used for clean-up steps. Converted DNA was PCR amplified using EpiTaq (Takara) and custom-made locus-specific primers—Pax5-FW (GGT AGT TGA TTA TTG AGT TGA AAT TAA), Pax5-RV (TTA TAC TAA TAC AAA ATT CAA AAC CAC T), Hoxc13-FW (TTT TTA AAA AGT TGG AGT AGA TTA TGT T), Hoxc13-RV (TTA AAA AAA ACC CAA TAC TAA TAA CCT T), Hoxd13-FW (AAA GAA AAG AAA AGA AAA GAA AAG AAA A) and Hoxd13-RV (TAA AAA CCT ATT ACT CAT CTT TCC TTA A). PCR products were purified with QIAGEN QIAquick PCR Purification kit (28104). Sequencing was performed by Plasmidsaurus using Oxford Nanopore Technology with custom analysis and annotation. For analysis, FASTQ files were converted to FASTA files with seqtk (v1.4 with seq -A). Read alignment information and DNAme were retrieved using Quma (-f 3). Downstream processing and plotting were done using R (v4.4.1).

### DMR identification

For Infinium arrays, DMR calling was performed as described^15^—DMRs were called on the basis of ≥2 probes in a window having a difference in *β* value of at least 0.2 between individual DNMT3A^GOF^ and DNMT3A^WT^ samples, changed in the same direction. DMR methylation level was defined as the mean *β* value of all probes located in the DMR.

For EM-seq, DMRs were called using the DSS package (v2.54.0)^90–93^ on the basis of ≥5 CpGs in a window having a difference in methylation values of at least 0.1 with the following parameters: p.threshold = 0.01; minCG = 5; delta = 0.1; pct.sig = 0.9. Only CpGs present in all six samples were considered for DMR calling (*n* = 18,482,064). For enrichment analyses, the set of DMR CpGs was compared to a genome-wide background control set of all CpGs sequenced. DMR methylation level was defined as the weighted mean methylation level (methylated coverage/total coverage) using all CpGs observed within the DMR.

### GO enrichment analysis

GO enrichment analysis was performed as described^15^. For human EPIC array called DMRs, control and DMR-associated probes were mapped to their closest annotated TSS with the ChIPpeakAnno package (v3.40.0)^94^, using Ensembl annotated protein coding genes (Ensembl Release 75/GCRh37), using the ‘annotatePeakInBatch’ command, with setting—FeatureLocForDistance = ‘TSS’ and output = ‘shortestDistance’, to derive DMR and control Ensembl gene ID lists. For mouse EM-seq, DMRs were mapped to genes if overlapping either 1 kb upstream of the TSS or within the gene body. Gene lists were mapped to the Biological Process and Molecular Function GO terms using Ensembl Biomart. All parental terms were identified using custom R scripts and the GO.db package (v3.20.0). For each GO term, statistical enrichment for term-associated genes in the DMR list versus control list was tested by Benjamini–Hochberg-corrected Fisher’s exact test, with a false discovery rate of 0.05. Terms with <10 genes in control list were excluded from analysis. GO terms were filtered for semantic similarity (Bioconductor packages GOSemSim (v2.32.0)^95^ and org.Hs.eg.db (v.3.20.0) or org.Mm.eg.db (v.3.21.0)). Similar terms were defined on the basis of Wang similarity of >0.7, using a modified ‘simply’ function from the ‘clusterProfiler’ package^96^. For each group, the term with the lowest adjusted *P* value was retained.

### ChromHMM enrichment analysis

Analysis performed as described^15^. EPIC Infinium probes and EM-seq CpGs were respectively mapped to existing ChromHMM annotations^40,97^ using the BEDTools intersect function (v2.30.0)^89^. Enrichment was assessed by Fisher’s exact tests comparing number of hypermethylated DMR probes/CpGs per category against the total number of probes/CpGs in that category.

### DMV definition

Previously reported human DMVs^19^ were mapped to hg19 using the UCSC LiftOver tool, and merged DMV regions from all five cell types were determined using the BEDTools ‘merge’ function. For mouse, DMVs were called based on EM-seq *Dnmt3a*^+/+^ HSC data mapped to mm10, using similar parameters to those described previously^19^. Mean DNAme level calculated using BEDTools ‘map’ for 1 kb window intervals generated using BEDTools ‘makewindows’. Then, mean DNAme was computed for 5 kb sliding windows with 1 kb steps, 5 kb windows with CpG coverage and DNAme level below 15% were called as DMVs. Overlapping windows were merged into a final list of DMVs.

### DNAme and histone modifications at DMVs

Scaled heatmaps and metaplots of human and mouse H3K27me3 and H3K4me3, and mouse HSC EM-seq data at DMVs were generated by defining 100 windows across each DMV using BEDTools (v2.30.0)^89^. For histone PTMs, the level in each window per sample was defined as the mean count within that window. For mouse HSCs, the methylation level in each window per sample was defined as the mean %mCpG within that window. Flanking regions were plotted using 40 250 bp windows extending 10 kb upstream and downstream of each region. Regions without coverage in all samples were excluded from the analysis. For visualization, the mean of two and three independent replicates per window was used for histone PTM and HSC EM-seq, respectively.

DNAme of human whole-genome bisulfite sequencing (GSE31263) was generated using ‘computematrix’ from deepTools (v3.5.4)^98^. All DMVs were normalized to a size of 20 kb based on their start and endpoints using window size of 250 bp. Flanking regions were plotted using the same window size and a maximum distance of 10 kb (-m 20000; --binsize 250; -a 10000; -b 10000). Heatmaps and metaplots were generated in R. Color scales for heatmaps range from the minimum to the 90% quantile of the normalized read count for the reference dataset in each set of heatmaps. Polycomb-positive DMVs were classified based on mean H3K27me3 level; H3K27me3 mean level across DMVs displayed a bimodal distribution and therefore the local minimum between two peaks was set as the threshold for Polycomb-positive DMVs.

### *Dnmt3a*^W326R^ mice

Mice carrying the W326R point mutation in Dnmt3a (accession number NM_007872.5, NP_031898.1) were generated on a C57Bl/6JCrl background by The University of Edinburgh Bioresearch & Veterinary Services (BVS) Central Transgenic Core Facility, using CRISPR–Cas9 microinjection of fertilized oocytes as described^15^. The line was maintained by breeding *Dnmt3a*^W326R/+^ males with C57Bl/6JCrl females purchased from Charles River Laboratories. Mice were cohoused in groups of two to six littermates of mixed genotype in individually ventilated cages at the Bioresearch & Veterinary Services research facility at The University of Edinburgh. A 12-h light/12-h dark cycle (lights on at 7.00 and off at 19.00) and controlled temperature/humidity (21 °C/50%) were maintained. Mice had ad libitum access to standard chow (RM1, Special Diet Service). All experimental protocols were approved by The University of Edinburgh’s Animal Welfare and Ethical Review Board and performed under a UK Home Office-approved Project License, in compliance with the UK Home Office Animals (Scientific Procedures) Act 1986. For phenotyping, investigators were blinded to genotypes at the point of data collection.

### Survival curve

Survival data were collected from our colony throughout the period of this study. Events were recorded for spontaneous deaths (found dead in cage) and upon termination at humane endpoints when mice were at risk of exceeding moderate severity, with criteria defined by the UK Home Office used by their carers for twice daily observations/scoring. These criteria are a measure of poor overall health and include changes in appearance (for example, coat staring, hunched posture, hair loss), behavior (for example, change in temperament, reluctance to move) and other clinical signs (for example, reduction in body weight, diarrhea, abnormal breathing). WT littermates were similarly monitored from 6 months of age and terminated when moderate severity was reached. Any mice terminated for other reasons were censored from time of termination.

### Neurobehavioural tests

Individual pedestrian locomotion was measured over a 48-h period in temperature-controlled metabolic chambers (Promethion Core system, Sable Systems), with a 12-h light/12-h dark cycle and free access to food and water. Physical activity measurements (XYZ beam breaks) were collected in 3-min intervals. To allow acclimatization, mice were single housed for 1 week before measurements started. Data were analyzed in CalR2 (ref. 99).

Open field tests were performed in a Perspex box (W × D × H = 50 ×50 × 40 cm), with three opaque white sides and one transparent side, to allow simultaneous top-down and side-on video recording using HD C920 webcams (Logitech). The arena was illuminated from above using a 4,000 K 40 W equivalent LED bulb. After habituating to the recording room for at least 30 min, mice were collected from their home cages through tube handling and placed in the center of the arena. Mice were recorded as they freely explored the arena for 20 min, with experimenters out of view of the animals. The arena was cleaned across recordings using disinfectant spray (Safe4), followed by distilled water, then wiped dry. Top-down videos were analyzed using ezTrack (v1.2)^100^ using the following tracking parameters in batch mode: ‘loc_thresh’ = 99.5, ‘use_window’ = True, ‘window_size’ = 100, ‘window_weight’ = 0.5, ‘method’ = ‘abs’, ‘rmv_wire’ = True, ‘wire_krn’ = 5. A 30 × 30 cm square in the center of the arena was digitally annotated in ezTrack for thigmotaxis measurements. The total distance traveledover the 20-min recording and the percentage of time spent in the 30 × 30 cm center zone were calculated for each animal.

Grip strengths were measured using an electronic grip strength meter (Bioseb). Animals were suspended by the base of the tail and allowed to grip the center of the grid with either their forelimbs or with all four limbs. Animals were then pulled backward across the grid until their grip failed. The maximum force exerted over the trial (*n*) was recorded. First, the grip strength of the forelimbs was measured thrice back to back. Animals were then given a short break before the grip strength of all limbs was measured thrice back to back. Average forelimb and all-limb grip strengths were calculated for each animal.

Spatial reference memory was assessed in the Y-maze test. The Y-shaped arena consisted of three arms of identical length at 120° angles. Mice were placed at the end of one arm (facing the end) and allowed to explore for 5 min. The Alternation Index (alternation index = number of alternations/max alternations × 100) was determined by manual scoring, where an animal was considered to have entered an arm of the maze when all four paws were inside.

### scRNA-seq

HSPCs were isolated by FACS (‘Cell sorting’). Single cells were processed through the 10x Genomics Chromium Platform using the Chromium Next GEM Single Cell 3′ GEM, Library & Gel Bead Kit (v3.1; 10x Genomics, PN-1000121), the Chromium Next GEM Chip G Single Cell Kit (10x Genomics, PN-1000120) and Single Index Kit T Set A (10x Genomics, PN-100021) as described^101^.

Raw reads were mapped to mm10 and quantified using the Cell Ranger pipeline (v6.0.1) with default parameters. Cell-associated barcodes were determined using the EmptyDrops method^102^ implemented in the Cell Ranger pipeline and background-associated barcodes excluded. Subsequent data analysis was performed using Scanpy^103^. Cell libraries with less than 2,000 detected genes or with mitochondrial gene expression exceeding 10% UMI counts were removed from downstream analysis. Multiplets were identified using the Python package Scrublet^104^ and removed. Remaining cells that passed quality controls (*n* = 7,555) were log-normalized and further processed using the PMCA pipeline^105^ to compute UMAP embeddings and identify different HSPC populations.

Differential abundance analysis was performed using the Python package MELD^106^, implemented in the PMCA pipeline^105^. Briefly, a cell similarity graph was constructed based on the Euclidean distances in the 50-dimensional principal component space, and a kernel density estimate (KDE) was computed for each sample. To account for absolute cell abundance changes, we applied a scaling factor to the mutant KDE, calculated as the ratio between the median WT HSC KDE and median mutant HSC KDE, as our observations showed no significant change in absolute HSC cell number in *Dnmt3a*^W326R/+^ mice (Fig. 3c). Differential abundance was then quantified by performing cell-wise L1 normalization of mutant and WT KDEs.

For differentiation trajectory analysis, cellular fate probability was inferred using Python package CellRank^107^, implemented in the PMCA pipeline^105^. The erythroid trajectory was defined as cells belonging to ‘HSC’, ‘MPP’, ‘MEP’, ‘Early Ery prog’, ‘Mid Ery prog’ or ‘Late Ery prog’ clusters and with erythroid fate probability of ≥0.10. The myeloid trajectory was defined as cells belonging to ‘HSC’, ‘MPP’, ‘Neut prog’ or ‘Mono/DC prog’ clusters. Diffusion pseudotime was computed using Scanpy function ‘tl.dpt’ and cellular density along pseudotime was computed using SciPy^108^ function ‘gaussian_kde’. To account for absolute cell abundance, mutant cell density was scaled using the same scaling factor calculated for differential abundance analysis.

For differential expression analysis, low-expression genes with mean expression of ≤0.05 counts per 10,000 UMIs (CP10K) were removed for each cell type. Wilcoxon rank-sum test was performed using Scanpy function ‘tl.rank_genes_groups’, and genes with |log_2_(FC) | ≥0.5 and Benjamini–Hochberg-adjusted *P* values of <0.05 were considered significant.

### RT–qPCR analysis of *Pax5* expression

RNA (50 ng) from each B cell fraction was converted to cDNA using SuperScript III first-strand synthesis (Invitrogen) with random hexamer primers. *Pax5* expression was assessed by qPCR using SYBR Select master mix (Applied Biosystems) following the manufacturer’s recommendations (Ta = 56 °C, 45 cycles), with primers Pax5-FW (ACC ATC AGG ACA GGA CAT GG) and Pax5-RV (GCG GAC TAC ATC TGG GAG TG). Expression was normalized to the *Hprt1* housekeeping gene with primers Hprt1-FW (CAG TCC CAG CGT CGT GAT TAG) and Hprt1-RV (GTG ATG GCC TCC CAT CTC CTT).

### VDJ-seq

Pro-B cells from 3 *Dnmt3a*^*+/+*^ and 3 *Dnmt3a*^W326R/+^ mice were isolated by FACS (‘Cell sorting’). Genomic DNA was isolated as described^109^ with minor modifications. Equal volume of 2× lysis buffer (10 mM Tris–HCl pH 8.0, 1 mM EDTA, 4% SDS and 200 μg ml^−1^ proteinase K; New England Biolabs, P8107S) was added to cell suspensions, followed by 30-min incubation at 55 °C with agitation (700 rpm). Next, RNase A (Thermo Fisher Scientific, EN0531) was added at 20 μg ml^−1^, followed by 30-min incubation at 30 °C with agitation. After phenol–chloroform extraction, ethanol precipitation was performed, using linear polyacrylamide (Thermo Fisher Scientific, J67830.XF) as a carrier.

This DNA (67–500 ng per sample) was used for VDJ-seq library preparation as described^110^, but modified for analysis of V_H_ recombination only. DNA was sheared to 500 bp (Covaris, E220; Focused-Ultrasonicator), and processed with NEBNext Ultra II End Repair/dA-Tailing Module (New England Biolabs, E7546). For subsequent adaptor ligation, 30 μl of Ligation Master Mix and 1 μl of Ligation Enhancer, along with 1.25 μl P7 adaptor F1 R1 mix (15 μM) and 1.25 μl P7 adaptor F2 R2 mix (15 μM), were added to the previous 65 μl reaction, followed by 2-h incubation at 20 °C. Adaptors were made with P7 adaptor F1, P7 short adaptor R1-block, P7 adaptor F2 and P7 short adaptor R2-block^110^. Clean-up was performed with 1× volume Agencourt AMPure XP beads (Beckman Coulter, A63881), eluting the DNA in 25 μl EB (10 mM Tris–HCl, pH 8.5). Primer extension was then performed in 50 μl reactions using Vent (exo-) DNA polymerase kit (New England Biolabs, M0257) and biotinylated J primers (Mouse_JH1_Rev_Bio, Mouse_JH2_Rev_Bio, Mouse_JH3_Rev_Bio and Mouse_JH4_Rev_Bio) as described^110^. Samples were purified using QIAquick PCR Purification Kit and eluted in 40 μl EB; primer extension products were captured using 5 μl Dynabeads MyOne streptavidin T1 beads (Invitrogen, 65601) as described^110^, and bead-captured products resuspended in 10.5 μl EB. PCR amplification was then incorporated with P5 adaptor using P7 short adaptor primer and J-P5 short primer mix^110^ (25 μl reactions, Q5 High-Fidelity 2× Master Mix; New England Biolabs, M0492S; 19 cycles). After PCR, Dynabeads were removed, AMPure XP clean-up performed and PCR products eluted in 10.5 μl EB. Next, incorporation of flow-cell-binding and barcoding sequences was performed by PCR (25 μl reactions, Q5 High-Fidelity, six cycles) with P7 flow-cell and P5-I5 flow-cell index primers^110^. P5-I5_flow_cell_index_2, 5, 6, 12, 15 and 19 were used tobarcode our six samples. PCR products were purified using two tandem AMPure XP beads clean-up steps, using 15 μl for final elution. Barcoded sublibraries were quantified using Agilent High Sensitivity DNA Assays on an Agilent 2100 Bioanalyzer. Equimolecular amounts of each were pooled to create a final library for paired-end sequencing (2 × 300 bp; Illumina MiSeq). Sequencing and analysis were performed as recommended^110^.

### DNAme aging clocks

CpG sites within DMR start/end coordinates were extracted from previously generated DNAme data within the GS population study^111^. Quality control and normalization of GS Illumina EPICv1 array data were applied separately to four subsets as described^111^. Briefly, poorly performing probes (based on signal detection *P* value and bead count) were removed from each subset, along with samples with a high proportion of such probes, and samples whose methylation-predicted sex did not align with participants’ self-reported sex. Each subset was then normalized using the Dasen method^112^. A single DNAme superset was generated by applying Dasen normalization to all subsets, after filtering on the intersection of their QC-passing probes. The relationship between chronological age in GS and DNAme at each CpG site was assessed through Pearson’s correlation coefficient in the superset. Epigenetic clocks were generated through penalized regression models in the R glmnet package (v4.1-8), using the largest of the four subsets (set 4, *n* = 8,898) as a training sample for an elastic net model, setting the mixing parameter (*α*) to 0.5. DNAme-derived estimates of chronological age were obtained in the largest of the remaining subsets (set 1, *n* = 5,085). These estimates were calculated as the weighted sum of scores from CpGs selected from the elastic net models. Model performance was assessed through Pearson’s correlation coefficient and median absolute error.

Chronological age prediction for patients with HESJAS by DNAme epi-clocks was done using the ‘methylclock’ (v.1.110.0) R package^113^. For mouse HSC age prediction, an epi-clock from ref. 71 was used.

### Metabolic analyses

Nonfasting blood glucose concentrations were assessed through tail bleeding using an AccuChek Performa Nano. Terminal bleeds were performed by cardiac puncture under general anesthesia; blood collected in EDTA-coated tubes, placed on ice and centrifuged at 4 °C for 2 min at 12,000*g* to separate plasma before storage at −80 °C. Postmortem tissues were collected, weighed and sectioned before fixation in 10% paraformaldehyde or snap freezing and storage at −80 °C. Plasma biochemical analyses were conducted by the Core Biochemical Assay Laboratory at Cambridge University Hospitals NHS Foundation Trust. A Siemens Dimension EXL autoanalyzer was used to quantify triglyceride, total cholesterol and HDL-C. LDL-C was calculated from triglyceride, HDL-cholesterol and total cholesterol concentrations using the Friedewald formula (LDL-C = total cholesterol − HDL-C − (triglycerides/2.2)). Insulin, adiponectin and GDF15 were quantified by electrochemiluminescence immunoassay with measurements read on the MesoScale Discovery Sector s600. Reagents and kits used were as follows: triglycerides (DF69A) and cholesterol (DF27) from Siemens Healthcare; high-density lipoprotein (CH2849) and low-density lipoprotein (CH2656) from Randox; and insulin (K15124C-3), adiponectin (K152BYC-2) and GDF15 (K152APNR-2) from Meso Scale Discovery.

### Histological analysis of liver and adipose tissue

Formalin-fixed tissues were stored in 70% ethanol for 2 days before dehydrating and paraffin embedding, cutting of 5 μm sections and staining with hematoxylin and eosin (H&E) using standard procedures^114^. Liver and adipose tissue slices were scanned and imaged with a Zeiss Axioscan.Z1 and Zen2.6 software. Images were analyzed at ×20 magnification using Fiji.

Fresh frozen tissue was sectioned at 8-μm thickness, rinsed with 60% isopropanol and stained with 0.5% Oil Red O solution (Sigma, 1320-06-5) for 15 min. Sections were then rinsed again with 60% isopropanol, counterstained with Harris hematoxylin for 1 min, washed with distilled water and finally mounted in an aqueous medium. Images were acquired as above, and lipid staining was quantified by measuring the proportion of the total image area stained.

### µCT analysis

μCT analysis was performed using a Neoscan N80 system (Neoscan) and analyzed as described^115^. For assessment of trabecular bone morphometry, femurs dissected free of most soft tissue were fixed for 24 h in buffered 4% paraformaldehyde, transferred to 70% ethanol and scanned at a resolution of 5 μm (67 kV, 200 μA, rotation step size 0.4°, using a 0.5 mm aluminum filter, camera at 2 × 2 binning, averaging at 2). Reconstruction was performed using Neoscan N80 software (v3.1.2). Trabecular parameters were measured using CTAn software (v1.20.8.0; Bruker Skyscan) in a stack of 200 slices of 200 μm proximal to the growth plate in distal femurs. Cortical parameters were measured using Skyscan CTAn software in a stack of 100 slices at the midshaft of the femurs. Trabecular bone was separated from cortical bone using a CTAn macro, and cortical and trabecular parameters were then measured according to ASBMR guidelines. To account for reduced femur length in mutant mice (12.1% in males and 14.5% in females), the height of the image stack analyzed was reduced by 14%, resulting in 172 slices for trabecular bone and 86 slices for cortical bone. The distance of the trabecular image stack to the growth plate was also decreased by the same percentages. For analysis of bone mineralization, scans were calibrated using hydroxyapatite standards scanned in the same sample holders and under the same conditions as the bone samples.

### Three-point bending test

The hind limbs were wrapped in moist paper and stored at −20°C until scanning. Samples were defrosted at 4 °C overnight. Next, femurs were scanned at a resolution of 12 μm with other settings as described above and the bone length measured in Skyscan Dataviewer (v1.5.6.6; Bruker Skyscan). Immediately after completion of μCT scans, flexural strength of femurs was assessed by dynamic three-point bending test using a ZWICK-mechanical testing machine (ZWICK), using a span of 6 mm between the support points and a crosshead speed of 1 mm min^−1^.

### Histological analysis of skin tissue

Skin samples from the dorsum of 6-month-old males were fixed in 4% paraformaldehyde in PBS for 24 h at 4 °C, then transferred to 70% ethanol. Tissue was processed using a Tissue-TeK VIP infiltration Processor (Sakura) for paraffin embedding, and sections were cut at 5 μm on a Leica microtome for H&E staining. Images were digitized using a NanoZoomer Digital slide scanner and NDP.view2 software (Hamamatsu), and images were analyzed using QuPath (v0.5.1)^116^. Magnification was set to fivefold for hair follicle and subcutaneous adipose tissue, and tenfold for epidermal thickness annotations; and measurements were performed using the linear annotation object to measure epidermal thickness, thickness of subcutaneous adipose tissue, and hair follicle length (from outer epidermis to base of follicle). Analysis was conducted by a single blinded observer using the ‘Mask image names’ facility in QuPath. All follicles were visualized, and bulb-to-epidermal length in section was quantified for every follicle in which the full length was captured in all 16 slides; for epidermal thickness, five representative sites were measured; for thickness of subcutaneous adipose tissue, ten representative sites were measured.

### Histological analysis of knee joints

Right hind limbs were fixed in 4% paraformaldehyde in PBS overnight and decalcified in 10% EDTA in PBS for approximately 2 weeks at 4 °C, then processed and embedded in paraffin and coronally cut into 5 μm sections, followed by staining with Safranin O and Fast Green (S&F) or H&E using standard protocols. Semi-quantitative scoring of cartilage damage at the tibiofemoral articulation was performed by a blinded observer using the OARSI scoring system^117^ on images of tibial plateau and femoral condyle articular cartilage. Eight S&F-stained sections at 50 μm intervals were analyzed for both medial and lateral compartments, yielding 32 scores per mouse, which were averaged to give an overall cartilage damage score on a scale of 0 to 6. Thickness of the proximal tibial growth plate was calculated by dividing growth plate area by width, measured using QuPath (v0.6.0)^116^. For each mouse, average growth plate thickness was calculated from three S&F-stained sections at 100 μm intervals. Marrow adiposity was determined in medial and lateral anterior metaphysis of the distal femur by semi-automated analysis of two H&E-stained sections, 100 μm apart, using the MarrowQuant 2.0 algorithm^118^ in QuPath (v0.5.1)^116^, with manual correction of artifacts caused by tissue shrinkage and blood vessels mistaken for adipocytes.

### Flow cytometry

Flow cytometry analyses on PB or BM were performed as follows. For PB, two to three drops per animal were collected through tail vein into PBS with 0.2 mg ml^−1^ EDTA. For BM, femur and tibia were flushed with cold PFP solution (PBS, 10% FBS, 1% penicillin–streptomycin). Red blood cell lysis was performed using ACK lysing buffer (Gibco, A1049201) or IOTest 3 Lysing Solution (Beckman Coulter, A07799). Cell suspensions were stained for 25 min at 4 °C in the dark with various combinations of antibodies.

Analyses of steady-state BM cells of nontransplanted animals were performed as follows. For differentiated cells, the panel was CD4 Biotin (1:1,600; BD Biosciences, 553648), CD8a Biotin (1:800; BD Biosciences, 553028), CD11b BV421 (1:50; BioLegend, 101223), B220 BV786 (1:200; BD Biosciences, 563894), CD71 FITC (1:100; Invitrogen, 11-0711-82) and Gr-1 PE (1:200; BD Biosciences, 551461). After removing excess primary antibody solution by washing with PFP, cell suspensions were incubated with Streptavidin APC (1:200; Invitrogen, 17-4317-82) on ice for 20 min. LIVE/DEAD Fixable Yellow Dead Cell Stain (Thermo Fisher Scientific, L34959) was used to exclude dead cells. For HSC and progenitor cell populations, the LSK-SLAM panel used is described in ‘Cell sorting’.

Analysis of Hardy fractions within immature B cell populations from BM was performed with antibody panel comprising B220 AF700 (1:65; BioLegend, 103232), IgM FITC (1:100; Invitrogen, 11-5790-81), BP-1 PE (1:50; Invitrogen, 12-5891-81), CD19 PE-Cy7 (1:200; BD Biosciences, 552854) and CD43 APC (1:200; BD Biosciences, 560663). After removing excess primary antibody solution by washing with PFP, cells were fixed using BD Cytofix/Cytoperm (BD Biosciences, 554714). Fixed cells were stained with DAPI (1 μg ml^−1^ in 100 μl per 3 × 10^6^ cells; 10 min on ice), washed in PFP and data acquisition performed on a Beckman Coulter Cytoflex SRT (4 laser) using Beckman Coulter CytExpert SRT software (v1.1.0.10007).

All other Flow Cytometry experiments were performed on a 5-laser LSRFortessa SORP Cell Analyzer or BD FACSAria II (BD Biosciences), with gates set using unstained WT and FMO controls, and data acquired using BD FACSDiva software. Downstream analyses were performed using FlowJo (v10).

### Cell sorting

To isolate HSCs for EM-seq, BM cells were stained using the following LSK-SLAM panels: CD4 Biotin (1:1,600; BD Biosciences, 553648), CD5 Biotin (1:800; BD Biosciences, 553018), CD8a Biotin (1:800; BD Biosciences, 553028), CD11b Biotin (1:200; BD Biosciences, 557395), B220 Biotin (1:200; BD Biosciences, 553086), Ter119 Biotin (1:50; BD Biosciences, 553672), Gr-1 Biotin (1:100; BD Biosciences, 553125), Sca1 FITC(1:200; BioLegend, 122506), CD48 Pe (1:600; BD Biosciences, 103405), CD150 Pe-Cy7 (1:200; BD Biosciences, 115914) and c-KIT APC (1:200; BD Biosciences, 105812). After removing excess antibody solution by washing in PFP, cell suspensions were incubated with Streptavidin Pacific Blue (1:200; Molecular Probes, S11222), before sorting on a BD FACSAria II (BD Biosciences). DAPI stain was used for dead-cell exclusion. To isolate HSCs for transplant experiments, BM was flushed from femur and tibia into PFP (PBS, 10% FBS, 1% penicillin–streptomycin), red blood cells lysed with ACK lysing buffer (Gibco, A1049201) or IOTest 3 Lysing Solution (Beckman Coulter, A07799) and cells manually counted. Cell suspensions were stained for 25 min at 4 °C in the dark with an LSK-SLAM antibody panel—CD3 PE (1:1,000; BioLegend, 100205), B220 PE (1:1,000; BD Biosciences, 553089), Gr-1 PE (1:1,000; BD Biosciences, 553128), Ter119 PE (1:1,000; BD Biosciences, 553673), CD11b PE (1:1,000; BD Biosciences, 553311), NK1.1 AF700 (1:1,000; BioLegend, 108730), Sca1 PeCy (1:250; eBioscience, 25-5981-81), CD48 AF700 (1:250; BioLegend, 103425), c-KIT BV421 (1:250; BD Biosciences, 562609) and CD150 BV605 (1:100; BioLegend, 115927). Cells were washed with PFP, and HSCs (Lineage^−^ c-Kit^+^ Sca1^+^ CD48^−^CD150^+^) isolated using FACS on a 5-laser LSRFortessa SORP Cell Analyzer (BD Biosciences). Dead-cell exclusion was performed using Hoechst 33342. Sorted cells were collected in sterile 50% FBS in PBS for transplantation. The same approach and panel were used to sort HSPCs (Lin^−^cKit^+^) for scRNA-seq.

Immature B cell Hardy fractions were sorted from mouse BM for methylation analysis and *Pax5* RT–qPCR using Ter119 BV421 (1:200; Invitrogen, 404-5921-82), CD4 BV421 (1:1600; Invitrogen, 404-0042-82), CD8 (1:200; Invitrogen, 404-0081-82), NK1.1 BV421 (1:40; BioLegend, 108741), Gr-1 BV421 (1:200; BD Biosciences, 562709), CD19 BV650 (1:20; BioLegend, 115541), B220 BV786 (1:200; BD Biosciences, 563894), IgM FITC (1:100; Invitrogen, 11-5790-81), BP-1 PE (1:50; Invitrogen, 12-5891-81) and CD43 APC (1:200; BD Biosciences, 560663). DAPI stain was used to exclude dead cells. Hardy fractions A, B, C/D (fractions C and D combined) or C–F (fractions C, D, E and F combined) were isolated using a Beckman Coulter Cytoflex SRT (4 laser) and collected into sterile PBS.

For VDJ-seq, BM cells were stained using B220 APC (1:200; BioLegend, 103211), CD43 Pe-Cy7 (1:200; BD Biosciences, 562866) and IgM PerCP-eFluor710 (1:200; Thermo Fisher Scientific, 46-5790-82). DAPI, added immediately before FACS acquisition, allowed dead-cell exclusion. Approximately 50,000 Pro-B cells (IgM^−^ B220^+^ CD43^+^) per mouse were sorted into PBS.

For all other FACS experiments, gates were set using unstained WT and FMO controls, data acquired using BD FACSDiva software and downstream analyses performed using FlowJo (v10).

### BM transplantation assays

To assess the functional capacity of *Dnmt3a*^W326R/+^ HSCs, BM from 2-month to 3-month-old *Dnmt3a*^+/+^ and *Dnmt3a*^W326R/+^ CD45.2 C57Bl6/JCrl male mice was sorted by FACS, using an LSK-SLAM marker panel (as outlined above in ‘Cell sorting’). Single-cell HSC suspensions (CD45.2) were injected through the tail vein together with ‘whole BM’ helper cells from CD45.1 mice (250 HSCs and 2.5 × 10^5^ BM helper cells per recipient) into 2–4-month-old CD45.1 recipients that had received two split doses of 4.5 Gy irradiation (Cs-137 Ɣ-irradiator). CD45.1 mice were congenic B6.SJL-*Ptprc*^*a*^*Pepc*^*b*^/BoyCrl (Charles River cat nr 494). PB was taken at 1, 2, 3 and 4 months post-transplantation and analyzed for the presence of CD45.2 cells and multilineage reconstitution by flow cytometry (see Flow cytometry section). At 4 months, BM from hind limb bones was similarly analyzed by flow cytometry for CD45.2 HSCs.

To determine the functional capacity of the *Dnmt3a*^W326R/+^ BM niche, reverse transplantation assays were performed using a similar protocol. Briefly, HSCs were FACS sorted from BM of CD45.1 mice (Charles River, 494). Single-cell HSC suspensions (250 CD45.1 HSCs per recipient) were injected through the tail vein of *Dnmt3a*^*+/+*^ or *Dnmt3a*^W326R/+^ CD45.2 C57Bl6/JCrl mice irradiated with a single dose of 4.5 Gy. PB was analyzed at 4 months post-transplantation for the presence of CD45.1 cells and multilineage reconstitution by flow cytometry.

Post-transplantation, donor cell chimerism and multilineage reconstitution were analyzed by flow cytometry using antibody panel comprising CD45.1 APC (1:200; BD Biosciences, 558701), CD45.2 PE (1:200; BD Biosciences, 560695), CD3 PerCP-Cy5.5 (1:100; eBioscience, 45-0031-82), CD11b BV605 (1:100; BioLegend, 101237), CD19 BV650 (1:100; BioLegend, 115541), Gr-1 APC-Cy7 (1:200; BD Biosciences, 557661) and Ter119 BV421 (1:400; BD Biosciences, 563998). Progenitor cell populations in BM were examined with the same LSK-SLAM antibody panel used to sort HSCs for transplant, with the addition of CD45.2 antibody (CD45.2-AF647, 1:200; BioLegend, 109818) to distinguish donor and recipient HSCs. The differentiation quotient for transplanted HSCs was calculated by dividing the percentage of CD45.2 blood chimerism in PB by the percentage of CD45.2 HSCs in BM at 4 months post-transplantation.

### Colony-forming unit-culture assays

Colony-forming unit-culture (CFU-C) assays were performed as described^119^. BM cells were flushed from both femurs and tibias and mechanically dissociated into single cells in PFP. Erythrocytes were lysed with ammonium chloride solution (StemCell Technologies, 07850) for 12 min at room temperature. Cells were counted, plated in duplicate (10,000 BM cells per 35-mm petri dish; Falcon, 1008) in methylcellulose (Methocult GF M3434, StemCell Technologies) supplemented with 1% penicillin–streptomycin, and incubated at 37 °C (5% CO_2_) for 10–12 days. Hematopoietic colonies were counted and distinguished by morphology under an inverted microscope. CFU-C types are defined as—blast forming unit erythroid progenitors, CFU-granulocyte progenitors, macrophage progenitors, granulocyte and macrophage progenitors and granulocyte, erythrocyte, monocyte and megakaryocyte progenitors.

### Intestinal clonogenicity analysis

Clonogenic potential of cells from mouse intestinal crypts was performed as described^120,121^. The most proximal 10 cm of small intestine following the duodenum was isolated from mice, flushed with PBS and cut open longitudinally. Villi were removed by scraping with a microscope coverslip; remaining tissue was cut into ~2 mm^2^ pieces, washed thrice with PBS and incubated in PBSE (PBS + 2 mM EDTA) for 30 min at 4 °C with gentle shaking. PBSE was removed and crypts were isolated from tissue by vigorous pipetting in PBS. Crypts were strained through a 70-μm cell strainer, washed once with PBS and digested with 1 ml of TripLE with 10 μM Y27632 (Tocris) for 20 min at 37 °C with regular dissociation. After digestion into single cells, 1% BSA was added to stop digestion and 10 ml of ‘organoid wash medium’ added—advanced DMEM/F-12 medium (ADF, Gibco) supplemented with 100 U ml^−1^ penicillin, 100 μg ml^−1^ streptomycin, 2 mM L-glutamine, 10 mM HEPES (Life Technologies) and 0.2% primocin solution (Invivogen). Cells were strained through a 40-μm cell strainer, pelleted by centrifugation (500*g*, 5 min) and washed with 10 ml organoid wash medium. Cells were counted using a Countess cell counter (Invitrogen) and 10,000 single cells plated per 10 μl drop of BME (Bio-Techne Cultrex Reduced Growth Factor Basement Membrane Extract, type 2; Pathclear, 3533-010-02) with at least four drops plated for each mouse. Organoid growth medium (500 μl; organoid wash medium supplemented with N2 (Life Technologies, 17502048), B27 (Life Technologies, 17504044), 50 ng ml^−1^ EGF (Peprotech, 315-09-500), 2% Noggin conditioned medium, 15% R-spondin-1 conditioned medium and 10 μM Y27632 (Tocris)) was added per well. Spheres/clones were counted manually after 4 days, blindly to genotype. Clonogenic capacity was determined by calculating the percentage of spheres with an observable lumen formed per single cell plated.

### Intestinal regeneration after oxaliplatin treatment

To assess intestinal regeneration, mice were given a single intraperitoneal injection of oxaliplatin (Bio-Techne, 2623) in sterile PBS, at 10 mg kg^−1^. Mice were killed 72 h after injection, and 2 h before tissue collection, mice were given 200 μl Cell Proliferation Labeling Reagent (GE Healthcare, RPN201) by intraperitoneal injection. The most proximal 10 cm of the small intestine following the duodenum was collected and divided into ten equally sized pieces. These were then arranged on surgical tape and fixed in 4% paraformaldehyde in PBS for 24 h at 4 °C, and transferred to 70% ethanol before further processing. Standard immunohistochemistry techniques were used, and proliferating cells were labeled with anti-BrdU antibody (1:200; BD Biosciences, 347580). Images were digitized using a NanoZoomer Digital slide scanner and NDP.view2 software (Hamamatsu). Automated scoring of sections was performed using QuPath^116^ (https://qupath.github.io/) with the operator blinded to genotypes. Regenerative crypts were scored as those containing six or more consecutive BrdU-positive epithelial cells. Average percentage of regenerative crypts was calculated by dividing the number of regenerative crypts by the total number of crypts per cross-section across ten gut pieces.

### Statistical analysis

Statistical testing was performed using R (v4.4.1) and GraphPad Prism 10. Tests used are indicated in all figure legends. All tests were two sided unless otherwise stated. Details for specific analyses are provided in relevant subsections in Methods, as described above.

### Reporting summary

Further information on research design is available in the Nature Portfolio Reporting Summary linked to this article.

## Extended Data

## Supplementary Material

**Supplementary information** The online version contains supplementary material available at https://doi.org/10.1038/s41588-026-02633-8.

Supplementary

Supplementary Material

## Data Availability

Processed and sequencing data generated as part of this study are available in GEO under accessions—GSE324236 (https://www.ncbi.nlm.nih.gov/geo/query/acc.cgi?acc=GSE324236) for Infinium methylation arrays, GSE324227 (https://www.ncbi.nlm.nih.gov/geo/query/acc.cgi?acc=GSE324227) for EM-seq, GSE324229 (https://www.ncbi.nlm.nih.gov/geo/query/acc.cgi?acc=GSE324229) for VDJ-seq and GSE319569 (https://www.ncbi.nlm.nih.gov/geo/query/acc.cgi?acc=GSE319569) for scRNA-seq. Previously published datasets used in this study are as follows: mm10 (GSM8027450, GSM8027453, GSM8027451, GSM8027448) and hg19 (GSE31263, GSM772868, GSM997231, GSM997240, GSM772867), GS^111^. For GS data access requests, see ‘GS Access Request Form’ at https://www.ed.ac.uk/generation-scotland/for-researchers/access. Requests require approval by the Generation Scotland Access Committee to ensure compliance with participant consent. Requests for materials should be addressed to A.P.J. Source data are provided with this paper.

## Code availability

Code documented in methods is available on GitHub at https://github.com/Gottgens-lab/Jackson_Lab_scRNAseq and https://github.com/dansarni/DNMT3A-HESJAS-ageing (https://doi.org/10.5281/zenodo.19692239).

## References

[1] Lopez-Otin C, Blasco MA, Partridge L, Serrano M, Kroemer G (2013). The hallmarks of aging. Cell.

[2] Lopez-Otin C, Blasco MA, Partridge L, Serrano M, Kroemer G (2023). Hallmarks of aging: an expanding universe. Cell.

[3] Petkovich DA (2017). Using DNA methylation profiling to evaluate biological age and longevity interventions. Cell Metab.

[4] Wang T (2017). Epigenetic aging signatures in mice livers are slowed by dwarfism, calorie restriction and rapamycin treatment. Genome Biol.

[5] Lu AT (2023). Universal DNA methylation age across mammalian tissues. Nat Aging.

[6] Zhang Q (2019). Improved precision of epigenetic clock estimates across tissues and its implication for biological ageing. Genome Med.

[7] De Magalhaes JP (2024). Distinguishing between driver and passenger mechanisms of aging. Nat Genet.

[8] Cook D (2014). Lessons learned from the fate of AstraZeneca’s drug pipeline: a five-dimensional framework. Nat Rev Drug Discov.

[9] Moqri M (2024). PRC2-AgeIndex as a universal biomarker of aging and rejuvenation. Nat Commun.

[10] Rakyan VK (2010). Human aging-associated DNA hypermethylation occurs preferentially at bivalent chromatin domains. Genome Res.

[11] Teschendorff AE (2010). Age-dependent DNA methylation of genes that are suppressed in stem cells is a hallmark of cancer. Genome Res.

[12] Boulard M, Edwards JR, Bestor TH (2015). FBXL10 protects Polycomb-bound genes from hypermethylation. Nat Genet.

[13] Gu T (2018). DNMT3A and TET1 cooperate to regulate promoter epigenetic landscapes in mouse embryonic stem cells. Genome Biol.

[14] Dixon G (2021). QSER1 protects DNA methylation valleys from de novo methylation. Science.

[15] Heyn P (2019). Gain-of-function DNMT3A mutations cause microcephalic dwarfism and hypermethylation of Polycomb-regulated regions. Nat Genet.

[16] Online Mendelian Inheritance in Man (2024). OMIM entry number.

[17] Sendzikaite G, Hanna CW, Stewart-Morgan KR, Ivanova E, Kelsey G (2019). A DNMT3A PWWP mutation leads to methylation of bivalent chromatin and growth retardation in mice. Nat Commun.

[18] Kibe K (2021). The DNMT3A PWWP domain is essential for the normal DNA methylation landscape in mouse somatic cells and oocytes. PLoS Genet.

[19] Xie W (2013). Epigenomic analysis of multilineage differentiation of human embryonic stem cells. Cell.

[20] Li Y (2018). Genome-wide analyses reveal a role of Polycomb in promoting hypomethylation of DNA methylation valleys. Genome Biol.

[21] Weinberg DN (2021). Two competing mechanisms of DNMT3A recruitment regulate the dynamics of de novo DNA methylation at PRC1-targeted CpG islands. Nat Genet.

[22] Gu T (2022). The disordered N-terminal domain of DNMT3A recognizes H2AK119ub and is required for postnatal development. Nat Genet.

[23] Chen X (2024). Structural basis for the H2AK119ub1-specific DNMT3A-nucleosome interaction. Nat Commun.

[24] Wapenaar H (2024). The N-terminal region of DNMT3A engages the nucleosome surface to aid chromatin recruitment. EMBO Rep.

[25] Gretarsson KH (2024). Cancer-associated DNA hypermethylation of Polycomb targets requires DNMT3A dual recognition of histone H2AK119 ubiquitination and the nucleosome acidic patch. Sci Adv.

[26] Futagawa H, Ito S, Kosaki K, Yoshihashi H (2023). Long-term clinical course of Heyn–Sproul–Jackson syndrome. Congenit Anom.

[27] Kim GH, Kim J, Lee J, Jang DH (2023). A novel pathogenic variant of *DNMT3A* associated with craniosynostosis: a case report of Heyn–Sproul–Jackson syndrome. Front Pediatr.

[28] Mellid S (2020). Novel *DNMT3A* germline variant in a patient with multiple paragangliomas and papillary thyroid carcinoma. Cancers.

[29] Remacha L (2018). Gain-of-function mutations in DNMT3A in patients with paraganglioma. Genet Med.

[30] Adam MP (1993). GeneReviews((R))(Bookshelf ID: NBK1121).

[31] Adam MP (1993). GeneReviews((R)) (Bookshelf ID: NBK1514).

[32] Wilson BT (2016). The Cockayne Syndrome Natural History (CoSyNH) study: clinical findings in 102 individuals and recommendations for care. Genet Med.

[33] Kunstyr I, Leuenberger HG (1975). Gerontological data of C57BL/6J mice. I. Sex differences in survival curves. J Gerontol.

[34] Lee S (2008). Comparison between surrogate indexes of insulin sensitivity and resistance and hyperinsulinemic euglycemic clamp estimates in mice. Am J Physiol Endocrinol Metab.

[35] Gray SL (2006). Leptin deficiency unmasks the deleterious effects of impaired peroxisome proliferator-activated receptor γ function (P465L PPARgamma) in mice. Diabetes.

[36] Challen GA (2011). Dnmt3a is essential for hematopoietic stem cell differentiation. Nat Genet.

[37] Wu H (2010). Dnmt3a-dependent nonpromoter DNA methylation facilitates transcription of neurogenic genes. Science.

[38] Jeong M (2018). Loss of Dnmt3a immortalizes hematopoietic stem cells in vivo. Cell Rep.

[39] Sato T (2009). Single Lgr5 stem cells build crypt-villus structures in vitro without a mesenchymal niche. Nature.

[40] Van der Velde A (2021). Annotation of chromatin states in 66 complete mouse epigenomes during development. Commun Biol.

[41] Zhou W (2018). DNA methylation loss in late-replicating domains is linked to mitotic cell division. Nat Genet.

[42] Voigt P, Tee WW, Reinberg D (2013). A double take on bivalent promoters. Genes Dev.

[43] Macrae TA, Fothergill-Robinson J, Ramalho-Santos M (2023). Regulation, functions and transmission of bivalent chromatin during mammalian development. Nat Rev Mol Cell Biol.

[44] Blackledge NP, Klose RJ (2021). The molecular principles of gene regulation by Polycomb repressive complexes. Nat Rev Mol Cell Biol.

[45] Reddington JP, Sproul D, Meehan RR (2014). DNA methylation reprogramming in cancer: does it act by re-configuring the binding landscape of Polycomb repressive complexes?. Bioessays.

[46] Buske C (2002). Deregulated expression of HOXB4 enhances the primitive growth activity of human hematopoietic cells. Blood.

[47] Calvanese V (2019). MLLT3 governs human haematopoietic stem-cell self-renewal and engraftment. Nature.

[48] Christodoulou C (2020). Live-animal imaging of native haematopoietic stem and progenitor cells. Nature.

[49] Cobaleda C, Schebesta A, Delogu A, Busslinger M (2007). Pax5: the guardian of B cell identity and function. Nat Immunol.

[50] Feinberg MW (2007). The Kruppel-like factor KLF4 is a critical regulator of monocyte differentiation. EMBO J.

[51] Frelin C (2013). GATA-3 regulates the self-renewal of long-term hematopoietic stem cells. Nat Immunol.

[52] Hock H (2003). Intrinsic requirement for zinc finger transcription factor Gfi-1 in neutrophil differentiation. Immunity.

[53] Laslo P (2006). Multilineage transcriptional priming and determination of alternate hematopoietic cell fates. Cell.

[54] Lawrence HJ (2005). Loss of expression of the Hoxa-9 homeobox gene impairs the proliferation and repopulating ability of hematopoietic stem cells. Blood.

[55] Lawrence SM, Corriden R, Nizet V (2018). The ontogeny of a neutrophil: mechanisms of granulopoiesis and homeostasis. Microbiol Mol Biol Rev.

[56] Lehnertz B (2021). HLF expression defines the human hematopoietic stem cell state. Blood.

[57] Lin YC (2010). A global network of transcription factors, involving E2A, EBF1 and Foxo1, that orchestrates B cell fate. Nat Immunol.

[58] Sauvageau G (1995). Overexpression of HOXB4 in hematopoietic cells causes the selective expansion of more primitive populations in vitro and in vivo. Genes Dev.

[59] Schonheit J (2013). PU.1 level-directed chromatin structure remodeling at the Irf8 gene drives dendritic cell commitment. Cell Rep.

[60] Weber BN (2011). A critical role for TCF-1 in T-lineage specification and differentiation. Nature.

[61] Xiang P (2017). A knock-in mouse strain facilitates dynamic tracking and enrichment of MEIS1. Blood Adv.

[62] Yui MA, Rothenberg EV (2014). Developmental gene networks: a triathlon on the course to T cell identity. Nat Rev Immunol.

[63] Medvedovic J, Ebert A, Tagoh H, Busslinger M (2011). Pax5: a master regulator of B cell development and leukemogenesis. Adv Immunol.

[64] Fuxa M (2004). Pax5 induces V-to-DJ rearrangements and locus contraction of the immunoglobulin heavy-chain gene. Genes Dev.

[65] Hill L (2020). Wapl repression by Pax5 promotes V gene recombination by Igh loop extrusion. Nature.

[66] Heyn H (2012). Distinct DNA methylomes of newborns and centenarians. Proc Natl Acad Sci USA.

[67] Horvath S (2013). DNA methylation age of human tissues and cell types. Genome Biol.

[68] Lu AT (2019). DNA methylation GrimAge strongly predicts lifespan and healthspan. Aging.

[69] Levine ME (2018). An epigenetic biomarker of aging for lifespan and healthspan. Aging.

[70] Hannum G (2013). Genome-wide methylation profiles reveal quantitative views of human aging rates. Mol Cell.

[71] Meer MV, Podolskiy DI, Tyshkovskiy A, Gladyshev VN (2018). A whole lifespan mouse multi-tissue DNA methylation clock. eLife.

[72] Weeda G (1997). Disruption of mouse ERCC1 results in a novel repair syndrome with growth failure, nuclear abnormalities and senescence. Curr Biol.

[73] Mounkes LC, Kozlov S, Hernandez L, Sullivan T, Stewart CL (2003). A progeroid syndrome in mice is caused by defects in A-type lamins. Nature.

[74] Di Pino A, DeFronzo RA (2019). Insulin resistance and atherosclerosis: implications for insulin-sensitizing agents. Endocr Rev.

[75] Beerman I, Maloney WJ, Weissmann IL, Rossi DJ (2010). Stem cells and the aging hematopoietic system. Curr Opin Immunol.

[76] Compston JE, McClung MR, Leslie WD (2019). Osteoporosis. Lancet.

[77] Crofts SJC, Latorre-Crespo E, Chandra T (2024). DNA methylation rates scale with maximum lifespan across mammals. Nat Aging.

[78] Sollis E (2023). The NHGRI-EBI GWAS Catalog: knowledgebase and deposition resource. Nucleic Acids Res.

[79] Beerman I (2013). Proliferation-dependent alterations of the DNA methylation landscape underlie hematopoietic stem cell aging. Cell Stem Cell.

[80] Sun D (2014). Epigenomic profiling of young and aged HSCs reveals concerted changes during aging that reinforce self-renewal. Cell Stem Cell.

[81] Iwama A (2004). Enhanced self-renewal of hematopoietic stem cells mediated by the polycomb gene product Bmi-1. Immunity.

[82] Xie H (2014). Polycomb repressive complex 2 regulates normal hematopoietic stem cell function in a developmental-stage-specific manner. Cell Stem Cell.

[83] Lee SC (2015). Polycomb repressive complex 2 component Suz12 is required for hematopoietic stem cell function and lymphopoiesis. Blood.

[84] Xavier Raj I (2025). Non-canonical functions of DNMT3A in hematopoietic stem cells regulate telomerase activity and genome integrity. Cell Stem Cell.

[85] Hardy RR, Carmack CE, Shinton SA, Kemp JD, Hayakawa K (1991). Resolution and characterization of pro-B and pre-pro-B cell stages in normal mouse bone marrow. J Exp Med.

[86] Iritani BM, Forbush KA, Farrar MA, Perlmutter RM (1997). Control of B cell development by Ras-mediated activation of Raf. EMBO J.

[87] Triche TJ, Weisenberger DJ, Van Den Berg D, Laird PW, Siegmund KD (2013). Low-level processing of Illumina Infinium DNA methylation BeadArrays. Nucleic Acids Res.

[88] Fortin JP, Triche TJ, Hansen KD (2017). Preprocessing, normalization and integration of the Illumina HumanMethylationEPIC array with minfi. Bioinformatics.

[89] Quinlan AR, Hall IM (2010). BEDTools: a flexible suite of utilities for comparing genomic features. Bioinformatics.

[90] Wu H, Wang C, Wu Z (2013). A new shrinkage estimator for dispersion improves differential expression detection in RNA-seq data. Biostatistics.

[91] Feng H, Conneely KN, Wu H (2014). A Bayesian hierarchical model to detect differentially methylated loci from single nucleotide resolution sequencing data. Nucleic Acids Res.

[92] Wu H (2015). Detection of differentially methylated regions from whole-genome bisulfite sequencing data without replicates. Nucleic Acids Res.

[93] Park Y, Wu H (2016). Differential methylation analysis for BS-seq data under general experimental design. Bioinformatics.

[94] Zhu LJ (2010). ChIPpeakAnno: a Bioconductor package to annotate ChIP–seq and ChIP–chip data. BMC Bioinformatics.

[95] Yu G (2010). GOSemSim: an R package for measuring semantic similarity among GO terms and gene products. Bioinformatics.

[96] Yu G, Wang LG, Han Y, He QY (2012). clusterProfiler: an R package for comparing biological themes among gene clusters. OMICS.

[97] Ernst J, Kellis M (2012). ChromHMM: automating chromatin-state discovery and characterization. Nat Methods.

[98] Ramirez F (2016). deepTools2: a next generation web server for deep-sequencing data analysis. Nucleic Acids Res.

[99] Mina AI (2018). CalR: a web-based analysis tool for indirect calorimetry experiments. Cell Metab.

[100] Pennington ZT (2019). ezTrack: an open-source video analysis pipeline for the investigation of animal behavior. Sci Rep.

[101] Ramachandran P (2019). Resolving the fibrotic niche of human liver cirrhosis at single-cell level. Nature.

[102] Lun ATL (2019). EmptyDrops: distinguishing cells from empty droplets in droplet-based single-cell RNA sequencing data. Genome Biol.

[103] Wolf FA, Angerer P, Theis FJ (2018). SCANPY: large-scale single-cell gene expression data analysis. Genome Biol.

[104] Wolock SL, Lopez R, Klein AM (2019). Scrublet: computational identification of cell doublets in single-cell transcriptomic data. Cell Syst.

[105] Isobe T (2023). Preleukemic single-cell landscapes reveal mutation-specific mechanisms and gene programs predictive of AML patient outcomes. Cell Genom.

[106] Burkhardt DB (2021). Quantifying the effect of experimental perturbations at single-cell resolution. Nat Biotechnol.

[107] Lange M (2022). CellRank for directed single-cell fate mapping. Nat Methods.

[108] Virtanen P (2020). SciPy 1.0: fundamental algorithms for scientific computing in Python. Nat Methods.

[109] Sanchez-Luque FJ (2019). LINE-1 evasion of epigenetic repression in humans. Mol Cell.

[110] Chovanec P (2018). Unbiased quantification of immunoglobulin diversity at the DNA level with VDJ-seq. Nat Protoc.

[111] Walker RM (2025). Data Resource Profile: Whole-Blood DNA Methylation Resource in Generation Scotland (MeGS). Int J Epidemiol.

[112] Pidsley R (2016). Critical evaluation of the Illumina MethylationEPIC BeadChip microarray for whole-genome DNA methylation profiling. Genome Biol.

[113] Pelegi-Siso D, de Prado P, Ronkainen J, Bustamante M, Gonzalez JR (2021). methylclock: a Bioconductor package to estimate DNA methylation age. Bioinformatics.

[114] Feldman AT, Wolfe D (2014). Tissue processing and hematoxylin and eosin staining. Methods Mol Biol.

[115] Van ‘t Hof RJ (2012). Analysis of bone architecture in rodents using microcomputed tomography. Methods Mol Biol.

[116] Bankhead P (2017). QuPath: open source software for digital pathology image analysis. Sci Rep.

[117] Glasson SS, Chambers MG, Van Den Berg WB, Little CB (2010). The OARSI histopathology initiative—recommendations for histological assessments of osteoarthritis in the mouse. Osteoarthritis Cartilage.

[118] Sarkis R (2023). MarrowQuant 2.0: a digital pathology workflow assisting bone marrow evaluation in experimental and clinical hematology. Mod Pathol.

[119] Sa da Bandeira D (2022). PDGFRβ^+^ cells play a dual role as hematopoietic precursors and niche cells during mouse ontogeny. Cell Rep.

[120] Hall AE (2022). RNA splicing is a key mediator of tumour cell plasticity and a therapeutic vulnerability in colorectal cancer. Nat Commun.

[121] Gudino V (2021). RAC1B modulates intestinal tumourigenesis via modulation of WNT and EGFR signalling pathways. Nat Commun.

[122] Forget P (2017). What is the normal value of the neutrophil-to-lymphocyte ratio?. BMC Res Notes.

[123] Huang YH (2017). DNA epigenome editing using CRISPR–Cas SunTag-directed DNMT3A. Genome Biol.

