# Supplementary material for: A progeria syndrome links DNA hypermethylation to age-related pathology
Source: Nat Genet. Author manuscript; Available in PMC 2026 Jul 24. (PMC13364717; doi:10.1038/s41588-026-02633-8)
Supplement: Supplementary [file EMS215958-supplement-Supplementary.pdf]

Supplementary Information – Table of Contents

Supplementary Tables

Supplementary Table 1. Variants identified in DNMT3A.

Supplementary Table 2. a, Clinical phenotype of DNMT3A subjects (P1-P6).

Supplementary Table 2. b, Clinical phenotype of DNMT3A subjects (P7-P13).

Supplementary Table 3. Micro-CT measurements mouse femur trabecular bone.

Supplementary Table 4. Micro-CT measurements mouse femur cortical bone.

Supplementary Table 5. 3-point bending test measurements for mouse femurs.

Supplementary Figures

Supplementary Figure 1. Representative gating strategy for Fig. 3b.

Supplementary Figure 2. Representative gating strategy for Fig. 3d-g.

Supplementary Figure 3. Representative gating strategy for Fig. 5c-d.

Supplementary Table 1. Variants identified in DNMT3A.

Individual	Nucleotide Alterations	Predicted Amino Acid Consequence	Sex	Country of origin
P1 in Heyn 2019	c.988T>C	p.W330R	F	USA
P2 in Heyn 2019	c.988T>C	p.W330R	M	New Zealand
P3 in Heyn 2019	c.997G>A	p.D333N	M	Spain
P4	c.911_913del	p.S304del	F	France
P5	c.911_913del	p.S304del	F	USA
P6	c.998A>T	p.D333V	F	USA
P7	c.989G>C	p.W330S	M	Norway
P8	c.916T>C	p.W306R	M	France
P9	c.1003A>G	p.K335E	M	Spain
P10	c.911_913del	p.S304del	F	Mexico
P11	c.899T>G	p.L300R	M	USA
P12	c.994G>A	p.G332R	F	France
P13	c.989G>T	p.W330L	F	Spain

Supplementary Table 2. a, Clinical phenotype of DNMT3A subjects (P1-P6).

Patient	P1	P2	P3	P4	P5	P6
**Age**	23y (deceased)	12y	8y	10y	15y	8y
**Growth restriction**	Pre+postnatal	Pre+postnatal	Pre+postnatal	Postnatal	Pre+postnatal	Pre+postnatal
**Hair**	Sparse, fine hair	Sparse, fine hair	Fine, thin, blond, sparse hair	Sparse hair	Fine, thin, blond, sparse hair	Very thin, sparse, slow growing hair; high posterior hairline
**Haematology**	Chronic anemia and leukopenia. Bone marrow (BM) biopsy: hypocellular for age with concomitant pancytopenia; mild dyspoeitic changes only. BM FACS: 83% myeloid, 9% monocytes, 6% lymphocytes, 2% myeloid blasts, <5% CD34 blasts	Not available	Lymphopenia (0.9x 1000/mm3 NR 1.5-5). B cells 8% (low); CD4 22% (low); CD8 7% (low); NK cells 56% (high). IgG, A,M normal (G1,G2,G3,G4 also normal)	B and T lymphopenia; CD8, CD19 low; CD4, NK normal. Mild normocytic anemia (Hb11)	Normal CBC	lymphopenia
**Infections/ Immunology**	Otitis media. Frequent episodes suspected sepsis age 20y. Dental abscess. Left lower lobe pneumonia. IgG,A,M normal. Died presumed sepsis episode age 23y.	Recurrent upper respiratory tract infections. Chronic otitis media	Recurrent respiratory tract infections; bronchitis; chronic otitis media. Haemophilus influenzae and E.coli. Primary immunodeficiency. Trimethoprim and sulfametoxazol antibiotic prophylaxis	Recurrent cutaneous infections (abscesses), URTI, rhinitis and gastroenteritis. Normal IgG,A,M levels. Normal vaccine responses: IgG, tetanus, pneumococcus, rubella	RSV at 6m; Rotavirus at 9m; breathing problems at 1.5y; tonsillectomy and adenoidectomy at 5y; pneumonia at 7y; UTI at 12y; frequent hospital visits for low grade fever and breathing problems	Frequent infections (ear and UTI); gastritis; history of low lymphocytes
**Skeletal**	Osteopenia. Fracture proximal right femur. Proportionate short stature; 11 pairs of ribs; bone age of 3m at age 12m; Multiple areas of fibrous dysplasia right femur; ganglion cyst, wrist	Bone age of 15m at 19m; skeletal survey: short broad metacarpals and phalanges; multiple lytic/sclerotic lesions consistent with fibrous dysplasia; 3 pathologic fractures associated with these lesions in humeri and femur	Proportional short stature; bone age of 5y at 4.5y; short broad metacarpals and phalanges	Fragile bones with multiple fractures. 5th finger clinodactyly x2; overlapping II-III toes bilateral	Borderline accelerated bone age; skeletal survey: longitudinally-oriented striations in the metaphyses, gracile long bones with thin diaphyses, tall appearance of vertebral bodies with depression of posterior aspect of endplates, especially in inferior endplates and thoracolumbar region.	Short stature; normal bone age at 2.5y
**Metabolic/ Adipose (adults)**						
**Skin**		Pruritis		Dry skin	Eczema	Skin diffusely xerotic; ichthyosis vulgaris; dry scaly skin; hypopigmented

						lesions; abnormal nail structure
**Facial appearance**			Wide forehead; hypertelorism	Bulbous nose; thin lips	Mild frontal bossing; thin lips; wide mouth; long philtrum	Plagiocephaly; micrognathia; wide mouth; abnormally shaped teeth; broad nasal tip
**ENT**	Simple ears without a prominent antitragus	Hearing loss	Small ears; recurrent otitis resulting in conductive hearing loss	Low set and protruding ears	Low set, small, normally rotated ears; PE tubes placed at 12y	Low set, rotated ears; simplified external ears
**Eye**	Bilateral cataracts during childhood		Strabismus; hypertelorism; epicanthic folds	Strabismus astigmatism; severe progressive visual deficiency with optic nerve damage	Hypometropia strabismus	Mild hyperopic astigmatism
**Developmental delay**	Severe	Moderate	Moderate-severe	Moderate	Severe	Severe
**Development**	Global developmental delay; combined speech and motor; attained 18m level by 23y	Global developmental delay; walking at 3y; few words at 5y	Severe psychomotor delay; delayed speech	Global developmental delay; walked at 3y; first sentence at 5y	Global developmental delay; severe speech delay; babbling at 12y with few words; walked at 2y; cognitive age of 12-18m at 12y; severe behavioural difficulties; improving with fluoxetine treatment	Global developmental delay; walked at 2.5y; sentences at 4y; behavioural issues
**CNS**	Microcephaly; grand mal seizures; MRI: 'cerebral atrophy'	Microcephaly; MRI: structurally normal brain	Microcephaly; MRI: structurally normal brain	Microcephaly; normal MRI	Lower limb hyperreflexia. MRI at 8y: choroid plexus cysts, glomus of right and left choroid plexus; normal MR spectroscopy	Hypotonia, balance and coordination issues; microcephaly; plagiocephaly; epilepsy; MRI structurally normal brain; normal pituitary
**Cardiac**				Normal cardiac ultrasound.	No	Atrial septal defect; palpitations
**Family history**	No	No	Maternal great grandmother & paternal uncle <150cm height.	No	No	No
**Consanguineou**s	No	No	No	No	No	No
**Other**	Gastritis	Episodic diarrhoea; difficult to maintain weight. Normal microarray CGH and metabolic screen; normal IGF1 and GH.	Bilateral macro-orchidism; Normal IGF1, high IGFBP-3. Aggressive behaviour from age 3-4y; on antipsychotics and GnRH to suppress precocious puberty (age 6.5y).	GH treatment between age 4-6y (no effect); no GH deficiency; no thyroid deficiency; dental malposition; unexplained polyuria	Gastrostomy from 3y to 11y; poor eater; constant diarrhoea - resolved after changing formula at age 15y; late onset puberty	Chronic constipation; underweight; gastritis; fatigue; always cold; GH deficiency; GH therapy from 4y; pituitary dwarfism

Supplementary Table 2. b, Clinical phenotype of DNMT3A subjects (P7-P13).

Patient	P7	P8	P9	P10	P11	P12	P13
**Age**	5y	12y	31y	10y 9m	7y (deceased)	42y	63y
**Growth restriction**	Pre+postnatal	Pre+postnatal	Pre+postnatal	Pre+postnatal	Pre+postnatal	None	Short stature
**Hair**	Sparse, fine hair	Thin, sparse hair.	Sparse, fine hair.	Very thin, sparse, slow growing hair	Thin, sparse hair	Fine hair	Sparse, very fine hair; high posterior hairline
**Haematology**	Not available	Normal CBC	Not available	Lymphopenia, particularly B cell deficiency; decreased mean platelet volume	T-B-NK+ SCID requiring bone marrow transplant; severe neutropenia pre-transplant; history of progressing profound lymphopenia. BM biopsy: no infiltrate or malignancy; evidence of iron deficiency anaemia.	Not available	Normochromic normocytic anaemia
**Infections/ Immunology**	No more infections than usual	Not available	Not available	Recurrent otitis media; inadequate vaccine response to polysaccharides; normal immunoglobulin levels; low levels of memory B lymphocytes; normal isotype switched and non-isotype switched B lymphocytes	Adequate number of normalized TREC; appropriate thymic and T cell function	No	Not available
**Skeletal**	Fracture of right humerus at age 4y 9m without any known preceding trauma; bone density not measured. Healed without problems/complications. Skeletal survey not done.	Osteopenia; multiple fractures of long bones	Osteoporosis; multiple fractures; elongated fingers; thickening in interphalangeal joints; narrow chest with low-set nipples	Proportionate short stature; skeletal survey: scoliosis, small sella turcica, malar hypoplasia, long-lined chest, genu-valgus, slight hunching of tibias, premature closure of growth discs, ulnar shortening, tall vertebral bodies; advanced bone age	Severe osteopenia requiring bisphosphonate infusions; generalized low bone density; multiple fractures; bone biopsy showed lamellar bone fragments, focal fibrosis. Died of metastatic osteosarcoma (left femur) age 7y	Mild scoliosis	Short stature; short metacarpal phalanges; tapering fingers with few creases
**Metabolic/ Adipose (adults)**	Lipodystrophic appearance reported by clinician.	Lipodystrophic appearance reported.	Lipodystrophic appearance reported; loss of subcutaneous fat in limbs; increased abdominal adiposity as adult; no diabetes; acanthosis nigricans.			Not assessed	Lipodystrophic appearance reported. Reduced subcutaneous fat in limbs and abdominal obesity. Acanthosis nigricans. Insulin not assessed. Polycystic ovary syndrome age 15y. ALT elevated, 58U/l (5-34), AST 37 (5-27). Low HDL cholesterol (54mg/dL (>=65). Normal TGs. No

							data insulin/leptin, adiponectin
**Skin**	Normal	Hyperkeratosis	Very soft skin	Dry skin	Dry skin		Longitudinal striae on nails
**Facial appearance**	Downslant; mild frontal bossing; thin upper lip; bulbous nose tip		Small, round face; wide nasal tip; anteverted nostrils; high palate	Bulbous nose; thin lips	Facial dysmorphism	Facial dysmorphism	Small, round face; wide forehead; thin upper lip; bulbous nose; anteverted nostrils; long nasolabial furrow
**ENT**	Small, posteriorly rotated, simple ears	Normal ears	Normal ears	Posteriorly rotated ears	Sensorineural hearing loss	Normal ears	Low set, posteriorly rotated ears
**Eye**	Deep-set eyes; no strabismus		Deep-set eyes	Deep-set eyes; bilateral myopia -6.5D; Fundus: macrodisc, peripapillary atrophy, vascular attenuation, possible macular changes			Wide, deep-set eyes
**Developmental delay**	Mild/moderate	Severe	Mild	Severe	Severe	Mild	Moderate
**Development**	Developmental delay; walked at 2y, speaks a few single words at 3y; hyperactive.<br>Rigid behaviour, tendency to destroy items, poor sleep at night, but also very cheerful and smiling.	Developmental delay; first words at 3y; very hyperactive; agitation and stereotypic behaviours	Developmental delay	Developmental delay; first words at 3y; first sentence at 5y; walked at 2.5y; behavioural difficulties improving with risperidone.	Global developmental delay		
**CNS**	Microcephaly; normal cerebral MRI; no seizures. Swallowing difficulties (dysphagia), also when drinking	Microcephaly; plagiocephaly; normal MRI	Microcephaly; Arnold-Chiari type I malformation	Microcephaly; MRI with ventricular asymmetry	Microcephaly		Plagiocephaly; seizures; hyporeflexia; hypotonia; altered balance
**Cardiac**		Fallot's tetralogy; TGV; CIV		Normal cardiac ultrasound	Normal cardiac ultrasound	No	
**Family history**	No	No	No	No	No	No	
**Consanguineous**	No	No	No	No	No	No	
**Other**	Major feeding problems; PEG gastric bypass; does not tolerate large amounts of food (vomits); frequent diarrhoea; major sleeping problems	Bilateral cryptorchidism; dental abnormalities; hyperhidrosis	2 parotid paragangliomas; 2 cervical paragangliomas; GH treatment; delayed secondary dentition; transparency of skin - increased visible blood vessels; aged appearance	Major sleeping problems; menarche at 10y; levels of LH, estradiol and FSH corresponding in pubertal ranges; chronic diarrhoea	Gastric tube dependence; precocious puberty, central hypothyroidism; chronic kidney disease; small kidneys	Bilateral carotid body paragangliomas	Multiple head and neck paragangliomas; lipodystrophy; delayed puberty; polycystic ovarian cyst. Sella turcica tumour - radiotherapy

Supplementary Table 3. Micro-CT measurements mouse femur trabecular bone.

	Male						Female					
	+/+		W326R/+		Mutant vs WT		+/+		W326R/+		Mutant vs WT	
	n = 5		*n* = 4				*n* = 5		*n* = 5			
	Mean	s.d.	Mean	s.d.	% change	*P*-value	Mean	s.d.	Mean	s.d.	% change	*P*-value
**Tissue volume (mm^3)**	2.87	0.113	1.67	0.0873	-41.7	5.19×10^{-7}	2.14	0.129	1.57	0.0597	-26.6	1.88×10^{-5}
**Bone volume (mm^3)**	0.694	0.208	0.198	0.0363	-71.4	0.0023	0.145	0.0182	0.0172	0.0112	-88.2	9.29×10^{-7}
**Percentage bone volume (%)**	24.0	6.6	11.8	2.1	-50.8	0.010	6.75	0.65	1.10	0.75	-83.7	1.34×10^{-6}
**Trabecular thickness (µm)**	67.5	5.9	71.2	0.83	5.4	0.26	57.7	5.6	55.3	3.0	-4.0	0.43
**Trabecular separation (µm)**	190	26.4	292	14.2	53.7	0.0002	313	24.5	651	114	108	1.89×10^{-4}
**Trabecular number (1/mm)**	3.53	0.86	1.66	0.29	-53.0	0.0045	1.18	0.16	0.20	0.13	-83.4	4.44×10^{-6}
**Trabecular pattern factor (1/µm)**	0.012	0.005	0.019	0.002	59.6	0.031	0.029	0.003	0.037	0.004	26.8	0.0055
**Structure model index**	1.34	0.40	2.13	0.18	58.3	0.0093	2.44	0.12	2.83	0.12	16.1	0.0009
**Connectivity density ($1/mm^3$)**	201	44.3	77.9	28.9	-61.3	0.0020	81.7	18.2	16.6	5.55	-79.6	6.00×10^{-5}
**Density (mg/cm^3)**	912	14.6	995	9.7	9.0	2.74×10^{-5}	879	47.7	887	31.4	0.90	0.77

Supplementary Table 4. Micro-CT measurements mouse femur cortical bone.

	Male						Female					
	+/+		W326R/+		Mutant vs WT		+/+		W326R/+		Mutant vs WT	
	n = 5		*n* = 4				*n* = 5		*n* = 5			
	Mean	s.d.	Mean	s.d.	% change	*P* value	Mean	s.d.	Mean	s.d.	% change	*P* value
Tissue volume (mm^3)	1.22	0.0819	0.598	0.0322	-51.1	2.00×10^{-6}	0.853	0.0234	0.547	0.00317	-35.8	1.25×10^{-7}
Bone volume (mm^3)	0.529	0.0425	0.331	0.00729	-37.5	3.97×10^{-5}	0.446	0.0222	0.343	0.0169	-23.1	3.45×10^{-5}
Cortical thickness (µm)	209	8.0	217	0.95	3.5	0.11	231	11.6	250	13.2	8.1	0.044
Moment of inertia (x) (mm^5)	0.112	0.0139	0.0368	0.00301	-67.3	1.48×10^{-5}	0.0704	0.00454	0.0368	0.00312	-47.7	8.04×10^{-7}
Moment of inertia (y) (mm^5)	0.268	0.0437	0.0933	0.00860	-65.1	0.0001	0.142	0.0122	0.0749	0.00904	-47.3	8.96×10^{-7}
Moment of inertia (z) (mm^5)	0.358	0.0539	0.120	0.0112	-66.5	0.0001	0.194	0.0148	0.101	0.0111	-48.1	3.42×10^{-6}
Polar moment of inertia (mm^5)	0.369	0.0548	0.120	0.0113	-66.1	0.0001	0.203	0.0152	0.106	0.0114	-47.8	3.08×10^{-6}
Tissue mineral density (mg HA/cm^3)	1,357	20	1,436	11	5.8	0.0002	1,431	19	1,492	30	4.26	0.0051
Mean total periosteal perimeter (µm)	6,297	268	4,751	144	-24.5	1.76×10^{-5}	5,105	101	4356	134	-14.7	4.23×10^{-6}
Mean total endosteal perimeter (µm)	5,162	368	3,282	159	-36.4	3.15×10^{-5}	3,602	67	2710	144	-24.8	1.49×10^{-6}

Supplementary Table 5. 3-point bending test measurements for mouse femurs.

	Male						Female					
	+/+		W326R/+		Mutant vs WT		+/+		W326R/+		Mutant vs WT	
	n = 10		*n* = 6				*n* = 7		*n* = 5			
	Mean	s.d.	Mean	s.d.	% change	*P* value	Mean	s.d.	Mean	s.d.	% change	*P* value
Length (mm)	16.35	0.14	13.99	0.16	-14.5	3.11×10^{-14}	16.06	0.28	14.03	0.27	-12.6	$1.85E \times 10^{-7}$
Stiffness (N/mm)	68.24	15.31	56.41	18.15	-16.5	0.22	71.74	6.00	59.93	21.97	-16.5	0.20
F_{max} (N)	20.68	5.42	14.16	3.50	-30.8	0.028	22.87	3.65	16.82	3.31	-26.4	0.015
F_{break} (N)	13.43	5.71	12.09	4.39	-8.4	0.70	17.45	4.10	15.50	4.30	-11.2	0.44
W to F_{max} (Nmm)	5.93	2.19	3.56	0.54	-41.7	0.017	5.56	1.07	3.23	0.79	-42.0	0.0021
W to break (Nmm)	9.77	3.92	5.22	1.06	-46.9	0.019	9.07	3.13	3.64	0.57	-59.8	0.0036

Supplementary Figure 1

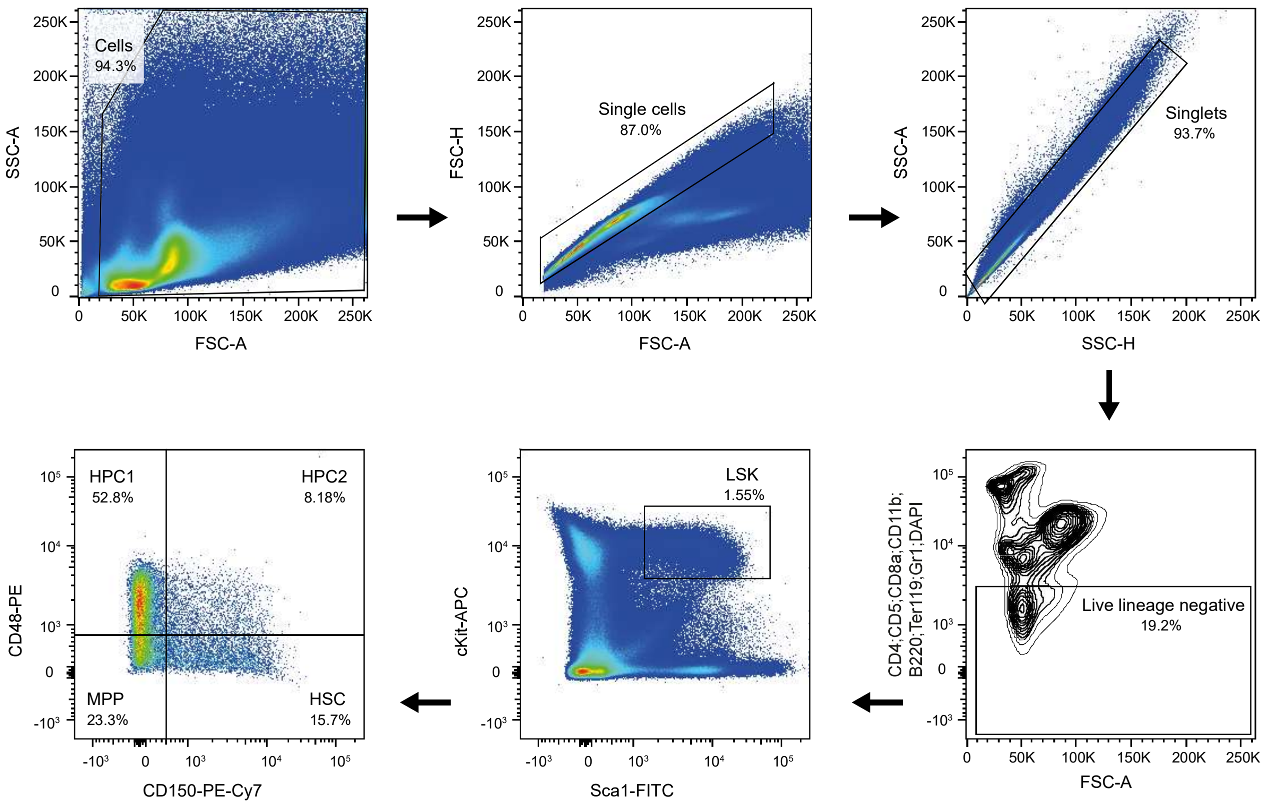

Supplementary Fig. 1| Representative gating strategy for Fig. 3b.

Supplementary Figure 2

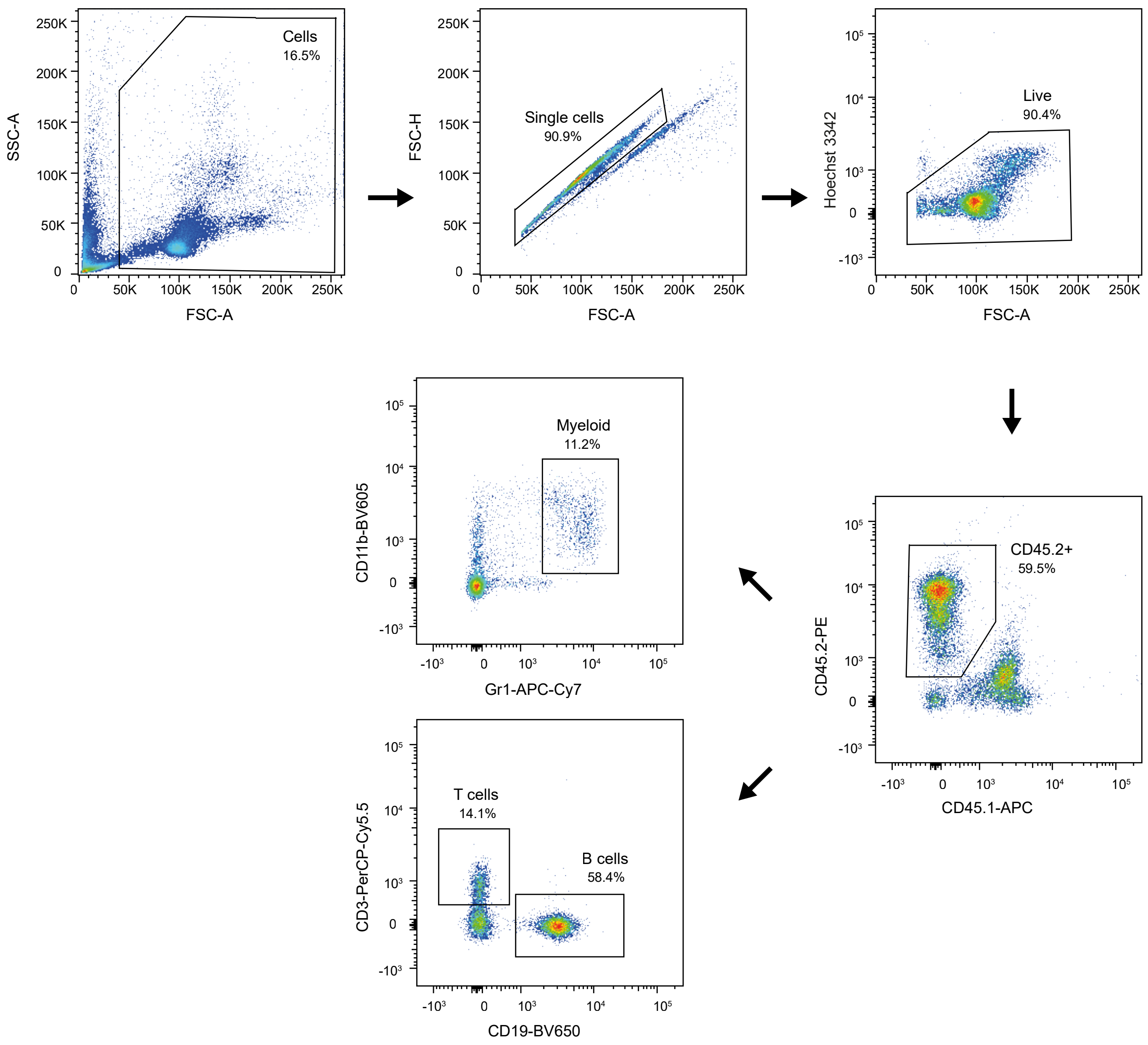

Supplementary Fig. 2| Representative gating strategy for Fig. 3d-g.

Supplementary Figure 3

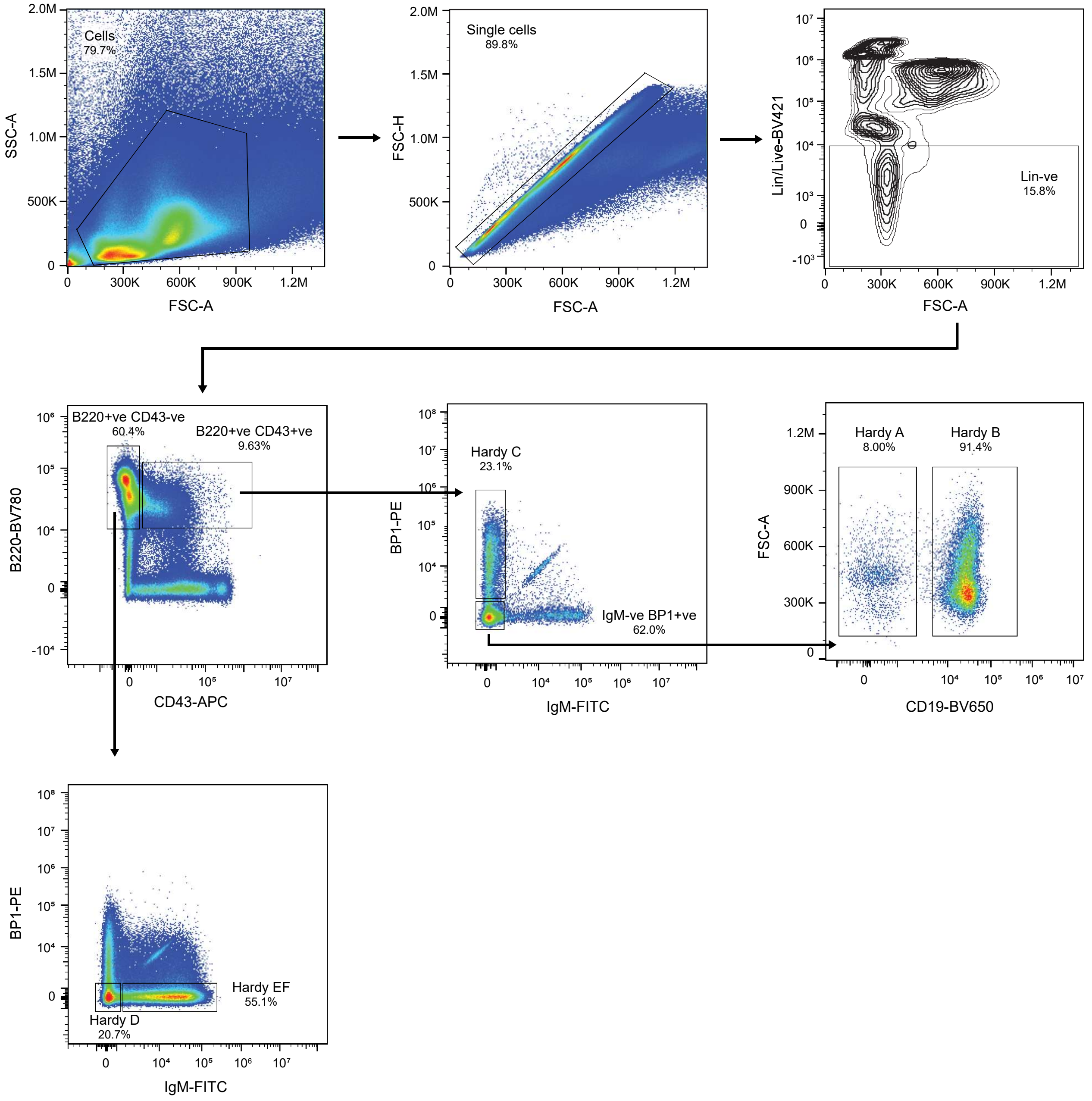

Supplementary Fig. 3| Representative gating strategy for Fig. 5c-d.
