# Supplementary material for: A progeria syndrome links DNA hypermethylation to age-related pathology
Source: Nat Genet. 2026 Jun 12;58(7):1632–42. doi: 10.1038/s41588-026-02633-8 (PMC13364717; doi:10.1038/s41588-026-02633-8)
Supplement: Supplementary file 2 [file 41588_2026_2633_MOESM2_ESM.pdf]

nature portfolio

Corresponding author(s): Andrew Jackson

Last updated by author(s): Mar 10, 2026

Reporting Summary

Nature Portfolio wishes to improve the reproducibility of the work that we publish. This form provides structure for consistency and transparency in reporting. For further information on Nature Portfolio policies, see our Editorial Policies and the Editorial Policy Checklist.

Statistics

For all statistical analyses, confirm that the following items are present in the figure legend, table legend, main text, or Methods section.

n/a	Confirmed	
☐	☒	The exact sample size (*n*) for each experimental group/condition, given as a discrete number and unit of measurement
☐	☒	A statement on whether measurements were taken from distinct samples or whether the same sample was measured repeatedly
☐	☒	The statistical test(s) used AND whether they are one- or two-sided<br>*Only common tests should be described solely by name; describe more complex techniques in the Methods section.*
☒	☐	A description of all covariates tested
☐	☒	A description of any assumptions or corrections, such as tests of normality and adjustment for multiple comparisons
☐	☒	A full description of the statistical parameters including central tendency (e.g. means) or other basic estimates (e.g. regression coefficient) AND variation (e.g. standard deviation) or associated estimates of uncertainty (e.g. confidence intervals)
☐	☒	For null hypothesis testing, the test statistic (e.g. *F*, *t*, *r*) with confidence intervals, effect sizes, degrees of freedom and *P* value noted<br>*Give P values as exact values whenever suitable.*
☒	☐	For Bayesian analysis, information on the choice of priors and Markov chain Monte Carlo settings
☒	☐	For hierarchical and complex designs, identification of the appropriate level for tests and full reporting of outcomes
☐	☒	Estimates of effect sizes (e.g. Cohen's *d*, Pearson's *r*), indicating how they were calculated

Our web collection on statistics for biologists contains articles on many of the points above.

Software and code

Policy information about availability of computer code

Data collection	BD FACSDiva software and Beckman Coulter CytExpert SRT (v1.1.0.10007) were used to collect flow cytometry data. Immunohistochemistry images were digitalised using a NanoZoomer Digital slide scanner and NDP.view2 software (Hamamatsu). Liver and adipose tissue slices were imaged with a Zeiss Axioscan.Z1 and Zen2.6 software. Open field and Y-maze tests were filmed using iSpy Agent DVR (v6.9.8.0) software.
Data analysis	General statistics or visualization: R (v4.4.1); GraphPad PRISM (v10); IGV (v2.16.0); FlowJo (v10); Microsoft Excel 2021<br><br>Structural modelling: PyMol (v2.4.0)<br><br>Histology: QuPath (v0.5.1 or v0.6.0); Bruker Skyscan Dataviewer (v1.5.6.6); Neoscan N80 software (v3.1.2); Bruker Skyscan CTAn (v1.20.8.0); Fiji (v1.54)<br><br>Neurobehavioural testing: ezTrack (v1.2), CalR (v2, https://calrapp.org/)<br><br>Sequencing data analysis: R (v4.4.1); FASTQC (v0.11.4); TrimGalore (v0.4.1); Bismark (v 0.18.1); Bowtie2 (v2.3.1); BEDtools (v2.30.0); UCSC tools bedGraphToBigWig (v369); minfi (v1.50.0); DSS (v2.54.0); deeptools (v3.5.4); ChIPpeakAnno (v3.40.0); GO.db (v3.20.0); GOSemSim (v2.32.0); org.Hs.eg.db (v3.20.0); glmnet (v4.1-8); seqtk (v1.4)<br><br>scRNA sequencing: analysis was performed using Python (v3.8.6 or v3.8.12) and R (v3.6.3 or v4.0.3). Softwares/packages used include: (For preprocessing) Cell Ranger (v6.0.1), scanpy (v1.6.0), scrublet (v0.2.1), umap-learn (v0.4.6), Seurat (v4.0.0), SeuratDisk (v0.0.0.9013); (For differential abundance analysis) scanpy (v1.6.0), meld (v1.0.0), scikit-learn (v1.0.1); (For differentiation trajectory analysis) scanpy (v1.8.0 and 1.8.1), scvelo (v0.2.4), cellrank (v1.5.1), scikit-learn (v0.22.1 and 0.24.2), scipy (v1.5.2); (For differential expression analysis) scanpy (v1.8.1)

See methods for further details.

For manuscripts utilizing custom algorithms or software that are central to the research but not yet described in published literature, software must be made available to editors and reviewers. We strongly encourage code deposition in a community repository (e.g. GitHub). See the Nature Portfolio guidelines for submitting code & software for further information.

Data

Policy information about availability of data

All manuscripts must include a data availability statement. This statement should provide the following information, where applicable:

- Accession codes, unique identifiers, or web links for publicly available datasets
- A description of any restrictions on data availability
- For clinical datasets or third party data, please ensure that the statement adheres to our policy

Processed and sequencing data generated as part of this study is available in GEO under accessions: GSE324236 (https://www.ncbi.nlm.nih.gov/geo/query/acc.cgi?acc=GSE324236) for Infinium methylation arrays, GSE324227 (https://www.ncbi.nlm.nih.gov/geo/query/acc.cgi?acc=GSE324227) for EM-seq, GSE324229 (https://www.ncbi.nlm.nih.gov/geo/query/acc.cgi?acc=GSE324229) for VDJ-seq and GSE319569 (https://www.ncbi.nlm.nih.gov/geo/query/acc.cgi?acc=GSE319569) for scRNA-seq.

Previously published data sets used in this study: mm10: GSM8027450, GSM8027453, GSM8027451, GSM8027448, and hg19: GSE31263, GSM772868, GSM997231, GSM997240, GSM772867, Generation Scotland. Generation Scotland data access requests, see 'GS Access Request Form' at https://www.ed.ac.uk/generation-scotland/for-researchers/access. Requests require approval by the Generation Scotland Access Committee to ensure compliance with participant consent.

Research involving human participants, their data, or biological material

Policy information about studies with human participants or human data. See also policy information about sex, gender (identity/presentation), and sexual orientation and race, ethnicity and racism.

Reporting on sex and gender	The term sex (biological attribute) was used in this study, based on self-reporting or assigned by the local clinician. Detailed patient characteristics for P1-P13 are documented in Supplementary Tables 1 and 2. Seven females and six males were recruited. Informed written consent was obtained from all participating families for sharing of individual-level data.
Reporting on race, ethnicity, or other socially relevant groupings	Race, ethnicity or socially relevant groupings were not reported in this study.
Population characteristics	Detailed patient characteristics for P1-P13 are documented in Supplementary Tables 1 and 2.
Recruitment	Patients were recruited to the research studies by their local clinician. Selection was based on genotype information. Informed written consent was obtained from all participating families. Participant compensation was not provided.
Ethics oversight	The research studies were approved by the Scottish Multicentre Research Ethics Committee (05/MRE00/74), the University of Utah medical review board (IRB00085855), the Bergen Hospital Trust ethical review board and the Ethics Committee of the Instituto de Salud Carlos III (CEI PI 93_2022).

Note that full information on the approval of the study protocol must also be provided in the manuscript.

Field-specific reporting

Please select the one below that is the best fit for your research. If you are not sure, read the appropriate sections before making your selection.

☒ Life sciences ☐ Behavioural & social sciences ☐ Ecological, evolutionary & environmental sciences

For a reference copy of the document with all sections, see nature.com/documents/nr-reporting-summary-flat.pdf

Life sciences study design

All studies must disclose on these points even when the disclosure is negative.

Sample size	No statistical methods were used to pre-determine sample size. Sample sizes were chosen based on standard practices of the field; or sample/data availability.
Data exclusions	Reverse transplantation - for two recipients (1x WT; 1x Dnmt3a W326R/+), tail vein injection was incomplete - and so these animals were excluded from analyses. Subcutaneous adipose tissue measurements - for two 13-month-old mutant males the quality of tissue sections was very poor - and so these samples were excluded from analysis. Knee histopathology analysis - one WT male unexpectedly showed features resembling severe medial compartmental OA (complete erosion of cartilage and extensive remodelling of surrounding tissues); this sample was considered an outlier and excluded from all analyses.

Replication | All attempts at replication were successful. Independent experiments were performed to ensure reproducibility as documented in Figure legends and methods.

Randomization | No methods of randomization were used. Samples/organisms/participants were allocated to groups on basis of genotypes (i.e. Mendelian randomization for organisms and participants).

Blinding | Investigators were blinded to genotypes/group allocations during data collection and analyses.

Reporting for specific materials, systems and methods

We require information from authors about some types of materials, experimental systems and methods used in many studies. Here, indicate whether each material, system or method listed is relevant to your study. If you are not sure if a list item applies to your research, read the appropriate section before selecting a response.

Materials & experimental systems

n/a	Involved in the study	
☐	☒	Antibodies
☒	☐	Eukaryotic cell lines
☒	☐	Palaeontology and archaeology
☐	☒	Animals and other organisms
☐	☒	Clinical data
☒	☐	Dual use research of concern
☒	☐	Plants

Methods

n/a	Involved in the study	
☒	☐	ChIP-seq
☐	☒	Flow cytometry
☒	☐	MRI-based neuroimaging

Antibodies

Antibodies used

Flow cytometry analysis of steady state BM cells, differentiated cells:
CD4 Biotin (BD Biosciences 553648, lot 7054665)
CD8a Biotin (BD Biosciences 553028, lot 2112287)
CD11b BV421 (BioLegend 101223, lot B386941)
B220 BV786 (BD Biosciences 563894, lot 3298121)
CD71 FITC (Invitrogen 11-0711-82, lot 2640485)
Gr1 PE (BD Biosciences 551461, lot 3221831)

Flow cytometry analysis of steady state BM cells (progenitor cells) and HSC cell sort for EM-seq:
CD4 Biotin (BD Biosciences 553648, lot 7054665)
CD5 Biotin (BD Biosciences 553018, lot 0156529)
CD8a Biotin (BD Biosciences 553028, lot 2112287)
CD11b Biotin (BD Biosciences 557395, lot 0350634)
B220 Biotin (BD Biosciences 553086, lot 2094412)
Ter119 Biotin (BD Biosciences 553672, lot 2115202)
Gr-1 Biotin (BD Biosciences 553125, lot 1060384)
Sca1 FITC (Biolegend 122506, lot B277817)
CD48 Pe (BD Biosciences 103405, lot B359894)
CD150 Pe-Cy7 (BD Biosciences 115914, lot B382747)
c-KIT APC (BD Biosciences 105812, lot B381643)
Streptavidin-Pacific Blue (Molecular probes, S11222)

HSC sort for BM transplantation and HSPC sort for scRNA-seq:
CD3 PE (Biolegend 100205)
B220 PE (BD Biosciences 553089)
Gr1 PE (BD Biosciences 553128)
Ter119 PE (BD Biosciences 553673)
CD11b PE (BD Biosciences 553311)
NK1.1 AF700 (Biolegend 108730)
Sca1 PeCy (eBioscience 25-5981-81)
CD48 AF700 (Biolegend 103425)
c-Kit BV421 (BD Biosciences 562609)
CD150 BV605 (Biolegend 115927)

Post-transplantation analysis, donor cell chimerism and multilineage reconstitution:
CD45.1 APC (BD Biosciences 558701, lot 3138612)
CD45.2 PE (BD Biosciences 560695, lot 3201722),
CD3 PerCP-Cy5.5 (eBioscience 45-0031-82, lot 2527402)
CD11b BV605 (BioLegend 101237, lot B370143)
CD19 BV650 (BioLegend 115541, lot B386727)
Gr1 APC-Cy7 (BD Biosciences 557661, lot 3200110)
Ter119 BV421 (BD Biosciences 563998, lot 3291972)

Post-transplantation analysis, progenitor cell population:
Same panel as for HSC sort for BM transplantation, but including
CD45.2-AF647 (BioLegend 109818)

Intestinal regeneration assay:
anti-BrdU (BD Biosciences 347580, lot 3016583 and 9172603)

Immature B cell sort for Pax RT-qPCR and EM conversion PCR:
Ter119 BV421 (1:200; Invitrogen 404-5921-82, lot 2827616)
CD4 BV421 (1:1600; Invitrogen 404-0042-82, lot 2823734)
CD8 (1:200; Invitrogen 404-0081-82, lot 2977590)
NK1.1 BV421 (1:40; Biolegend 108741, lot B427542)
Gr-1 BV421 (1:200; BD Biosciences 562709, lot 3255198)
CD19 BV650 (1:20; Biolegend 115541, lot B454588)
B220 BV786 (1:200; BD Biosciences 563894, lot 4305767)
IgM FITC (1:100; Invitrogen 11-5790-81, lot 2839732)
BP-1 PE (1:50; Invitrogen 12-5891-81, lot 2789871)
CD43 APC (1:200: BD Biosciences 56066, lot 52265613)

Pro-B cell sort for VDJ-seq:
B220 APC (1:200; Biolegend 103211, lot B271009)
CD43 PeCy7 (1:200; BD Biosciences 562866, lot 9326901)
IgM PerCP-eFluor710 (1:200, Themo Fisher Scientific 46-5790-82, lot 2185098).

Flow cytometry analysis of B cell differentiation:
B220 AF700 (1:65; Biolegend 103232, lot B428206)
IgM FITC (1:100; Invitrogen 11-5790-81, lot 2839732)
BP-1 PE (1:50; Invitrogen 12-5891-81, lot 2789871)
CD19 PE-Cy7 (1:200; BD Biosciences 552854, lot 3222461)
CD43 APC (1:200; BD Biosciences 560663, lot 5226561)

Validation

All flow cytometry antibodies were validated and quality tested as shown on the manufacturer website, often including a histogram of positive and negative cells stained with the respective antibody. Prior to using each antibody, we performed testing on the cell types of interest and/or on single stain beads to determine specificity and sensitivity.

BD Biosciences quality and reproducibility statement: https://www.bdbiosciences.com/en-gb/products/reagents/flow-cytometry-reagents/research-reagents/quality-and-reproducibility

Biolegend quality and reproducibility statement: https://www.biolegend.com/ja-jp/quality/quality-control

ThermoFisher Scientific (Invitrogen) quality and reproducibility statement: https://www.thermofisher.com/uk/en/home/life-science/antibodies/invitrogen-antibody-validation.html

CD4 Biotin (BD Biosciences 553648, lot 7054665), BD Pharmingen™ Biotin Rat Anti-Mouse CD4, https://www.bdbiosciences.com/en-gb/products/reagents/flow-cytometry-reagents/research-reagents/single-color-antibodies-ruo/biotin-rat-anti-mouse-cd4.553649?tab=product_details

CD8a Biotin (BD Biosciences 553028, lot 2112287), BD Pharmingen™ Biotin Rat Anti-Mouse CD8a, https://www.bdbiosciences.com/en-gb/products/reagents/cell-preparation-separation-reagents/biotin-rat-anti-mouse-cd8a.553028?tab=format_details

CD11b BV421 (BioLegend 101223, lot B386941), Pacific Blue™ anti-mouse/human CD11b Antibody, https://www.biolegend.com/en-gb/products/pacific-blue-anti-mouse-human-cd11b-antibody-3863

B220 BV786 (BD Biosciences 563894, lot 3298121), BD Horizon™ BV786 Rat Anti-Mouse CD45R/B220, https://www.bdbiosciences.com/en-gb/products/reagents/flow-cytometry-reagents/research-reagents/single-color-antibodies-ruo/bv786-rat-anti-mouse-cd45r-b220.563894?tab=product_details

CD71 FITC (Invitrogen 11-0711-82, lot 2640485), CD71 (Transferrin Receptor) Monoclonal Antibody (R17217 (RI7 217.1.4)), FITC, eBioscience™, https://www.thermofisher.com/antibody/product/CD71-Transferrin-Receptor-Antibody-clone-R17217-RI7-217-1-4-Monoclonal/11-0711-82

Gr1 PE (BD Biosciences 551461, lot 3221831), BD Pharmingen™ PE Rat Anti-Mouse Ly-6G, https://www.bdbiosciences.com/en-us/products/reagents/flow-cytometry-reagents/research-reagents/single-color-antibodies-ruo/pe-rat-anti-mouse-ly-6g.551461?tab=product_details

CD4 Biotin (BD Biosciences 553648, lot 7054665), CD4 Rat anti-Mouse, Biotin, Clone: H129.19, BD, https://www.bdbiosciences.com/en-gb/products/reagents/flow-cytometry-reagents/research-reagents/single-color-antibodies-ruo/biotin-rat-anti-mouse-cd4.553649?tab=product_details

CD5 Biotin (BD Biosciences 553018, lot 0156529), BD Pharmingen™ Biotin Rat Anti-Mouse CD5, https://www.bdbiosciences.com/en-au/products/reagents/flow-cytometry-reagents/research-reagents/single-color-antibodies-ruo/biotin-rat-anti-mouse-cd5.553018?tab=product_details

CD8a Biotin (BD Biosciences 553028, lot 2112287), BD Pharmingen™ Biotin Rat Anti-Mouse CD8a, https://www.bdbiosciences.com/en-gb/products/reagents/cell-preparation-separation-reagents/biotin-rat-anti-mouse-cd8a.553028?tab=product_details

CD11b Biotin (BD Biosciences 557395, lot 0350634), BD Pharmingen™ Biotin Rat Anti-CD11b, https://www.bdbiosciences.com/en-us/products/reagents/flow-cytometry-reagents/research-reagents/single-color-antibodies-ruo/biotin-rat-anti-cd11b.557395?tab=product_details

B220 Biotin (BD Biosciences 553086, lot 2094412), BD Pharmingen™ Biotin Rat Anti-Mouse CD45R/B220, https://www.bdbiosciences.com/en-gb/products/reagents/cell-preparation-separation-reagents/biotin-rat-anti-mouse-cd45r-b220.553086?tab=product_details

Ter119 Biotin (BD Biosciences 553672, lot 2115202), BD Pharmingen™ Biotin Rat Anti-Mouse TER-119/Erythroid Cells, https://www.bdbiosciences.com/en-gb/products/reagents/flow-cytometry-reagents/research-reagents/single-color-antibodies-ruo/biotin-rat-anti-mouse-ter-119-erythroid-cells.553672?tab=product_details

Gr-1 Biotin (BD Biosciences 553125, lot 1060384), BD Pharmingen™ Biotin Rat Anti-Mouse Ly-6G and Ly-6C, https://www.bdbiosciences.com/en-gb/products/reagents/cell-preparation-separation-reagents/biotin-rat-anti-mouse-ly-6g-and-ly-6c.553125?tab=product_details

Sca1 FITC (Biolegend 122506, lot B277817), FITC anti-mouse Ly-6A/E (Sca-1) Antibody, https://www.biolegend.com/en-us/products/fitc-anti-mouse-ly-6a-e-sca-1-antibody-3894

CD48 Pe (BD Biosciences 103405, lot B359894), PE anti-mouse CD48 Antibody, https://www.biolegend.com/en-gb/products/pe-anti-mouse-cd48-antibody-293

CD150 Pe-Cy7 (BD Biosciences 115914, lot B382747), PE/Cyanine7 anti-mouse CD150 (SLAM) Antibody, https://www.biolegend.com/en-us/products/pe-cyanine7-anti-mouse-cd150-slam-antibody-3056?lang=uk

c-KIT APC (BD Biosciences 105812, lot B381643), APC anti-mouse CD117 (c-Kit) Antibody, https://www.biolegend.com/en-us/products/apc-anti-mouse-cd117-c-kit-antibody-72

CD3 PE (Biolegend 100205), PE anti-mouse CD3 Antibodyhttps://www.biolegend.com/en-gb/products/pe-anti-mouse-cd3-antibody-47

B220 PE (BD Biosciences 553089), BD Pharmingen™ PE Rat Anti-Mouse CD45R/B220 https://www.bdbiosciences.com/en-ca/products/reagents/flow-cytometry-reagents/research-reagents/single-color-antibodies-ruo/pe-rat-anti-mouse-cd45r-b220.553089?tab=product_details

Gr1 PE (BD Biosciences 553128), BD Pharmingen™ PE Rat Anti-Mouse Ly-6G and Ly-6C, https://www.bdbiosciences.com/en-gb/products/reagents/flow-cytometry-reagents/research-reagents/single-color-antibodies-ruo/pe-rat-anti-mouse-ly-6g-and-ly-6c.553128?tab=product_details

Ter119 PE (BD Biosciences 553673), BD Pharmingen™ PE Rat Anti-Mouse TER-119/Erythroid Cells, https://www.bdbiosciences.com/en-gb/products/reagents/flow-cytometry-reagents/research-reagents/single-color-antibodies-ruo/pe-rat-anti-mouse-ter-119-erythroid-cells.553673?tab=product_details

CD11b PE (BD Biosciences 553311), BD Pharmingen™ PE Rat Anti-CD11b, https://www.bdbiosciences.com/en-gb/products/reagents/flow-cytometry-reagents/research-reagents/single-color-antibodies-ruo/pe-rat-anti-cd11b.553311?tab=product_details

NK1.1 AF700 (Biolegend 108730), Alexa Fluor® 700 anti-mouse NK-1.1 Antibody, https://www.biolegend.com/en-gb/products/alexa-fluor-700-anti-mouse-nk-1-1-antibody-6555

Sca1 PeCy (eBioscience 25-5981-81), Ly-6A/E (Sca-1) Monoclonal Antibody (D7), PE-Cyanine7, eBioscience™, Invitrogen™, https://www.fishersci.co.uk/shop/products/ly-6a-e-sca-1-monoclonal-antibody-d7-pe-cyanine7-ebioscience-invitrogen/p-7090472

CD48 AF700 (Biolegend 103425), Alexa Fluor® 700 anti-mouse CD48 Antibody, https://www.biolegend.com/en-gb/products/alexa-fluor-700-anti-mouse-cd48-antibody-6670

c-Kit BV421 (BD Biosciences 562609), BD Horizon™ BV421 Rat Anti-Mouse CD117, https://www.bdbiosciences.com/en-gb/products/reagents/flow-cytometry-reagents/research-reagents/single-color-antibodies-ruo/bv421-rat-anti-mouse-cd117.562609?tab=product_details

CD150 BV605 (Biolegend 115927), Brilliant Violet 605™ anti-mouse CD150 (SLAM) Antibody, https://www.biolegend.com/en-gb/products/brilliant-violet-605-anti-mouse-cd150-slam-antibody-7871

CD45.1 APC (BD Biosciences 558701, lot 3138612), BD Pharmingen™ APC Mouse anti-Mouse CD45.1, https://www.bdbiosciences.com/en-us/products/reagents/flow-cytometry-reagents/research-reagents/single-color-antibodies-ruo/apc-mouse-anti-mouse-cd45-1.558701?tab=product_details

CD45.2 PE (BD Biosciences 560695, lot 3201722), BD Pharmingen™ PE Mouse Anti-Mouse CD45.2, https://www.bdbiosciences.com/en-eu/products/reagents/flow-cytometry-reagents/research-reagents/single-color-antibodies-ruo/pe-mouse-anti-mouse-cd45-2.560695?tab=product_details

CD3 PerCP-Cy5.5 (eBioscience 45-0031-82, lot 2527402), CD3e Monoclonal Antibody (145-2C11), PerCP-Cyanine5.5, eBioscience™, https://www.thermofisher.com/antibody/product/CD3e-Antibody-clone-145-2C11-Monoclonal/45-0031-82

CD11b BV605 (BioLegend 101237, lot B370143), Brilliant Violet 605™ anti-mouse/human CD11b Antibody, https://www.biolegend.com/en-gb/products/brilliant-violet-605-anti-mouse-human-cd11b-antibody-7637

CD19 BV650 (BioLegend 115541, lot B386727), Brilliant Violet 650™ anti-mouse CD19 Antibody, https://www.biolegend.com/en-gb/products/brilliant-violet-650-anti-mouse-cd19-antibody-7851

Gr1 APC-Cy7 (BD Biosciences 557661, lot 3200110), BD Pharmingen™ APC-Cy™7 Rat Anti-Mouse Ly-6G and Ly-6C, https://www.bdbiosciences.com/en-gb/products/reagents/flow-cytometry-reagents/research-reagents/single-color-antibodies-ruo/apc-cy-7-rat-anti-mouse-ly-6g-and-ly-6c.557661?tab=product_details

Ter119 BV421 (BD Biosciences 563998, lot 3291972), BD Horizon™ BV421 Rat Anti-Mouse TER-119/Erythroid Cells, https://www.bdbiosciences.com/en-gb/products/reagents/flow-cytometry-reagents/research-reagents/single-color-antibodies-ruo/bv421-rat-anti-mouse-ter-119-erythroid-cells.563998?tab=product_details

CD45.2-AF647 (BioLegend 109818), Alexa Fluor® 647 anti-mouse CD45.2 Antibody, https://www.biolegend.com/en-gb/products/alexa-fluor-647-anti-mouse-cd45-2-antibody-3107?GroupID=BLG1934

Streptavidin-Pacific Blue (Molecular probes, S11222), Streptavidin, Pacific Blue™ conjugate, https://www.thermofisher.com/order/catalog/product/S11222

anti-BrdU (BD Biosciences 347580, lot 3016583), BD™ Purified Mouse Anti-BrdU, https://www.bdbiosciences.com/en-gb/products/reagents/flow-cytometry-reagents/clinical-discovery-research/single-color-antibodies-ruo-gmp/purified-mouse-anti-brdu.347580?tab=product_details

Ter119 BV421 (1:200; Invitrogen 404-5921-82), Invitrogen TER-119 Monoclonal Antibody (TER-119), Brilliant Violet™ 421, eBioscience™, https://www.thermofisher.com/antibody/product/TER-119-Antibody-clone-TER-119-Monoclonal/404-5921-82

CD4 BV421 (1:1600; Invitrogen 404-0042-82), Invitrogen CD4 Monoclonal Antibody (RM4-5), Brilliant Violet™ 421, eBioscience™, https://www.thermofisher.com/antibody/product/CD4-Antibody-clone-RM4-5-Monoclonal/404-0042-82

CD8 (1:200; Invitrogen 404-0081-82), Invitrogen CD8a Monoclonal Antibody (53-6.7), Brilliant Violet™ 421, eBioscience™, https://www.thermofisher.com/antibody/product/CD8a-Antibody-clone-53-6-7-Monoclonal/404-0081-82

NK1.1 BV421 (1:40; Biolegend 108741), Biolegend Brilliant Violet 421™ anti-mouse NK-1.1 Antibody, https://www.biolegend.com/en-gb/products/brilliant-violet-421-anti-mouse-nk-1-1-antibody-7150

Gr-1 BV421 (1:200; BD Biosciences 562709), BD Horizon™ BV421 Rat Anti-Mouse Ly-6G and Ly-6C Clone RB6-8C5 (RUO), https://www.bdbiosciences.com/en-eu/products/reagents/microscopy-imaging-reagents/immunofluorescence-reagents/bv421-rat-anti-mouse-ly-6g-and-ly-6c.562709?tab=product_details

IgM FITC (1:100; Invitrogen 11-5790-81), Invitrogen Mouse IgM Monoclonal Antibody (II/41), FITC, eBioscience™, https://www.thermofisher.com/antibody/product/Mouse-IgM-Antibody-clone-II-41-Monoclonal/11-5790-81

BP-1 PE (1:50; Invitrogen 12-5891-81), Invitrogen CD249 (BP-1) Monoclonal Antibody (6C3), Biotin, eBioscience™, https://www.thermofisher.com/antibody/product/CD249-BP-1-Antibody-clone-6C3-Monoclonal/13-5891-81

CD43 APC (1:200: BD Biosciences 560663), BD Pharmingen™ APC Rat Anti-Mouse CD43 Clone S7 (RUO), https://www.bdbiosciences.com/en-gb/products/reagents/flow-cytometry-reagents/research-reagents/single-color-antibodies-ruo/apc-rat-anti-mouse-cd43.560663?tab=product_details

B220 AF700 (1:65; Biolegend 103232), Biolegend Alexa Fluor® 700 anti-mouse/human CD45R/B220 Antibody, https://www.biolegend.com/en-gb/products/alexa-fluor-700-anti-mouse-human-cd45r-b220-antibody-3408

B220 APC (1:200; Biolegend 103211, lot B271009), Biolegend APC anti-mouse/human CD45R/B220 Antibody, https://www.biolegend.com/fr-ch/products/apc-anti-mouse-human-cd45r-b220-antibody-442?GroupID=GROUP658

CD43 PeCy7 (1:200; BD Biosciences 562866), BD Pharmingen™ PE-Cy™7 Rat Anti-Mouse CD43 Clone S7 (RUO), https://www.bdbiosciences.com/en-eu/products/reagents/flow-cytometry-reagents/research-reagents/single-color-antibodies-ruo/pe-cy-7-rat-anti-mouse-cd43.562866?tab=product_details

IgM PerCP-eFluor710 (1:200, Themo Fisher Scientific 46-5790-82, lot 2185098), Invitrogen Mouse IgM Monoclonal Antibody (II/41), PerCP-eFluor™ 710, eBioscience™, https://www.thermofisher.com/antibody/product/Mouse-IgM-Antibody-clone-II-41-Monoclonal/46-5790-82

Animals and other research organisms

Policy information about studies involving animals; ARRIVE guidelines recommended for reporting animal research, and Sex and Gender in Research

Laboratory animals	Genetically modified mice carrying the Dnmt3a-W326R mutation were generated and maintained on a C57Bl/6JCrl background. The age of mice used for data collection are indicated in figure legends.
Wild animals	N/A. This study did not involve wild animals.
Reporting on sex	None of the findings presented were sex-specific, although there may be some differences between the sexes in terms of time of

Reporting on sex	onset or overall severity. Data for bone and metabolic phenotypes are presented for both sexes. Figure legends indicate the sex of animals used.
Field-collected samples	N/A. This study did not involve samples collected from the field.
Ethics oversight	All experimental protocols were approved by the University of Edinburgh animal welfare and ethical review board and performed under a UK Home Office-approved Project License, in compliance with the UK Home Office Scientific Procedure (Animals) Act 1983. Breeding, maintenance and general phenotyping were performed under Project License P2A477A62, PP1567597 and PP2060675, with bone marrow transplants and oxaliplatin treatments performed under Project License PC6D479E0 and PP9016178, respectively.

Note that full information on the approval of the study protocol must also be provided in the manuscript.

Clinical data

Policy information about clinical studies
All manuscripts should comply with the ICMJE guidelines for publication of clinical research and a completed CONSORT checklist must be included with all submissions.

Clinical trial registration	N/A
Study protocol	This study is not a clinical trial. The research study protocol can be accessed by contacting the corresponding author (APJ).
Data collection	Patients were recruited to the research studies by their local clinician. Selection was based on genotype information. Clinical data was collected by the local clinician for each participant. Informed written consent was obtained from all participating families.
Outcomes	N/A

Plants

Seed stocks	N/A
Novel plant genotypes	N/A
Authentication	N/A

Flow Cytometry

Plots

Confirm that:

☒ The axis labels state the marker and fluorochrome used (e.g. CD4-FITC).

☒ The axis scales are clearly visible. Include numbers along axes only for bottom left plot of group (a 'group' is an analysis of identical markers).

☒ All plots are contour plots with outliers or pseudocolor plots.

☒ A numerical value for number of cells or percentage (with statistics) is provided.

Methodology

Sample preparation	Described in detail in Methods: Flow Cytometry, Cell Sorting, VDJ-Seq and Bone marrow transplantation assays.
Instrument	5-laser LSRFortessa SORP Cell Analyzer, BD FACSAria II (BD Biosciences) or Beckman Coulter Cytoflex SRT (4 laser)
Software	BD FACSDiva software or Beckman Coulter CytExpert SRT software (v1.1.0.10007) was used to acquire flow cytometry data. FlowJo (v10) was used to analyse flow cytometry data.
Cell population abundance	To sort HSCs for transplantation and EM-seq, to sort HSPCs for scRNA-seq and to assess steady state progenitor cells in the bone marrow, up to 4 million events corresponding to single cells were acquired for each sample. To assess chimerism and cell differentiation post-transplantation, a minimum target of 10,000 events corresponding to donor-derived single cells was used per sample. To evaluate steady state differentiated bone marrow cells, more than 10,000 events corresponding to single cells were recorded per sample.
Gating strategy	For all gating strategies, singlets were identified based on FSC-A/FSC-H. Dead cell exclusion was performed by staining with Hoechst, DAPI or LIVE/DEAD Fixable Yellow Dead Cell Stain (ThermoFisher Scientific, L34959). Lineage panel comprised B220,

Gr1, Ter-119, CD11b and CD3 (bone marrow transplant and scRNA-seq experiments) or CD4, 5 and 8 (steady state BM analysis and HSC sort for EM-seq). NK1.1 was added to the lineage panel to sort HSCs for bone marrow transplant experiments. LSK cells were defined as Lineage- Sca1+ c-Kit+. HSCs were defined as Lineage- Sca1+ c-Kit+ CD48-CD150+. MPP cells were defined as Lineage- Sca1+ c-Kit+ CD48-CD150-. Myeloid cells were defined as CD11b+ Gr1+ cells. T cells were defined as CD3+ or CD4+CD8+ cells. B cells were defined as CD19+ or B220+ cells. Erythroid cells were defined as CD71+ cells. For bone marrow transplant experiments, donor versus recipient cells were distinguished by their CD45.1 and CD45.2 positivity. Thresholds for positivity were established using fluorescence-minus-one and/or unstained samples. Gates were always applied consistently across samples per experiment. Representative gating strategies are shown in Supplementary Fig. 1-3.

☒ Tick this box to confirm that a figure exemplifying the gating strategy is provided in the Supplementary Information.
