# Supplementary material for: A progeria syndrome links DNA hypermethylation to age-related pathology
Source: Nat Genet. 2026 Jun 12;58(7):1632–42. doi: 10.1038/s41588-026-02633-8 (PMC13364717; doi:10.1038/s41588-026-02633-8)
Supplement: Supplementary file 3 [file 41588_2026_2633_MOESM3_ESM.pdf]

nature portfolio

Peer Review File

A progeria syndrome links DNA hypermethylation to age-related pathology

Corresponding Author: Professor Andrew Jackson

This file contains all editorial decision letters in order by version, followed by all author rebuttals in order by version.

Version 0:

Decision Letter:

30th May 2025

Dear Professor Jackson,

Your Article, "DNA hypermethylation is causal for multiple age-related pathologies" has now been seen by 3 referees. You will see from their comments copied below that while they find your work of considerable potential interest, they have raised quite substantial concerns that must be addressed. In light of these comments, we cannot accept the manuscript for publication, but would be very interested in considering a revised version that addresses these serious concerns.

We hope you will find the referees' comments useful as you decide how to proceed. If you wish to submit a substantially revised manuscript, please bear in mind that we will be reluctant to approach the referees again in the absence of major revisions.

To guide the scope of the revisions, the editors discuss the referee reports in detail within the team, including with the chief editor, with a view to identifying key priorities that should be addressed in revision and sometimes overruling referee requests that are deemed beyond the scope of the current study. In this case, we ask that you address the reviewers' comments in full, with a particular focus on strengthening the evidence supporting your conclusions with more mechanistic insights. Please do not hesitate to get in touch if you would like to discuss these issues further.

If you choose to revise your manuscript taking into account all reviewer and editor comments, please highlight all changes in the manuscript text file. At this stage we will need you to upload a copy of the manuscript in MS Word .docx or similar editable format.

We are committed to providing a fair and constructive peer-review process. Do not hesitate to contact us if there are specific requests from the reviewers that you believe are technically impossible or unlikely to yield a meaningful outcome.

If revising your manuscript:

*1) Include a “Response to referees” document detailing, point-by-point, how you addressed each referee comment. If no action was taken to address a point, you must provide a compelling argument. This response will be sent back to the referees along with the revised manuscript.

*2) If you have not done so already please begin to revise your manuscript so that it conforms to our Article format instructions, available here.
Refer also to any guidelines provided in this letter.

*3) Include a revised version of any required Reporting Summary: https://www.nature.com/documents/nr-reporting-summary.pdf
It will be available to referees (and, potentially, statisticians) to aid in their evaluation if the manuscript goes back for peer review.
A revised checklist is essential for re-review of the paper.

Please be aware of our guidelines on digital image standards.

EXTENDED DATA FIGURES

When re-submitting your manuscript, please ensure that any supplementary figures and tables that are crucial to the manuscript's conclusions are converted into Extended Data figures and tables to increase visibility of these data. Extended Data figures and tables are online-only (present in the online PDF and full-text HTML versions of the paper), peer-reviewed display items that provide essential background to the article but are not included in the main article due to space constraints. A maximum of ten Extended Data display items (figures and tables) is permitted.

You may use the link below to submit your revised manuscript and related files:

Link Redacted

Note: This URL links to your confidential home page and associated information about manuscripts you may have submitted, or that you are reviewing for us. If you wish to forward this email to co-authors, please delete the link to your homepage.

If you wish to submit a suitably revised manuscript we would hope to receive it within 6 months. If you cannot send it within this time, please let us know. We will be happy to consider your revision so long as nothing similar has been accepted for publication at Nature Genetics or published elsewhere. Should your manuscript be substantially delayed without notifying us in advance and your article is eventually published, the received date would be that of the revised, not the original, version.

Please do not hesitate to contact me if you have any questions or would like to discuss the required revisions further.

Nature Genetics is committed to improving transparency in authorship. As part of our efforts in this direction, we are now requesting that all authors identified as 'corresponding author' on published papers create and link their Open Researcher and Contributor Identifier (ORCID) with their account on the Manuscript Tracking System (MTS), prior to acceptance. ORCID helps the scientific community achieve unambiguous attribution of all scholarly contributions. You can create and link your ORCID from the home page of the MTS by clicking on 'Modify my Springer Nature account'. For more information please visit please visit www.springernature.com/orcid.

Thank you for the opportunity to review your work.

Sincerely,
Chiara

Chiara Anania, PhD
Associate Editor
Nature Genetics
https://orcid.org/0000-0003-1549-4157

Referee expertise:

Referee #1: aging and genomics

Referee #2: methylation, transcription

Referee #3: epigenetics

Reviewers' Comments:

Reviewer #1 (Remarks to the Author):

In this work, the authors show that patients with DNMT3A gain-of-function mutations as well as a mouse model exhibit features of accelerated ageing. They also show that region-specific DNA
hypermethylation leads to stem cell dysfunction, suggesting that stem cell exhaustion drives the observed ageing phenotypes. This is a fascinating, original and timely work that provides causal evidence for a role of DNA methylation in ageing. I only have a few comments and suggestions.

My major issue is that from an ageing perspective some additional detail/information would be valuable. Regarding the HESJAS patients, my understanding from figure 1A is that these are relatively young patients. I would be interested in knowing the age range of these patients, also whether there is any indication of premature death - from what I could gather on page 5 only one patient has died so far, is that correct?

Likewise, I wonder what is the lifespan of the mutant mice or, at least, if there is evidence of premature mortality? If so, what

do the animals die of?

Although there is a mention to epigenetic clocks (page 8), it was not clear to me whether epigenetic clocks are accelerated in HESJAS patients. And what about epigenetic clocks in the mouse model?

Lastly, given that wild type Dnmt3a hinders stem cell renewal, I wonder what is its evolutionary purpose? In other words, what is the downside of Dnmt3a? I appreciate this a more evolutionary perspective outside the scope of the work, but I would be interested in the authors thoughts on this.

Overall, I think this is a fascinating work that will make an important contribution to the field, though a few aspects could be improved/clarified.

It is possible that some of my comments reflect misunderstandings of mine. If so then I would suggest that the authors use my misunderstandings as an indication that such points might be made clearer in the manuscript.

It is my usual policy to reveal my identity to the authors: Joao Pedro de Magalhaes.

Reviewer #2 (Remarks to the Author):

NG-A68576 ms review, Neary et al., DNA hypermethylation is causal for multiple age-related pathologies

Description
In this manuscript, Neary et al. utilize a DNMT3A gain-of-functon(GOF) mutant mouse model to study the role of aberrant DNA hypermethylation during aging. The DNMT3A GOF mutation of interest (W326R in mouse DNMT3A) is found within the PWWP domain, and results in relocalization of DNMT3A away from H3K36me2/3-marked regions into Polycomb-marked regions, with a corresponding gain of DNA methylation in such regions. This DNMT3A GOF mutation is similar to the ones observed in patients with Heyn-Sproul-Jackson syndrome (HESJAS), which is characterized by global developmental delay. Since the DNMT3A GOF mouse model displays DNA hypermethylation at Polycomb-marked regions in several tissues, as has been previously reported to occur during aging in somatic mitotic cells (PMID: 39009581), the authors propose that this mouse model would allow them to establish a causality between DNA hypermethylation at Polycomb-marked regions and ageing.
However, the data currently presented in this manuscript is insufficient to support such a statement. The manuscript is largely missing measurements of aging at the organismal level in the DNMT3A GOF mouse model. Moreover, several of the authors' conclusion regarding DNA methylation changes are based on analysis of a limited subset of CpGs mostly overlapping with promoters and enhancers (obtained using Infinium Methylation Arrays), and their analysis of whole-genome methylation data is similarly biased towards those regions. Additional experiments and considerations are needed to clarify the mechanism proposed in the title ("DNA hypermethylation is causal for multiple age-related pathologies"). Alternatively, the title of this manuscript should be revised to better reflect the conclusions that can reasonably be drawn from the presented data.

Major comments
#1. Although the manuscript aims to present a mouse model of aging, there is a lack of measurements of aging at the organism level in the DNMT3A GOF mouse model. For example, while Fig. 2a suggests that the DNMT3A GOF mouse model displays a similar developmental delay during the first 9 wk of life as is observed in HESJAS patients, in lines 195-196 of the manuscript, the authors state that "neither hair greying nor alopecia were evident" in their mouse model—were there any other physical phenotypes of ageing evident in these mice, as would be expected from a rapidly ageing organism? The authors should include a survival curve of the DNMT3A GOF mouse models, and complement with additional physical and mental fitness tests as previously performed in studies of aging using mouse models (e.g., grip strength, Y-maze test; PMID: 36610399). Additionally, the authors show data of hyperinsulinemia in their DNMT3A GOF mouse model (Fig. 2e, EDF. 2f) – is that because of an underlying insulin resistance in these mice? The authors should complement these observations with a glucose tolerance test during aging in their mouse model.
#2. Many of the conclusions of this manuscript related to DNA hypermethylation during aging are based on the use of DNA methylation arrays (e.g., Fig. 1e). Methylation arrays display an overrepresentation of CpGs within promoters and enhancers, and underrepresentation of CpGs within repetitive elements (which have been reported to influence aging, PMID: 36610399). The authors should explicitly state in the manuscript text the biases and limitations from using DNA methylation arrays towards their major conclusions, and ideally include a new figure which contextualizes the genomic annotation of the identified DMRs and DMVs (i.e., overlapping with enhancers, promoters, etc.) given the bias of CpGs included in the DNA methylation arrays. Additionally, the authors should be explicit in each figure legend about the technology used to measure DNA methylation, as it may be useful to the reader in the interpretation of the results (for example, comparing Fig. 1e and Fig. 4b, notice the detection of 4 times more hypermethylated DMRs with EM-seq compared to DNA methylation arrays).
#3. One of the hallmarks of aging is the decrease of DNA methylation, as indicated in the study from which the authors reanalyzed the DNA methylation patterns in newborn and centenarian individuals (PMID: 22689993). While the authors mostly focus on the DNA hypermethylation happening in Polycomb-marked regions, it is unclear to this reviewer whether there are any DNA hypomethylated regions in the DNMT3A GOF mouse model at all, which may not be detected using a DMR approach (as usually DMR analysis clusters adjacent CpGs with similar methylation changes, and the CpGs that lose methylation during aging tend to be found in CpG-poor regions of the genome). The authors should complement their current analysis of EM-seq data (Fig. 4) with additional genome-wide analysis focusing on DNA methylation changes occurring at

different genomic scales than DMRs, including at the CpG-level as well as the megabase-scale. For analysis at the CpG-level, see an example by the Schübeler lab (PMID: 22170606, Fig. 5b).
#4. In Fig. 5, when the authors focus on DNA methylation changes during human aging using the datasets from PMID: 22689993 (datasets in GSE31263), although the authors refer to those samples as "lymphocytes", the authors should take into account that of those datasets, the 0yr and 103yr samples come from CD4+ T cells, while the 26yr sample may come from PBMCs, which include cells other than lymphocytes. Please check and make the appropriate modifications in the text. Additionally, please make sure to use an appropriate H3K27me3 dataset given this clarification. Currently it is unclear what is the source of the H3K27me3 dataset employed in Fig. 5 (see next comment).
#5. Throughout the manuscript, the authors should indicate explicitly in the figure legend the accession number of the dataset they are using, to avoid any ambiguities. For example, in Fig. 5, it is unclear what is the source of the H3K27me3 dataset being used, and in the Data and materials availability section of the manuscript, the authors are listing as potential source for this dataset an accession number which includes 296 datasets from the ENCODE project across several different cell lines. Furthermore, the authors should aim to cross-compare their datasets to closely related cell types when possible. In EDF. 5c, the authors are presumably using a H3K4me3 dataset from the GM12878 lymphoblastoid cell line, whereas there are datasets in primary T cells publicly available (for example, GSM1586403). Similarly, work by the labs of Ido Amit (PMID: 25103404), Maria Figueroa (PMID: 31085557), Margaret Goodell (PMID: 32386543), and others, have produced several datasets in primary cell types relevant to this manuscript that could be used for reanalysis instead of the ENCODE datasets originating from cell lines that are presumably currently being used.
#6. In lines 151-157, the authors mention that many of the HESJAS patients suffer from lymphopenia, in some cases even leading to severe immunodeficiency, and while it is also said that the mutant mice suffer from low bone marrow cellularity, in the lines 220-221 it is pointed out that these mice do not have reductions of either HSCs or MPPs. Is this also the case in humans for the HESJAS patients? Please comment in the manuscript text.
Minor comments
#1. In Fig. 2f, please add to the figure the cell surface markers used to define "myeloid" and "lymphoid" in the flow cytometry analysis.
#2. In EDF. 1c, please consider replacing the black-scale for some type of color-scale (for example, rainbow), and state which technology was used for this DNA methylation measurement.
#3. In EDF. 1f, please add an explanation about how DMRs were assigned to genes.
#4. In EDF. 4l, please consider adding an additional barplot normalized to 100%, illustrating whether the CFU proportions are the same in DNMT3A GOF cells vs WT, even if the total CFU numbers are decreased in DNMT3A GOF cells.
#5. In lines 276-278, the manuscript mentions that the DNMT3A GOF mice display an enrichment of DNA hypermethylation at "bivalent transcription start sites/promoters marked by H3K4me3 and H3K27me3 (Extended Data Fig. 5a, …)", but the figures referenced should be EDF. 5b-c.

Reviewer #3 (Remarks to the Author):

In this manuscript, the authors explore age-associated DNA hypermethylation and its potential role in causing ageing-related phenotypes. Drawing on both a cohort of individuals with Heyn-Sproul-Jackson syndrome (HESJAS), caused by gain-of-functon(GOF) mutations in DNMT3A, and a Dnmt3aW326R/+ mouse model, they observe age-associated phenotypes in both human and mice. Aiming to investigate the cellular basis of these phenotypes, they show stem cell dysfunction in adult Dnmt3aW326R/+ mice. Furthermore, accumulation of DNA hypermethylation at Polycomb-marked regions are observed. This manuscript addresses an important and unresolved question in the field of epigenetic ageing. The authors establish an interesting monogenic ageing model, and integrate functional stem cell assays, methylome and transcriptomic profiling, and clinical phenotyping to argue that DNA hypermethylation underlies stem cell exhaustion and downstream tissue pathology. However, while the study is ambitious, there are several concerns that dampened my initial enthusiasm after reading the abstract.

Comments:
1. My major concern is that this study seems to be a follow up on the previous work published on HESJAS by the same group. While this is appreciated and of course relevant for understanding HESJAS, the links to age-related changes in DNA methylation are correlative, and the direct interpretation of how this disease relates to tissue ageing is unclear.

2. Along the same lines, there is little mechanistic insight on how the HESJAS-specific PWWP mutation can be used as a model to study age related changes in methylation that occur in presence of wt DNMTs. Why does impaired binding to H3K36me mimic methylation changes observed in aged tissues?

3. The authors' model suggests that methylation is the key variable, but alternative epigenetic or genetic effects of the mutation are not discussed. The observed phenotypes may be the downstream result of broader molecular disruptions caused by the PWWP domain mutations. E.g., could the mutation in the PWWP domain of DNMT3A disrupt protein complexes at target genes required for differentiation independently of its methylation activity? The authors should temper causal claims and consider that hypermethylation might be a readout of dysregulation rather than the initiating cause.

4. The scRNA-seq data show modest transcriptional changes between wild-type and Dnmt3aW326R/+ progenitor cells, despite pervasive hypermethylation at Polycomb-marked regions. This raises the question about the functional impact of the methylation changes. The authors propose that transcriptional differences emerge during differentiation, due to impaired induction of lineage genes. This is plausible, but the modest expression changes weaken the case for direct repression of gene expression via DNA methylation. An alternative explanation could be that delayed differentiation is due to cell cycle

perturbation or altered proliferation dynamics rather than slowed transcriptional activation. A clearer explanation of why transcriptional changes are so limited and how this supports the proposed mechanism are necessary.

5. The claim that lineage-defining genes show reduced expression across pseudotime in mutant cells is interesting but required additional clarification. It is unclear whether this observation reflects a reduction in cell numbers progressing to late stages or a true defect in transcriptional activation of target genes.

6. Although the manuscript states that hypermethylation affects Polycomb domains and bivalent promoters, the gene-level analysis of methylation changes remains limited. A more systematic intersection between DMRs and curated lists of lineage-defining genes is missing.

Version 1:

Decision Letter:

Our ref: NG-A68576R

4th Feb 2026

Dear Dr. Jackson,

Thank you for submitting your revised manuscript "An Epigenetic Progeria Links Hypermethylation to Age-Related Pathology" (NG-A68576R). It has now been seen by the original referees and their comments are below. The reviewers find that the paper has improved in revision, and therefore we'll be happy in principle to publish it in Nature Genetics, pending minor revisions to satisfy the referees' final requests and to comply with our editorial and formatting guidelines.

If the current version of your manuscript is in a PDF format, please email us a copy of the file in an editable format (Microsoft Word or LaTex)-- we can not proceed with PDFs at this stage.

We are now performing detailed checks on your paper and will send you a checklist detailing our editorial and formatting requirements soon. Please do not upload the final materials and make any revisions until you receive this additional information from us.

Thank you again for your interest in Nature Genetics Please do not hesitate to contact me if you have any questions.

Sincerely,

Margot Brandt, PhD
Senior Editor
Nature Genetics
https://orcid.org/0000-0002-9434-794X

Reviewer #1 (Remarks to the Author):

The authors have successfully addressed my concerns and I have no further comments.

I enjoyed reading your paper and I look forward to seeing more of your work.

Reviewer #2 (Remarks to the Author):

The authors have addressed all our comments

Reviewer #3 (Remarks to the Author):

The authors have addressed most of my comments and added very good additional experiments to this new version.

I find it very exciting that HESJAS shows a novel form of monogenic progeria driven by gain of function of DNMT3A. Therefore, I fully support the author's conclusions that this mutation in DNMT3A causes accelerated hypermethylation at Polycomb promoters and leads to premature ageing, which makes these findings relevant for broad audience.

That said, I still do not agree with the claims of causality laid out in the current version. The authors indicate that the measured hypermethylation of Pax5 in Dnmt3aW326R/+ pre-pro-B cells is the reason for the failed differentiation to pro-B cells (new Fig 5). While indeed Dnmt3aW326R/+ inhibits this differentiation, and indeed there is hypermethylation observed in these cells, there is no experiment to support that DNA methylation at the Pax5 promoter directly influences this outcome (or transcription of Pax5).

While the results in the current form suggest correlations, the authors write:
L103: “we causally implicate DNA methylation-mediated stem cell exhaustion in the aetiology”
L150: “methylation at the promoter of a key transcription factor, Pax5, is the likely cause of reduced cellular output in this lineage to account for lymphopenia one of the age-related pathologies observed. Together, these findings provide experimental evidence supporting a causal link between hypermethylation and physiological ageing.”
L395: “DNA hypermethylation of a key lineage transcription factor in adult stem cells leads to impaired differentiation, promoting an age-related phenotype”
L485: “demonstrate that DNA hypermethylation slows transcriptional activation dynamics during differentiation” … “implicates such hypermethylation as the cause, at least of this pathology. ”

If the authors want to show a causal relationship, they need to show that indeed the presence of methylation is leading to transcriptional repression of Pax5 and subsequently differentiation block with functional/mechanistic assays. And/or identify and test a mechanism that could link methylation to repression, i.e. blocked transcription factor binding, recruitment of MBD proteins. I agree that such would be too much asked for this manuscript, which is already extensive, but without such mechanistic insights, the authors should rephrase and exchange “causality” with “association”.

Open Access This Peer Review File is licensed under a Creative Commons Attribution 4.0 International License, which permits use, sharing, adaptation, distribution and reproduction in any medium or format, as long as you give appropriate credit to the original author(s) and the source, provide a link to the Creative Commons license, and indicate if changes were made.
In cases where reviewers are anonymous, credit should be given to 'Anonymous Referee' and the source.
The images or other third party material in this Peer Review File are included in the article’s Creative Commons license, unless indicated otherwise in a credit line to the material. If material is not included in the article’s Creative Commons license and your intended use is not permitted by statutory regulation or exceeds the permitted use, you will need to obtain permission directly from the copyright holder.
To view a copy of this license, visit https://creativecommons.org/licenses/by/4.0/

Rebuttal NG-A68576, Neary et al.,

Reviewers' Comments:

We thank all reviewers for recognising the significance of our findings and for their insightful criticisms. These have help us to significantly improve the manuscript during the 6-month revision time-frame.

Key issues raised were:

a. An organism-level description of ageing phenotypes and survival curve for the HESJAS mouse was needed (Reviewers 1 and 2);
b. Exclusion of unrecognised DNA hypomethylation changes, and biases arising from using DNA methylation arrays (Reviewer 2);
c. Insufficient causal evidence for a direct role for hypermethylation in tissue pathology (Reviewer 3).

Responding to these key issues, we have:

a. Substantially extended phenotyping of our mouse model, discovering additional age-related phenotypes (Reviewer response 2.1) and demonstrated significantly reduced survival (Reviewer response 1.1);
b. Performed additional analyses at single CpG site, 10kbp, 100kbp and 1Mbp resolution, which did not detect any age-associated DNA hypomethylation. In particular, alterations at PMDs ('partially methylated domains') were not identified. Potential biases from use of DNA methylation arrays were also addressed, and we confirm that the W326R mutation results specifically in hypermethylation at Polycomb-marked regions (Reviewer response 2.2);
c. Demonstrated that gain of hypermethylation at the promoter region of a lineage-specific gene cause substantial reduction in transcription and result in reduced cell output by impairing the key differentiation transitions regulated by this gene (Fig. 5 of the revised manuscript). We show (i) hypermethylation of Pax5, a transcription factor and master regulator of early B lymphocyte development. (ii) The impact on its transcription at Hardy stage B and C, for which it is critical and (iii) the consequent marked reduction in cells at later stage lymphocyte development. Furthermore, B cells surviving this transition are under strong selection against hypermethylation at the Pax5 locus (for further data and discussion, see Reviewer response 3.4).

Below, we comprehensively address all reviewers' questions on a point-by-point basis. (Please note: for the reviewers' convenience, all citations in the rebuttal (including those that quote sections from the revised manuscript) are numbered in the order they appear, with a reference list provided at the end.) Taken together, these strengthen our original conclusion that hypermethylation causes specific age-related pathology. Nevertheless, we have taken on board counsel from Reviewers 2 and 3, and tempered causal claims, by revising title, abstract and text to ensure we are clear on the conclusions that can be drawn from this study. The proposed revised title is:

"*An epigenetic progeria links DNA hypermethylation to age-related pathology*".

Figure 5

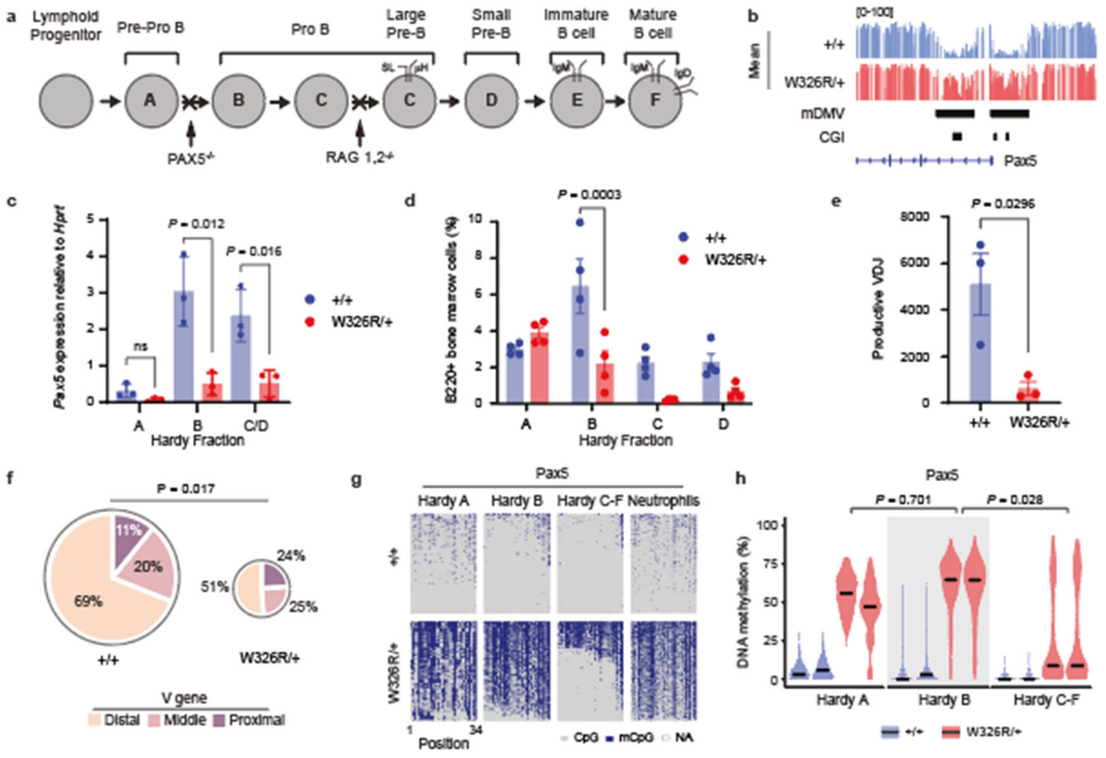

Figure 5. HESJAS B cell differentiation is delayed at the same stage as $Pax5^{-/-}$ bone marrow. a, Schematic of B cell differentiation stages (adapted from [93,94]). **b,** DMV hypermethylation at the 5' end and promoter region of *Pax5* in $Dnmt3a^{W326R/+}$ HSCs. Genome browser view, mean DNA methylation from n = 9 mice per genotype. chr4:44,685,675-44,729,761; EM-Seq. **c,** RT-qPCR analysis demonstrates reduced Pax5 mRNA expression levels in B-cell Hardy fractions B and C/D in 3-month old $Dnmt3a^{W326R/+}$ mice. Mean ± SD, unpaired t-tests. **d,** B-cell numbers are reduced from Hardy B stage onwards. Data points, 2-3 month old mice, mean ± SD, t-tests. **e,** Reduced productive VDJ recombination events. Data points, 2-3 month old mice, mean ± SD, t-tests. **f,** VDJ recombination is bias towards proximal V gene usage, chi-squared test. Percentage VDJ recombination events with proximal, middle and distal V genes. **g,h,** *Pax5* promoter hypermethylation is reduced at later stages of B cell differentiation. Targeted EM-PCR analysis of DNA methylation of a 562bp amplicon at the *Pax5* promoter region. **g,** Representative CpG methylation state matrices of sorted cell populations from individual mice, ages 2-3 months. Blue, mCpG; grey, CpG. Columns, individual CpGs numbered from start of amplification. Rows, individual sequencing reads. (Panels reproduced in EDF9 alongside replicate data at higher magnification.) **h,** Violin plot. Percentage DNA methylation of individual amplicons. Centre line, median. *P* value, two-sided t-test between population means for n=2 mice.

Reviewer #1 (Remarks to the Author):

In this work, the authors show that patients with DNMT3A gain-of-function mutations as well as a mouse model exhibit features of accelerated ageing. They also show that region-specific DNA hypermethylation leads to stem cell dysfunction, suggesting that stem cell exhaustion drives the observed ageing phenotypes. This is a fascinating, original and timely work that provides causal evidence for a role of DNA methylation in ageing. I only have a few comments and suggestions.

Thank you for highlighting the importance and originality of our work for the ageing field.

My major issue is that from an ageing perspective some additional detail/information would be valuable. Regarding the HESJAS patients, my understanding from figure 1A is that these are relatively young patients. I would be interested in knowing the age range of these

patients, also whether there is any indication of premature death - from what I could gather on page 5 only one patient has died so far, is that correct?

The reviewer was correct that only one of the patients had died; however, another sadly passed away in the last week. Age data for all patient is provided in Supplementary Data Table 2. Average lifespan for human HESJAS at this point is unknown as ascertainment is heavily biased to early childhood, and currently without the benefit of extended longitudinal data. Our human subjects have a median age of 10.75 years with an age range of 4-63 years. However, older patients have milder phenotypes that likely reflect weaker mutation severity, adding complexity to determining expected lifespan for HESJAS.

Likewise, I wonder what is the lifespan of the mutant mice or, at least, if there is evidence of premature mortality?

Premature mortality is evident in our mouse model. We now provide a survival curve as part of Fig. 2, which demonstrates that lifespan is clearly shortened, with a median survival of 12.8 months compared with an expected life expectancy of wild-type C57Bl/6J mice reported to be 26-29 months[3] . This therefore supports the notion that this is an accelerated ageing syndrome.

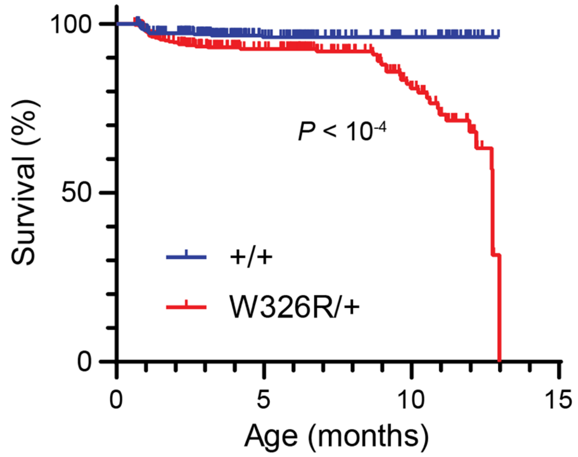

If so, what do the animals die of?

Increasing frailty was observed between 6 and 12 months of age (Reviewer response 2.1 documents the progressive multi-system phenotypes with advancing age). Mortality was either spontaneous (found dead in cage) or as a result of humane endpoints when mice reached moderate severity, with criteria defined by the UK Home Office used by their carers for twice daily observations/scoring. These criteria are a measure of poor overall health and include changes in appearance (e.g. coat staring, hunched posture, hair loss), behaviour (e.g. change in temperament, reluctance to move) and other clinical signs (e.g. reduction in body weight, diarrhoea, abnormal breathing). Wildtype littermates were similarly monitored from 6 months of age and terminated when moderate severity was reached.

We address these points textually:

"We identified 10 individuals (median age 11) with $DNMT3A^{GOF}$ mutations."

"Dnmt3a$^{W326R/+}$ mice from 6 months exhibited increasing frailty. Survival was significantly reduced compared to wild-type littermates (Fig. 2a), with a median life expectancy of 12.8 months, less than half of the expected 26-29 months reported for C57BL/6J animals[3]. Dnmt3a$^{W326R/+}$ mice exhibited postnatal growth failure (Extended Data Fig. 2a) and slow but gradual weight loss observed from 6 months of age (Extended Data Fig. 2b) until end of life, generally between 9 months and 1 year. At end point, cataracts, reduced coat condition and kyphosis were also evident (Extended Data Fig. 2c-e)."

And in the Methods section:

"Survival data was collected from our colony throughout the period of this study. Events were recorded for spontaneous deaths (found dead in cage) and upon termination as a result of humane endpoints when mice reached moderate severity, with criteria defined by the UK Home Office used by their carers for twice daily observations/scoring. These criteria are a measure of poor overall health and include changes in appearance (e.g. coat staring, hunched posture, hair loss), behaviour (e.g. change in temperament, reluctance to move) and other clinical signs (e.g. reduction in body weight, diarrhoea, abnormal breathing). Wildtype littermates were similarly monitored from 6 months of age and terminated when moderate severity was reached. Any mice terminated for other reasons were censored from time of termination"

Although there is a mention to epigenetic clocks (page 8), it was not clear to me whether epigenetic clocks are accelerated in HESJAS patients. And what about epigenetic clocks in the mouse model?

Epigenetic clock age is indeed accelerated in both human HESJAS patients and in the mouse model. This analysis is now provided in Extended Data Fig. 10e,g.

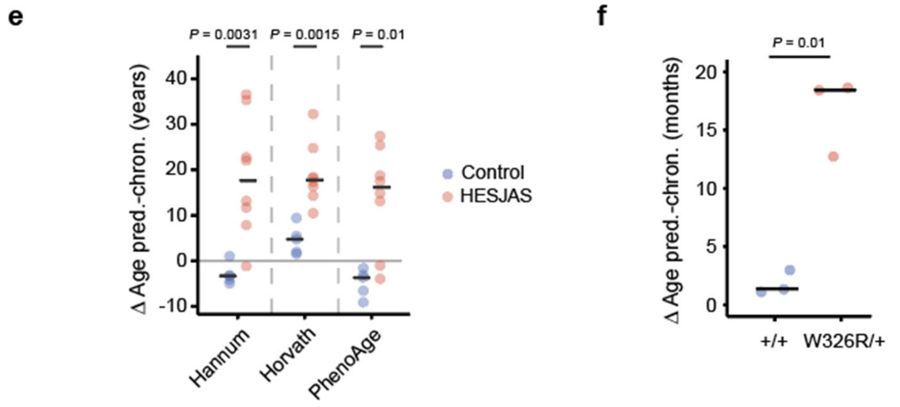

We state in the results:
"*Also, a DNA methylation clock derived from HESJAS hyper-DMR CpGs, performed comparably to several published clocks[4-7] in the GS dataset in predicting chronological age (Fig. 6d,e). In addition, existing clocks predicted accelerated age in both HESJAS patients and the mouse model[4-8] (Extended Data Fig. 10e,f)."*

Lastly, given that wild type Dnmt3a hinders stem cell renewal, I wonder what is its evolutionary purpose?

Previous studies established that after serial transplantations, *Dnmt3a*$^{-/-}$ haematopoietic stem cells were more likely to self-renew than differentiate[9,10]. So, in this sense DNMT3A indeed seems to hinder stem cell renewal. However, our interpretation would be rather that DNMT3A has a regulatory role in deciding whether a stem cell self-renews or differentiates. In an evolutionary context, if DNMT3A activity varies between species, the balance between such renewal and differentiation could contribute to available regenerative capacity during ageing. Recent work by Chandra and colleagues is intriguing, as they demonstrate a significant correlation between DNA methylation rate and longevity between species[11].

In other words, what is the downside of Dnmt3a? I appreciate this a more evolutionary perspective outside the scope of the work, but I would be interested in the authors thoughts on this.

The potential downside for DNMT3A is its affinity for Polycomb-marked histones. While this likely limits expansion of Polycomb-domains, this seems to then have detrimental consequence, permitting wild-type DNMT3A to gradually replace Polycomb marks with DNA methylation during physiological ageing.

To respond, we now provide additional discussion on the role of wild-type DNMT3A in age-related DNA methylation and potential evolutionary relevance:

"The multilineage impact on stem cell output, combined with the accelerated nature of hypermethylation in HESJAS can account both for its impact on growth during development and later effects on tissue regenerative capacity in adult life. In physiological ageing, gradual DNA hypermethylation by wild-type DNMT3A in the population at large may be accounted for by the affinity of the DNMT3A1 N-terminal region for H2AK119Ub[12,13], an epigenetic mark enriched in Polycomb regions (see model, Extended Data Fig. 10g). Such cumulative hypermethylation would consequently be predicted to contribute to similar tissue pathologies as those seen in HESJAS, but in later life. Notably, differing DNA methylation rates between species correlate with longevity[11], raising the possibility that altered DNMT3A activity could be a contributing determinant to mammalian lifespan."

Overall, I think this is a fascinating work that will make an important contribution to the field, though a few aspects could be improved/clarified.

Thank you.

It is possible that some of my comments reflect misunderstandings of mine. If so then I would suggest that the authors use my misunderstandings as an indication that such points might be made clearer in the manuscript.

It is my usual policy to reveal my identity to the authors: Joao Pedro de Magalhaes.

Reviewer #2 (Remarks to the Author):

NG-A68576 ms review, Neary et al., DNA hypermethylation is causal for multiple age-related pathologies

Description

In this manuscript, Neary et al. utilize a DNMT3A gain-of-functon(GOF) mutant mouse model to study the role of aberrant DNA hypermethylation during aging. The DNMT3A GOF mutation of interest (W326R in mouse DNMT3A) is found within the PWWP domain, and results in relocalization of DNMT3A away from H3K36me2/3-marked regions into Polycomb-marked regions, with a corresponding gain of DNA methylation in such regions. This DNMT3A GOF mutation is similar to the ones observed in patients with Heyn-Sproul-Jackson syndrome (HESJAS), which is characterized by global developmental delay. Since the DNMT3A GOF mouse model displays DNA hypermethylation at Polycomb-marked regions in several tissues, as has been previously reported to occur during aging in somatic mitotic cells (PMID: 39009581), the authors propose that this mouse model would allow them to establish a causality between DNA hypermethylation at Polycomb-marked regions and ageing.

However, the data currently presented in this manuscript is insufficient to support such a statement. The manuscript is largely missing measurements of aging at the organismal level in the DNMT3A GOF mouse model. Moreover, several of the authors' conclusion regarding DNA methylation changes are based on analysis of a limited subset of CpGs mostly overlapping with promoters and enhancers (obtained using Infinium Methylation Arrays), and their analysis of whole-genome methylation data is similarly biased towards those regions. Additional experiments and considerations are needed to clarify the mechanism proposed in the title ("DNA hypermethylation is causal for multiple age-related pathologies"). Alternatively, the title of this manuscript should be revised to better reflect the conclusions that can reasonably be drawn from the presented data.

We thank the reviewer for highlighting these important issues, which we address below.

Major comments

#1. Although the manuscript aims to present a mouse model of aging, there is a lack of measurements of aging at the organism level in the DNMT3A GOF mouse model. For example, while Fig. 2a suggests that the DNMT3A GOF mouse model displays a similar developmental delay during the first 9 wk of life as is observed in HESJAS patients, in lines 195-196 of the manuscript, the authors state that "neither hair greying nor alopecia were evident" in their mouse model—were there any other physical phenotypes of ageing evident in these mice, as would be expected from a rapidly ageing organism?

Yes, there are additional physical age-related phenotypes that we now document in Fig. 2 and Extended Data Fig. 2 and 3 of the revised manuscript. After 6 months of age, kyphosis (Extended Data Fig. 2d), reduced subcutaneous fat (Extended Data Fig. 2m,n), cataracts (Extended Data Fig. 2e) and a gradual decrease body weight become evident (Extended Data Fig. 2b). This is in addition to the previously documented phenotypes that were apparent from 3-6 months: haematological (myeloid skewing; Fig. 2f and 3g), bone health

(osteoporosis; Fig. 2b-c, Extended Data Fig. 3a-c), metabolic (lipodystrophy, hyperinsulinemia, Fig. 2e, Extended Data Fig. 3d-i) and skin (reduced hair follicle length and epidermal thickness, Extended Data Fig. 2j-l). As with other monogenic progeria syndromes, a subset of ageing pathologies are seen. This is consistent with each of these disorders reflecting a specific aetiological aspect of the multifactorial process of ageing. In the case of HESJAS, this informs on a component of the 'epigenetic alterations' hallmark process, specifically hypermethylation.

The authors should include a survival curve of the DNMT3A GOF mouse models,

As requested, we now provide a survival curve (Fig. 2a; also see our response to Reviewer 1). This demonstrates a substantial and significant reduction in lifespan of Dnmt3a GOF mice compared with isogenic C57BL/6J wildtype littermates.

and complement with additional physical and mental fitness tests as previously performed in studies of aging using mouse models (e.g., grip strength, Y-maze test; PMID: 36610399).

We have performed further physical and behavioural testing that together demonstrate progressive frailty across multiple systems from 6 months of age. These contribute to the premature mortality documented above. In addition to the physical changes noted above, neurocognitive assessment demonstrated the mice to have reduced nocturnal activity (Extended Data Fig. 2f), the time of day that rodents are normally most active. In open field tests they also spend less time in the field centre (positive thigmotaxis) consistent with increased anxiety (Extended Data Fig.2 g-i), a phenotype previously reported in mouse Alzheimer models[14,15].

Not all ageing phenotypes are however evident in our mice, as expected for a monogenic progeria syndrome. This informs us which tissue pathologies are not associated with age-related DNA hypermethylation. For instance, grip strength and Y-maze alternations were not altered (Extended Data Fig. 3k-m), in contrast to PMID 36610399[16] as referenced by the reviewer. This work reports ERV reactivation as a driver for aging-associated articular degeneration. In our HESJAS model, there was no histological evidence of osteoarthritis at 10-12-month of age (Extended Data Fig. 3n,o). Whether DNA hypermethylation contributes to hair greying in humans is less clear, as we now understand that that hair greying is not well modelled in mice, and indeed is not evident in other mouse progeria models (Reviewer figure 1). We do note that our Dnmt3a mutant mice show lightening of their fur with age, a phenotype that may be interesting to investigate further in future.

We now provide the following text in the revised manuscript, in response to the reviewer's comments:

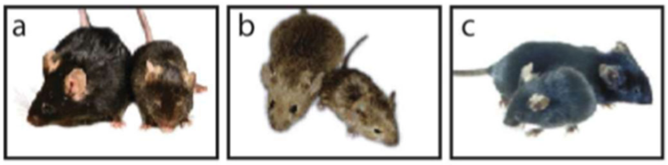

Reviewer Fig. 1| Progeria syndrome mice are small and do not display premature greying. **a,** HESJAS; **b,** Hutchinson-Guilford Progeria syndrome and **c,** ERCC1progeric mice next to wild-type littermates. Images for **b** and **c** reproduced from refs[1,2].

"Dnmt3a$^{W326R/+}$ mice from 6 months exhibited increasing frailty. Survival was significantly reduced compared to wild-type littermates (Fig. 2a), with a median life expectancy of 12.8 months, less than half of the expected 26-29 months reported for C57BL/6J animals[3]. Dnmt3a$^{W326R/+}$ mice exhibited postnatal growth failure (Extended Data Fig. 2a) and slow but gradual weight loss observed from 6 months of age (Extended Data Fig. 2b) until end of life, generally between 9 months and 1 year. At end point, cataracts, reduced coat condition and kyphosis were also evident (Extended Data Fig. 2c-e). Neurobehaviourally, reduced nocturnal activity was seen at 6 months and positive thigmotaxis was noted on open field testing at 1 year (Extended Data Fig. 2f-i). In 6-12 month old mice, we also observed decreased hair follicle length, reduced epidermal thickness and loss of subcutaneous fat (Extended Data Fig. 2j-n)."

"As with other monogenic progeria syndromes, not all features of ageing are present in HESJAS. For instance, there was no clear evidence of osteoarthritis, hair greying, reduction in grip strength or decrease in performance with Y-maze testing in the Dnmt3a$^{W326R/+}$ mouse (Extended Data Fig. 2c,d; Extended Data Fig. 3k-o)."

Additional organism level data provided:

Extended Data Figure 2

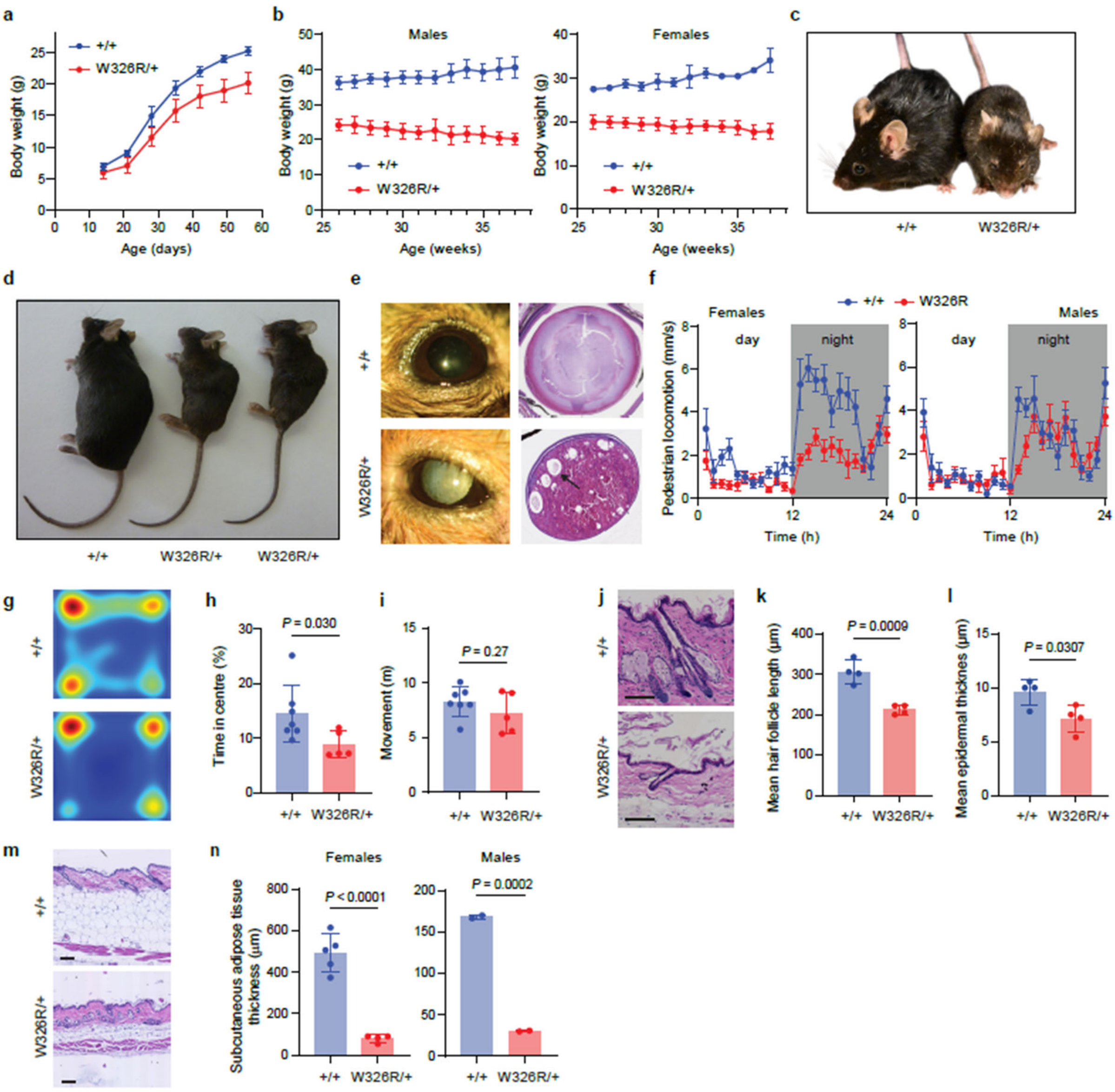

Extended Data Fig. 2| Dnmt3a$^{W326R/+}$ mice model HESJAS syndrome and exhibit premature ageing. a, Body weight of Dnmt3a$^{W326R/+}$ mice is significantly decreased post-weaning. Weight by age, mean ± 95% confidence intervals, *n* = 8 wildtype (+/+), *n* = 11 mutant (W326R/+) male C57BL/6J mice. *P* = 0.0006 by genotype, 2-way ANOVA. **b**, After 6 months W326R/+ mice of both sexes show a gradual decline in body weight. Males (*n* = 7 +/+, *n* = 6 W326R/+), females (*n* = 3 +/+, *n* = 5 W326R/+). **c-e**, Features of ageing at one year of age in the HESJAS mouse model. **c**, 1-year-old W326R/+ mouse next to C57BL/6J wildtype littermate. Features of premature ageing: **d**, kyphosis; **e**, senile cataracts with vacuoles and disorganisation of fibres in the lense nucleus; **f-i**, neurocognitive deficits. **f**, reduced nocturnal activity (at 6 months); **g-i**, open field avoidance (positive thigmotaxis), **g**, density plot of open field position, **h**, time in centre of field, **i**, total movement over 20 minutes (*n* = 7 +/+, *n* = 5 W326R/+ male mice). **j-l**, Reduced hair follicle length and epidermal thickness in W326R/+ mice. **j**, Representative images and quantification of (**k**) mean hair follicle length and (**l**) epidermal thickness. Data points, individual 6-month-old male mice (*n* = 4 +/+ and *n* = 4 W326R/+). **m,n**, Reduced subcutaneous adipose tissue in old W326R/+ mice. **m**, Representative images and **n**, quantification (data points, individual 1-year-old females (*n* = 5 +/+, n = 4 W326R/+) and 13-month old males (*n* = 2 +/+, n = 2 W326R/+). For **a,b,h,k,l,n**, Mean ± s.d., unpaired t-test. For **i**, Mean ± s.d., Mann-Whitney test. For **j,m**, size bars, 100 µm.

And Extended Data Figure 3n,o,k-q

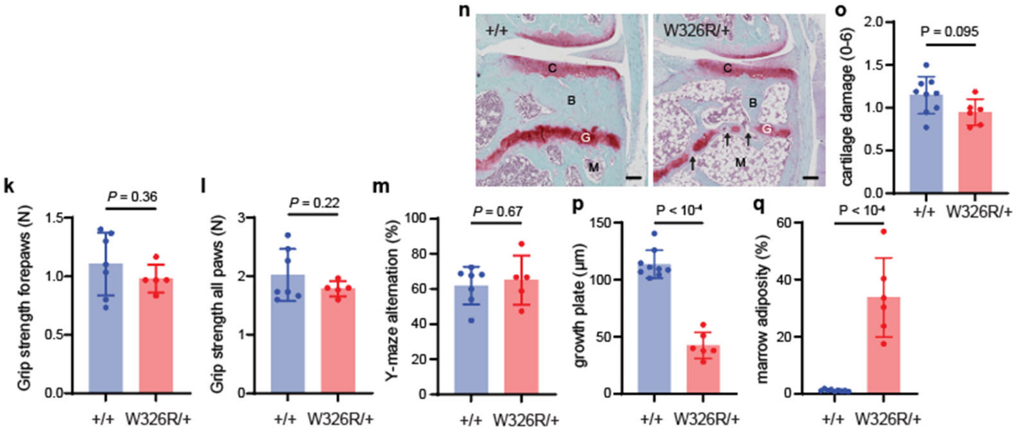

Extended Data Fig. 3| Dnmt3a$^{W326R/+}$ gain of function mutation leads to features of segmental progeria in mice. k,l, No significant change in grip strength or **m,** Y-maze alternations. **n-q,** No osteoarthritis, but reduced bone/growth plate and increased bone marrow adiposity in 10-12 month-old W326R/+ mice. **n,** Representative images of the medial tibiofemoral articulation in the knee stained with Safranin O (red, cartilage) and Fast Green (green, bone), showing partially closed growth plate (G, arrows), decreased bone (B) and increased adiposity in marrow (M), while articular cartilage (C) appears normal. **o,** No increased articular cartilage damage characteristic of osteoarthritis, assessed by OARSI scoring (scale 0-6), average score of medial and lateral tibial plateau and femoral condyles. *P*-value, Mann-Whitney U test. **p,** Reduced thickness of proximal tibial growth plate. **q,** Increased adiposity, % distal femoral metaphyseal bone marrow space occupied by adipocytes. *P*-value, unpaired t-test after log transformation. Data points, individual 10-12 month old males (*n* = 9 +/+, *n* = 6 W326R/+). For **a-m**, **o-p**, Mean ± s.d., unpaired t-tests. For **d**, **e**, **i** and **n** scale bars, 100 µm.

Additionally, the authors show data of hyperinsulinemia in their DNMT3A GOF mouse model (Fig. 2e, EDF. 2f) – is that because of an underlying insulin resistance in these mice? The authors should complement these observations with a glucose tolerance test during aging in their mouse model.

Yes, insulin resistance is present in this mouse model. At 6 months old, blood insulin concentrations are around 3-fold higher in both male and female mutant mice compared to their WT littermates. Because glucose concentrations are unchanged, this is unequivocal evidence of insulin resistance. To make this clearer we now present insulin and glucose data side-by-side (Fig. 2e and Extended Data Fig. 3g). Insulin resistance can be quantified as a HOMA-IR (Homeostatic Model Assessment for Insulin Resistance) score, which takes into account both insulin and glucose[17], and this is markedly increased in Dnmt3a GOF mice. Additional testing in younger animals demonstrates that insulin resistance begins to emerge at 3 months, with significant insulin resistance evident in males at this stage (Extended Data Fig. 3h,i). The finding of insulin resistance on a normal chow diet is remarkable, as even many murine models of monogenic insulin resistance do not manifest insulin resistance on chow alone[18].

Insulin resistance in our mouse model manifests alongside ectopic lipid accumulation in the liver and brown fat. This reflects "overspill" from white adipose tissue with impaired storage capacity, and low serum levels of the adipokine adiponectin. This "adipose failure" mirrors metabolic syndrome associated with ageing and obesity in humans, rather than pure primary insulin resistance due to insulin signalling defects, which has a distinct metabolic phenotype that does not include ectopic lipid deposition or low adiponectin (e.g. refs[19-21]).

We have performed glucose tolerance testing (GTT) as requested by the reviewer. Glucose tolerance is also already impaired at 3 months of age, significantly so in males. However, this finding is of limited additional value, given that GTT is often a poor test of insulin resistance in mice as in this species most glucose clearance is insulin independent[22].

Below, we present GTT results for the reviewer and include HomA-IR scores in Extended Data Fig. 3 of the revised manuscript.

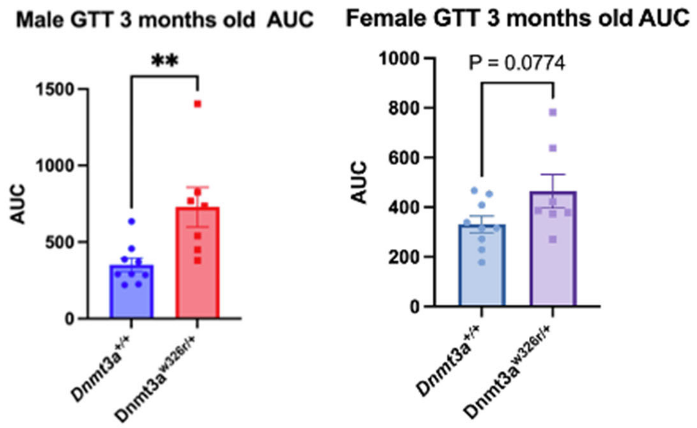

Reviewer Fig. 2| Glucose tolerance test. Data points, n=9 Dnmt3a^{++} , n=7 Dnmt3a$^{W326R/+}$ mice fasted for 4 h and gavaged with glucose (2 g/kg body weight). Mean area under the curve (AUC) ± s.e.m. t-test. (AUC is presented, as the most rigorous comparator of GTT, see ref[22])

Responding to the reviewer's request for clarification, we have revised the manuscript:

"Metabolic assessment demonstrated three-fold higher plasma insulin in 6-month-old *Dnmt3a$^{W326R/+}$ mice compared to wildtype littermate controls, despite no difference in blood glucose, indicating marked insulin resistance (Fig. 2e, Extended Data Fig. 3g). This was evident already at 3 months of age as measured by increased HOMA-IR score[17], (Homeostatic Model Assessment for Insulin Resistance; Extended Data Figure 3h,i). Reduced serum concentrations of adiponectin, a marker of adipose health, together with increased lipid content of liver (hepatic steatosis) and brown adipose tissue (Fig. 2d,e; Extended Data Fig. 2d-f) suggest functional adipose failure as the likely cause of insulin resistance. These features were seen in mice fed a normal chow diet, which is striking given that many mouse models of insulin resistance require challenge with a high fat diet to induce a metabolic phenotype[18]. Metabolic phenotypes appeared to be progressive in both males and females."*

Fig. 2e

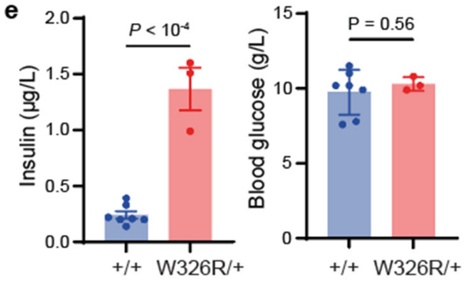

Extended Data Fig. 3h,i

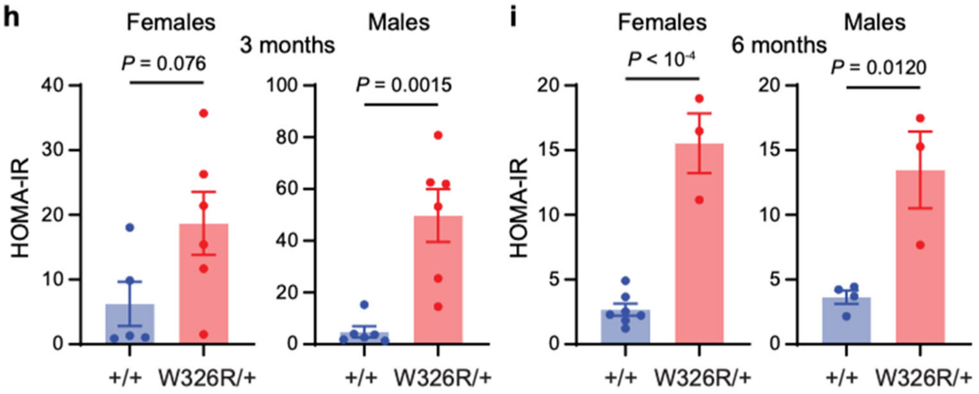

#2. Many of the conclusions of this manuscript related to DNA hypermethylation during aging are based on the use of DNA methylation arrays (e.g., Fig. 1e). Methylation arrays display an overrepresentation of CpGs within promoters and enhancers, and underrepresentation of CpGs within repetitive elements (which have been reported to influence aging, PMID: 36610399).

We thank the reviewer for highlighting the potential biases from selective CpG representation in DNA methylation arrays. We address this as follows:

Firstly, findings from DNA methylation arrays are all concordant with our EM-Seq analyses which examines CpG methylation at single-nucleotide resolution across the whole genome.

Secondly, to quantify the degree of representation of different genomic contexts, we have generated heat matrices of ChromHMM states to quantify the number of CpGs present in each category for Human Infinium Methylation EPIC BeadChip and Infinium Mouse Methylation BeadChip array (respectively, Extended Data Fig. 1e and Extended Data Fig. 4m,n). This demonstrates that while general enhancers and transcribed regions are highly represented on the arrays (categories 1-7), Polycomb associated features are relatively under-represented (categories 10-14), hence detected enrichment is not the consequence of over-representation on DNA methylation arrays.

Thirdly, all enrichment analyses we present in our study are corrected for this background CpG number on the arrays for each individual category. Therefore, the significant enrichment of Polycomb marked regions is not due to array representation bias.

With regard to repetitive elements, these are primarily present in ChromHMM categories 8_Het (Heterochromatin) and 15_Quies (Quiescent/Low activity), neither of which are enriched in our analysis.

Extended Data Fig. 1e

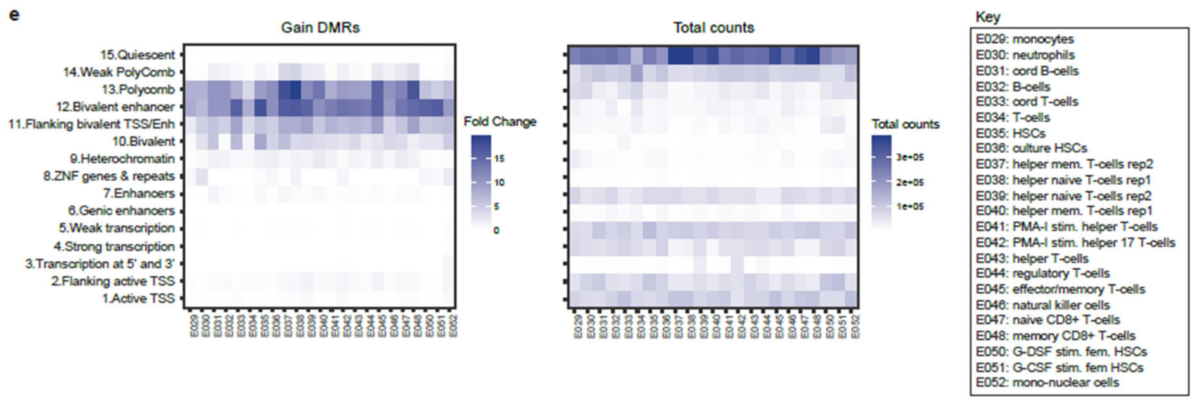

Extended Data Fig. 4m,n

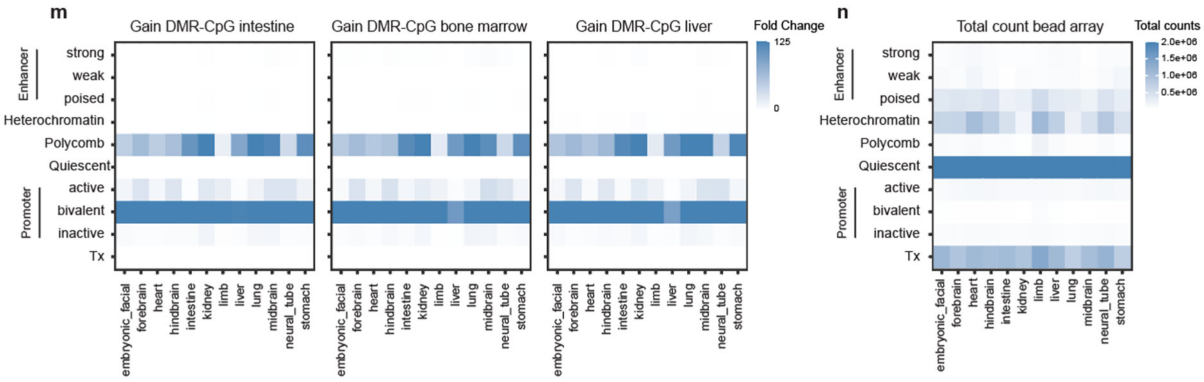

The authors should explicitly state in the manuscript text the biases and limitations from using DNA methylation arrays towards their major conclusions, and ideally include a new figure which contextualizes the genomic annotation of the identified DMRs and DMVs (i.e., overlapping with enhancers, promoters, etc.) given the bias of CpGs included in the DNA methylation arrays.

As requested, we state in the manuscript the biases and limitations of DNA methylation arrays. We also provide as requested additional analysis intersecting DMRs with genomic annotations as (Extended Data Fig. 1f and Extended Data Fig. 4o). These again demonstrate significant enrichment at promoters and adjacent regulatory regions, above the representation on CpG methylation bead arrays.

Extended Data Fig. 1f

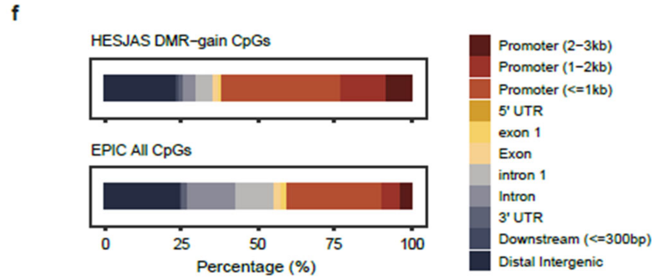

Extended Data Fig. 4o

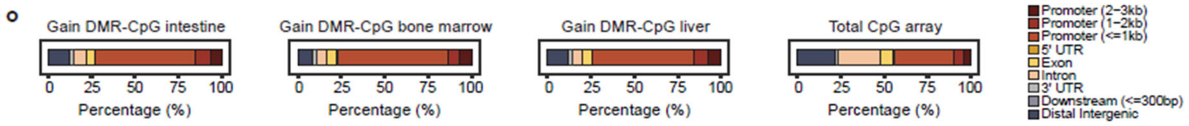

As well, to further address the specific question raised by the reviewer as to whether repetitive elements had altered DNA methylation, we have performed additional analyses using UCSC genomic RepeatMasker annotations for interspersed repeats (includes retrotranscripts (SINES), DNA transposons, retrovirus retrotransposons, non-retrovirus retrotransposons (LINES)). Analysis shows that no significant enrichment of either gain or loss HSC-DMR CpGs in repetitive elements, but rather a significant depletion, indicating DMRs are mainly excluded from repetitive elements (Reviewer Fig. 3). Likewise, this is the case for single site CpGs identified in the DMR-independent analysis performed for Reviewer 2 point 3).

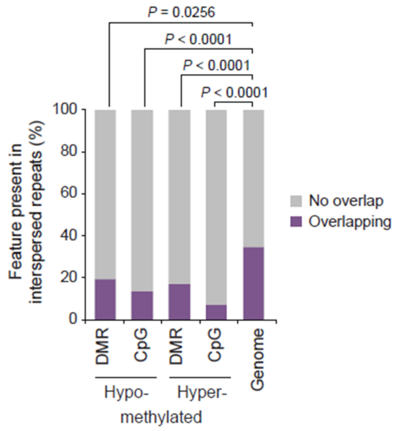

Reviewer Fig. 3| Hypo- and hyper-methylated DMRs and individual CpGs are depleted for interspersed repeats. Percentage of hypo- and hyper-methylated DMRs or single-site-CpGs (Extended Data Fig. 6g; EM-Seq for HSCs) that overlap with interspersed repeats (UCSC genome annotation, Repeatmasker) compared with frequency of genome-wide occurence of CpGs in interspersed repeats. Two-sided Fisher's exact test.

Addressing these issues in the manuscript text, we now state in the results:

"Enrichment of Polycomb-marked regions was also evident in bone marrow and other tissues analysed by DNA methylation arrays (Extended Data Fig. 4m). While DNA methylation arrays contain a subset of CpG sites with biased representation of certain genomic features (Extended Data Fig. 1e; Extended Data Fig. 4n), correcting for CpG numbers per feature representation, confirmed hypermethylated DMRs to be predominantly located at Polycomb-regulated promoters and regulatory regions across tissues (Extended Data Fig. 1e,f; Extended Data Fig. 4m-o)."

Additionally, the authors should be explicit in each figure legend about the technology used to measure DNA methylation, as it may be useful to the reader in the interpretation of the results (for example, comparing Fig. 1e and Fig. 4b, notice the detection of 4 times more hypermethylated DMRs with EM-seq compared to DNA methylation arrays).

We have amended figure legends to ensure clarity on the technology used.

#3. One of the hallmarks of aging is the decrease of DNA methylation, as indicated in the study from which the authors reanalyzed the DNA methylation patterns in newborn and centenarian individuals (PMID: 22689993). While the authors mostly focus on the DNA hypermethylation happening in Polycomb-marked regions, it is unclear to this reviewer whether there are any DNA hypomethylated regions in the DNMT3A GOF mouse model at all, which may not be detected using a DMR approach (as usually DMR analysis clusters adjacent CpGs with similar methylation changes, and the CpGs that lose methylation during aging tend to be found in CpG-poor regions of the genome). The authors should complement their current analysis of EM-seq data (Fig. 4) with additional genome-wide analysis focusing on DNA methylation changes occurring at different genomic scales than DMRs, including at the CpG-level as well as the megabase-scale. For analysis at the CpG-level, see an example by the Schübeler lab (PMID: 22170606, Fig. 5b).

We thank the reviewer for this suggestion. We have undertaken additional analyses of our EM-seq data to address whether the hypomethylation characteristic of aging also occurs in our mice. However, we do not detect meaningful hypomethylation at either the individual CpG scale or at larger genomic scales (10kbp-1Mbp). We therefore conclude that there is no evidence of age-related hypomethylated regions in HESJAS. These analyses are incorporated in Extended Data Fig. 6f, and provide additional support to our conclusion that HESJAS mutations specifically cause hypermethylation of Polycomb-marked regions, and that associated phenotypes are consequent on these DNA methylation gains.

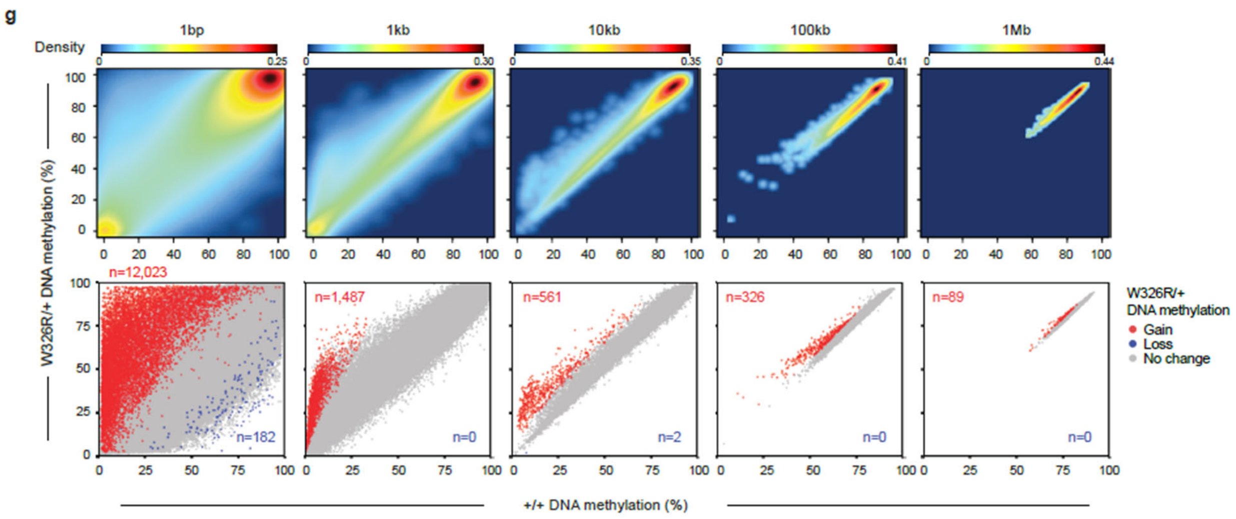

We also state in the results text:

"As DMR analysis is based on close proximity of methylated CpGs, widely dispersed hypomethylation in CpG poor regions characteristic of aging[23] might be overlooked. Therefore, we also analysed our EM-seq dataset (that identifies CpG methylation sites genome wide at single nucleotide resolution) independent of DMR calling, at single-CpG-site, 10-kbp, 100-kbp, and 1-Mbp resolution. This identified a very limited number of hypomethylated sites, but importantly we did not discover larger-scale regions with reduced methylation that are characteristic of partially methylated domains (PMDs) seen in aging. We therefore concluded that the Dnmt3a^{W326R} mutation causes Polycomb-region-specific hypermethylation in HSCs."

As well, to further address this point in an independent manner, we have undertaken an analysis of the patient samples using an epigenetic clock designed to detect cell division-associated hypomethylation in CpG-poor regions which occurs with aging[24]. This 'HypoClock' did not show an increased clock score in HESJAS patients (Reviewer Figure x), while a similar clock detecting DNA hypermethlation (EpiToc2) from the same paper detected significantly increased clock score.

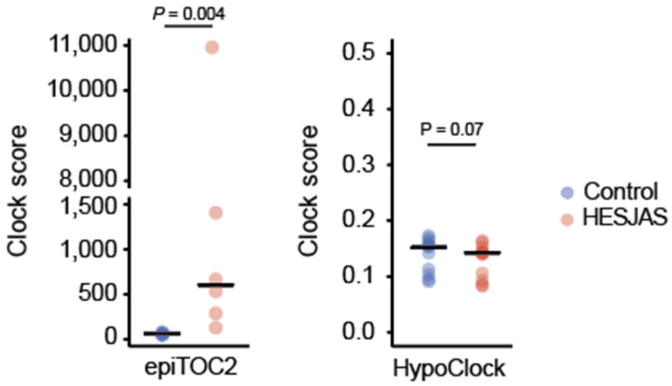

Reviewer Fig. 4| Epigenetic hypo- and hyper-methylation clock comparison. Data points, individual samples; black line, median; *P* values, Wilcoxon test.

#4. In Fig. 5, when the authors focus on DNA methylation changes during human aging using the datasets from PMID: 22689993 (datasets in GSE31263), although the authors refer to those samples as "lymphocytes", the authors should take into account that of those datasets, the 0yr and 103yr samples come from CD4+ T cells, while the 26yr sample may come from PBMCs, which include cells other than lymphocytes. Please check and make the appropriate modifications in the text. Additionally, please make sure to use an appropriate H3K27me3 dataset given this clarification. Currently it is unclear what is the source of the H3K27me3 dataset employed in Fig. 5 (see next comment).

We apologise, we had overlooked the inconsistency in sample type between subjects in the published study from which we obtained this data. We thank the reviewer for bringing this to our attention, and have amended the text and figure legend accordingly, as advised.

The H3K27me3 track provided was from a lymphoblastoid cell line dataset. To provide the best match with the naïve CD4+ T cells in the new born and 103-year old, we have obtained data from the ENCODE project for this cell type (GSM772868) and updated the track accordingly.

#5. Throughout the manuscript, the authors should indicate explicitly in the figure legend the accession number of the dataset they are using, to avoid any ambiguities. For example, in Fig. 5, it is unclear what is the source of the H3K27me3 dataset being used, and in the Data and materials availability section of the manuscript, the authors are listing as potential source for this dataset an accession number which includes 296 datasets from the ENCODE project across several different cell lines. Furthermore, the authors should aim to cross-compare their datasets to closely related cell types when possible. In EDF. 5c, the authors are presumably using a H3K4me3 dataset from the GM12878 lymphoblastoid cell line, whereas there are datasets in primary T cells publicly available (for example, GSM1586403). Similarly, work by the labs of Ido Amit (PMID: 25103404), Maria Figueroa (PMID: 31085557), Margaret Goodell (PMID: 32386543), and others, have produced several datasets in primary cell types relevant to this manuscript that could be used for reanalysis instead of the ENCODE datasets originating from cell lines that are presumably currently being used.

We apologise that in Fig. 5 the data source was not specifically identified by the accession number given from the ENCODE project. The dataset was from a lymphoblastoid cell line. We thank the reviewer for their suggestion of alternative datasets. To address the reviewer's comment, we now include accession numbers for datasets in figure legends for Fig. 4d, Fig. 6a and Extended Data Fig. 6e,f regenerating these figures using ChIP-seq datasets that match murine HSCs and naïve CD4+ve human lymphocytes, respectively.

#6. In lines 151-157, the authors mention that many of the HESJAS patients suffer from lymphopenia, in some cases even leading to severe immunodeficiency, and while it is also said that the mutant mice suffer from low bone marrow cellularity, in the lines 220-221 it is pointed out that these mice do not have reductions of either HSCs or MPPs. Is this also the case in humans for the HESJAS patients? Please comment in the manuscript text.

Bone marrow examinations have only been performed on P1 and P11, both now deceased. Stem cell numbers are not routinely assessed clinically, and as these biopsies are historical, we have not yet been able to address whether stem cell numbers are altered in humans. Bone marrow biopsy as an invasive procedure is not covered by our ethical approval, so we will have to wait for an opportunity to address this question in future, when a bone marrow biopsy is taken from one or more of our cases for clinical management reasons. We detail the results from the bone marrow investigations for P1 and P11 in Supplementary Data Table 2, and comment as requested in the results section:

"Haematopoesis is a deeply characterised multilineage stem cell system and given haematological abnormalities in our cohort including lymphopenia and bone marrow hypocellularity, we focussed our investigation on HSCs and their progeny. However, more detailed interogation such as the status of HSCs was not feasible in human subjects, so we turned to the Dnmt3a$^{W326R/+}$ mouse model".

Minor comments

#1. In Fig. 2f, please add to the figure the cell surface markers used to define "myeloid" and "lymphoid" in the flow cytometry analysis.

CD11b, Gr1; and CD19 defined myeloid and lymphoid populations respectively. We have added this information to the figure panel.

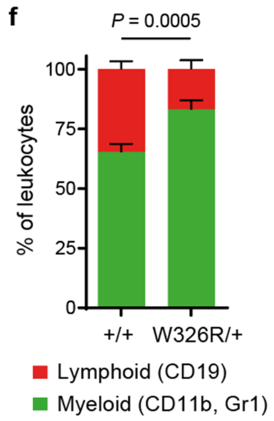

#2. In EDF. 1c, please consider replacing the black-scale for some type of color-scale (for example, rainbow), and state which technology was used for this DNA methylation measurement.

As requested, we have added colour scale and technology used.

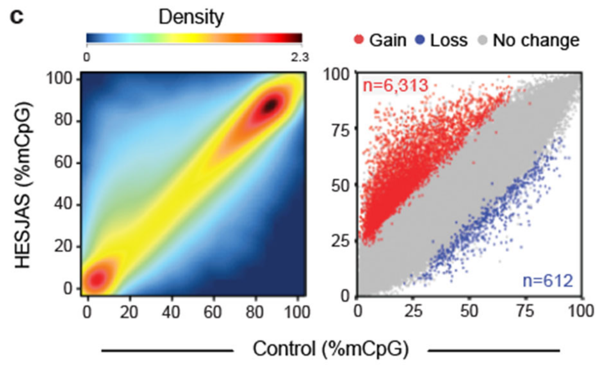

#3. In EDF. 1f, please add an explanation about how DMRs were assigned to genes.

This has been added to the figure (now Extended Data Fig. 1g), and is also documented in the methods section for Gene Otology enrichment analysis:

Extended Data Fig. 1g "Gene Ontology analysis of hypermethylated DMR associated genes. Top hits ranked by Benjamini-Hochberg adjusted FDR significance level. DMRs mapped to their closest annotated TSS. Genes associated with DMR probes (n = 623 genes) versus genes associated with all array probes (n = 20,028), two-sided Fisher's exact tests."

#4. In EDF. 4l, please consider adding an additional barplot normalized to 100%, illustrating whether the CFU proportions are the same in DNMT3A GOF cells vs WT, even if the total CFU numbers are decreased in DNMT3A GOF cells.

As requested, we have now added this what is now Extended Data Fig. 5l

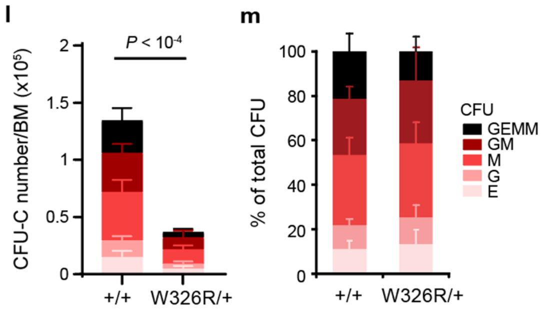

#5. In lines 276-278, the manuscript mentions that the DNMT3A GOF mice display an enrichment of DNA hypermethylation at “bivalent transcription start sites/promoters marked by H3K4me3 and H3K27me3 (Extended Data Fig. 5a, …)”, but the figures referenced should be EDF. 5b-c.

Thank you, we have corrected this

Reviewer #3 (Remarks to the Author):

In this manuscript, the authors explore age-associated DNA hypermethylation and its potential role in causing ageing-related phenotypes. Drawing on both a cohort of individuals with Heyn-Sproul-Jackson syndrome (HESJAS), caused by gain-of-functon(GOF) mutations in DNMT3A, and a Dnmt3aW326R/+ mouse model, they observe age-associated phenotypes in both human and mice. Aiming to investigate the cellular basis of these phenotypes, they show stem cell dysfunction in adult Dnmt3aW326R/+ mice. Furthermore, accumulation of DNA hypermethylation at Polycomb-marked regions are observed. This manuscript addresses an important and unresolved question in the field of epigenetic ageing. The authors establish an interesting monogenic ageing model, and integrate functional stem cell assays, methylome and transcriptomic profiling, and clinical phenotyping to argue that DNA hypermethylation underlies stem cell exhaustion and downstream tissue pathology. However, while the study is ambitious, there are several concerns that dampened my initial enthusiasm after reading the abstract.

Comments:

1. My major concern is that this study seems to be a follow up on the previous work published on HESJAS by the same group. While this is appreciated and of course relevant for understanding HESJAS, the links to age-related changes in DNA methylation are correlative, and the direct interpretation of how this disease relates to tissue ageing is unclear.

The reviewer highlights two issues: (a) HESJAS has been previously described, and (b) the correlative relationship of DNA hypermethylation to physiological ageing.

a) Our previous study reported HESJAS as a disorder of restricted growth, with DNMT3A mutations causing microcephalic dwarfism (Heyn et al Nat Genet 2019). Ageing phenotypes were not recognised by ourselves at this time. Since then, through the identification of a larger number of HESJAS patients, along with availability of more clinical data, as well through thorough phenotypic analysis of our HESJAS mouse model we came to realise that premature ageing was present in both, and performed further experiments to determine the underlying mechanism. In the current paper, we present these new findings and establish that HESJAS is actually an accelerated ageing syndrome (progeria) both in humans and mice. To our knowledge, it is the first monogenic progeria syndrome to be caused by a core epigenetic enzyme. Progeroid disorders have provided important causal evidence for hallmark ageing mechanisms, and so we believe in itself this is a significant advance for the ageing field, as these conditions are valuable for experimental studies determining the contribution of a single hallmark ageing process in isolation. While growth and ageing are temporally distinct, it is important to note that other monogenic progeroid syndromes (e.g. Hutchinson Guilford Progeria, Werner Syndrome), and progeria mouse models[1,2,25,26] also have restricted growth (also see Reviewer Figure 1 above, in response to Reviewer 2). This aspect of HESJAS is therefore also consistent with a monogenic progeria.

In response to the reviewer's comment, we have revised our introduction to further clarify the prior identification of DNMT3A gain of function mutations causing growth failure as HESJAS syndrome, which led us to the current work in which we discover that HESJAS is in fact a novel type of accelerated ageing syndrome.

"DNA hypermethylation also occurs in Heyn-Sproul-Jackson syndrome (HESJAS) [27,28]*, in which gain-of-function mutations in DNMT3A (hereafter denoted DNMT3A^{GOF}) cause microcephalic dwarfism (a group of extreme growth restriction disorders). … Here, we report the discovery that individuals with HESJAS display multiple age-associated phenotypes, in addition to the previously reported growth phenotype."*

In our results we also contextualise this seeming disconnect between HESJAS as a growth disorder, and an ageing syndrome, by highlighting that reduced size is a common feature of monogenic progerias in both humans and mice.

"Here, we report HESJAS as a novel form of monogenic progeria driven by gain of function in DNMT3A, an enzyme central to mammalian epigenetic regulation. HESJAS was previously identified as a disorder of extreme size reduction (microcephalic dwarfism). On the face of it this presents a disconnect, given growth and ageing are temporally distinct processes. However, reduced size is commonly seen in other monogenic accelerated ageing syndromes and progeroid mouse models[1,2,25,26] *."*

(b) Correlation to age-related hypermethylation

DNA methylation in ageing occurs at Polycomb regions and we show that the same sites are methylated in HESJAS, now with strengthened evidence, after responding to comments from Reviewer 2. Therefore, HESJAS provides a model to understand the functional and pathological consequences of such hypermethylation at these locations. As monogenic disorders provide unambiguous causal links between genotype and phenotype, i.e. in this case between mutation and cell, tissue and organism phenotypes; hence, DNMT3A mutations in HESJAS are causally linked to the age-associated pathologies we report, just like they are causally linked to the observed hypermethylation (diagram below).

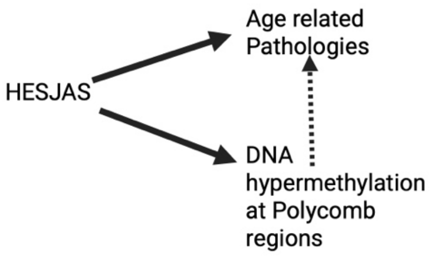

The key outstanding issue is then whether the hypermethylation is correlated with the pathology or causal for it. Applying Occam's razor, causality is highly likely given DNMT3A's canonical role as a DNA methyltransferase, alongside the previously established correlation between hypermethylation and ageing. However, we acknowledge that this does not provide direct mechanistic evidence. So, by addressing this reviewer's point 4 below, we now provide experimental evidence in favour of causality. We show that hypermethylation at the *Pax5* promoter severely impacts on its transcriptional activation during lymphocyte development. As this transcription factor is essential for B cell development this provides direct linkage for hypermethylation to an age-related pathology, the observed B cell lymphopenia. Furthermore the critical dependence on DNA hypermethylation at this locus is demonstrated, as DNA methylation at this specific locus is selected against in cells progressing past this critical maturation step.

This data is now provided as Fig. 5 of the revised manuscript (and below for the reviewer, in response to point 4), and we discuss the above points in the main text:

"*Given DNMT3A's canonical function as a DNA methyltransferase, and the direct effects we demonstrate between Pax5 hypermethylation, Pax5 gene activation and lymphopenia, this implicates hypermethylation as the cause, at least of this pathology*."

2. Along the same lines, there is little mechanistic insight on how the HESJAS-specific PWWP mutation can be used as a model to study age related changes in methylation that occur in presence of wt DNMTs. Why does impaired binding to H3K36me mimic methylation changes observed in aged tissues?

Wild-type DNMT3A1 (the longest isoform of DNMT3A) is competitively attracted by two distinct chromatin marks[12,13]: H3K36me2/3 and H2AK119Ub, which are present in mutually exclusive genomic regions (model below, Extended Data Fig. 10g). The PWWP-H3K36me2/3 interaction normally restrains DNMT3A1 methylation of Polycomb regions containing H2AK119Ub marks. As overexpression of DNMT3A1 can be sufficient to methylate Polycomb

regions[28] and wild-type DNMT3A1 is distinctive from the other *de novo* DNA methyltransferase, DNMT3B, in having an N-terminal UDR domain mediating this K119Ub interaction, our model is that wild-type DNMT3A gradually methylates CpGs at Polycomb sites during physiological ageing. In HESJAS, PWWP mutations disrupt the interaction with H3K36me2/3, enhancing the attraction of DNMT3A1 to Polycomb marked regions, accelerating the rate of DNA methylation gain in these regions, leading to onset of associated pathologies. Hence, HESJAS provides an accelerated model for the action of wild-type DNMT3A at Polycomb regions, by tipping the balance in favour of the UDR-K119Ub interaction.

We have revised the discussion accordingly and provide further explanation with a graphical model in Extended Data Fig. 10:
"In physiological ageing, gradual DNA hypermethylation by wild-type DNMT3A in the population at large may be accounted for by the affinity of the DNMT3A1 N-terminal region for H2AK119Ub[12,13], an epigenetic mark enriched in Polycomb regions (see model in Extended Data Fig. 10g)."

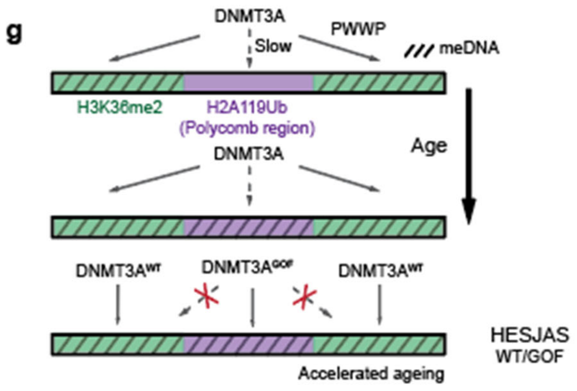

Extended Data Fig. 10| g, Model: hypermethylation of Polycomb regions by wild-type DNMT3A during physiological ageing. Wild-type DNMT3A1 is competitively attracted by two distinct chromatin marks, H3K36me2/3 and H2AK119Ub which are present in mutually exclusive genomic regions[10,21]. The PWWP-H3K36me2/3 interaction normally restrains DNMT3A1 methylation of Polycomb regions containing H2AK119Ub marks. Overexpression of DNMT3A1 can be sufficient to methylate Polycomb regions[96] and wild-type DNMT3A1 is distinctive from the other *de novo* DNA methyltransferase, DNMT3B, in having an N-terminal UDR domain mediating this K119Ub interaction. Therefore, low-level wild-type DNMT3A could gradually methylate CpGs at Polycomb sites during physiological ageing. In HESJAS, PWWP mutations disrupt the H3K36me2/3 enhancing the attraction of DNMT3A to Polycomb marked regions, accelerating the rate of DNA methylation, and the onset of associated pathologies. Hence, HESJAS provides an accelerated model for wild-type Dnmt3a's action at Polycomb regions, by tipping the balance in favour of the UDR-K119Ub interaction.

3. The authors' model suggests that methylation is the key variable, but alternative epigenetic or genetic effects of the mutation are not discussed. The observed phenotypes may be the downstream result of broader molecular disruptions caused by the PWWP domain mutations. E.g., could the mutation in the PWWP domain of DNMT3A disrupt protein complexes at target genes required for differentiation independently of its methylation activity? The authors should temper causal claims and consider that hypermethylation might be a readout of dysregulation rather than the initiating cause.

We agree that alternative mechanisms are important to consider. Indeed, work published since submission raises the possibility of non-catalytic functions for DNMT3A[29], however the molecular mechanism by which this might occur remain to be determined.

We note that putative non-catalytic DNMT3A functions might well be accounted for by recruitment of catalytically active DNMT3B, in a heteromeric complex, a notion supported by the demonstration that we and others have shown that catalytically inactive DNMT3B can recruit catalytically active 3A in recent studies[30,31]. Futhermore, the cellular abundance (~4,000 molecules per cell in haematological tissues[32]) and broad genomic distribution of both wildtype and DNMT3A^{W330R} enzymes detected by ChIPseq[12,33] reduces the likelihood of a direct specific disruptive effect of DNMT3A binding at target genes (Reviewer Fig. 5).

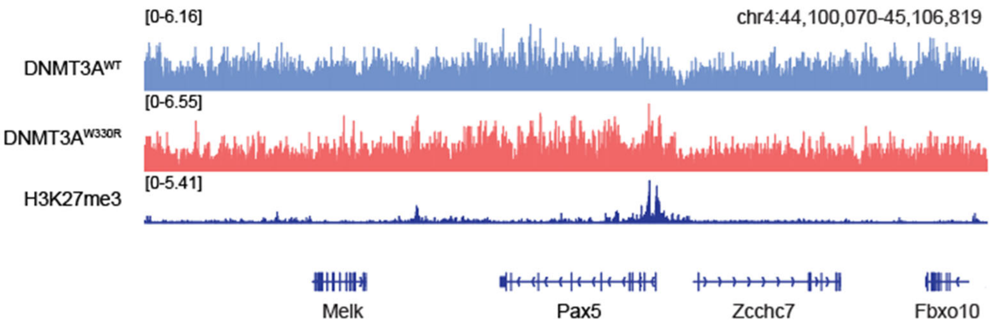

Reviewer Fig. 5| Genomic distribution of WT and W330R DNMT3A. Browser view of Pax5 locus showing DNMT3A ChIP-seq tracks, DNMT3A^{WT} (GSM7882128) and DNMT3A^{W330R} (GSM7882131), and H3K27me3 (GSM1000089).

Most significantly, we now directly demonstrate that DNA methylation at the *Pax5* promoter is increased, while *Pax5* transcriptional activation is reduced during B cell differentiation in *Dnmt3a*$^{W326R/+}$ mice. This, along with selection of more differentiated B cells without methylation at the *Pax5* locus argues strongly in favour of a direct role of hypermethylation at Polycomb regions, rather than a broader molecular disruption.

To address the reviewer's comment, tempering causal claims, we now discuss the possibility of a non-catalytic function alongside evidence for a direct role of hypermethylation in pathology:

"Given DNMT3A's canonical function as a DNA methyltransferase, and the direct effects we demonstrate between Pax5 hypermethylation, Pax5 gene activation and lymphopenia, this implicates such hypermethylation as the cause, at least of this pathology. Dnmt3a was recently reported to have non-catalytic functions[29], meaning that HESJAS mutations might mediate their effects independently of Dnmt3a's methyltransferase activity. *However, the strong selection against Pax5 hypermethylation in late stage differentiation and mature B cells (Fig. 5g,h) argues in favour of the direct effect of DNA hypermethylation."*

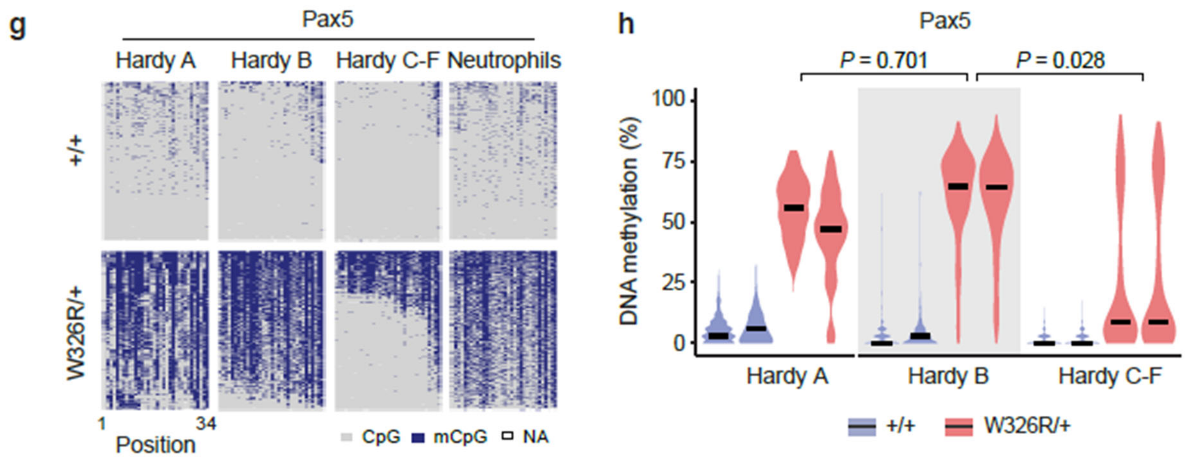

4. The scRNA-seq data show modest transcriptional changes between wild-type and Dnmt3aW326R/+ progenitor cells, despite pervasive hypermethylation at Polycomb-marked regions. This raises the question about the functional impact of the methylation changes. The authors propose that transcriptional differences emerge during differentiation, due to impaired induction of lineage genes. This is plausible, but the modest expression changes weaken the case for direct repression of gene expression via DNA methylation.

We agree that transcriptional effects detected in HSPCs were modest and this has led us to examine lymphopoeisis, where we demonstrate strong transcriptional effects of hypermethylation on Pax5 transcription, consistent with our proposed model.

An alternative explanation could be that delayed differentiation is due to cell cycle perturbation or altered proliferation dynamics rather than slowed transcriptional activation.

Transcriptional evidence for altered cell cycle is not evident across pseudotime, nor is Ki-67 transcription (a marker used to distinguish proliferating cells from quiescent cells). Cell cycle determined by DNA content FACS for HSPC are also similar between mutant and wild-type HSPC populations. So, proliferation dynamics and cell cycle do not appear to be significantly altered in HSPCs. Furthermore, to exclude an intrinsic cell proliferation defect across cell-types, we also performed growth curves on cultured primary dermal fibroblasts from our mice. We found Dnmt3a$^{W326R/+}$ fibroblasts to proliferate as well as wild-type.

We address the reviewer's point, by providing the following textual addition:

"No changes in cell cycle or cell proliferation, as measured by S-phase, G2/M scores and by Ki67 expression, were detected in the scRNAseq analysis nor were cell cycle changes evident with FACS analysis of HSPC subpopulations (Extended Data Fig. 8). Likewise Dnmt3a$^{W326R/+}$ primary dermal fibroblasts grew as well as wildtype fibroblasts in culture, excluding an intrinsic cell proliferation defect across cell types (Extended Data Fig. 8d)."

Extended Data Figure 8

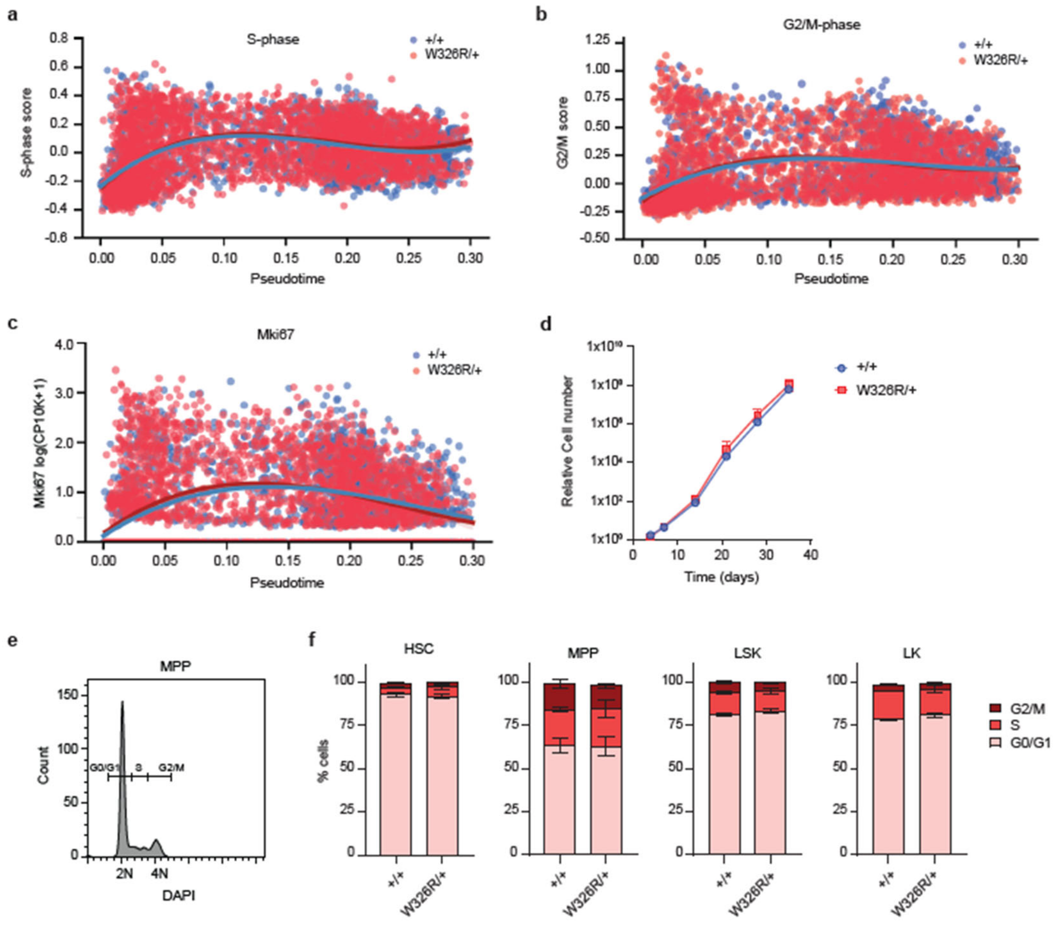

Extended Data Fig. 8| Dnmt3a$^{W326R/+}$ cell cycle dynamics are similar to wildtype HSPCs. a,b, scRNA-seq analysis of cell cycle signature genes across the myeloid differentiation trajectory. Scatterplots of transcriptional cell cycle scores for S-phase signature genes across pseudotime (**a**) and G2/M signature genes (**b**). Permutation test for W326R/+ vs +/+: p=0.54 (>0.05, ns) for **a**; p=0.76 (>0.05, ns) for **b. c,** Scatterplot of *Mki67* gene expression along myeloid differentiation trajectory. Permutation test for W326R/+ vs +/+: p=0.33 (>0.05, ns). **d,** Growth curves: cell proliferation is not decreased in Dnmt3a$^{W326R/+}$ cultured primary dermal fibroblasts compared with isogenic wild-type controls. **e,f,** HSPC FACS analysis of cell cycle. **e,** Representative DNA content histogram of cell populations defined by LSK-SLAM markers. **f,** Quantification of cell cycle profiles in HSC, MPP, LSK and LK populations, as indicated. No significant differences were detected between genotypes detected by 2-way ANOVA for HSCs, MPPs, LSK or LK populations.

A clearer explanation of why transcriptional changes are so limited and how this supports the proposed mechanism are necessary.

The major reduction in cell numbers occured later in haematopoesis during lineage commitment and differentiation, with most profound reductions during lymphopoiesis (Extended Data Fig. 5a-d). Therefore, to address the criticism that “the modest expression changes weaken the case for direct repression of gene expression via DNA methylation”, we focused revision studies on characterising lymphocyte differentiation. Significantly, DMVs encompassing the transcriptional start site and regulatory regions of *Pax5* are hypermethylated in HSCs from W326R/+ mice. This is accompanied by marked reduction in *Pax5* transcript levels during early stages of B cell differentiation when *Pax5* expression is normal induced. Furthermore, this is associated with substantial reduction in cell number from Hardy stage B onwards, the time at which Pax5 is essential for differentiation stage

progression, acting as a master regulator of B-cell development[34,35]. As well, for W326R/+ B cells that do undergo VDJ rearrangement, distal V gene usage is also reduced, consistent also with the established role of Pax5 in promoting distal V gene recombination[36,37]. Finally, B cells surviving the transition from Hardy stage B to later differentiation stages, largely lose hypermethylation at the Pax5 promoter, suggesting they are under strong selection against hypermethylation at the Pax5 locus.

Taken together, we believe these findings provide a clear explanation and support for our proposed mechanism, substantially strengthening the case for DNA hypermethylation directly impacting gene transcription, to reduce cell number during haematopoesis.

We respond to the reviewer's point, by incorporation of a new Figure 5, along with the following results text:

*"**Hypermethylation of Pax5 impairs activation in early B cell development***

To gain further insight into how bone marrow hypocellularity arose we turned to later differentiation, where the greatest changes in cell number were observed (Extended Data Fig. 5a-e). HESJAS DMRs intersect with lineage-defining genes, with the highest ranking categories from Gene Ontology analysis containing many transcription factor genes (Extended Data Fig. 6d, Supplementary Table 6). Most of these transcription factors will not be activated along a specific differentiation trajectory. Indeed, the expectation would be that only one, or at most a few, would be expressed in a given lineage. We identified several candidate genes from the haematopoesis literature[38-54], mapping to different myeloid and lymphoid lineages (Extended Data Fig. 9a). As B lymphocyte numbers are the most severely impacted in HESJAS, we then focused our attention on early lymphogenesis. The transcriptional programme for B lymphocyte differentiation involves several transcription factors, including PAX5, a master regulator essential for the transition from pre-pro B cells to pro B cells[34] (Hardy A to B, Fig. 5a). The Pax5 promoter and regulatory regions are hypermethylated in HSCs from Dnmt3a$^{W326R/+}$ mice (Fig. 5b). According to dogma, this would be expected to transcriptionally silence the Pax5 gene. To confirm this prediction, early B cell populations were FACS-sorted using established cell-surface markers into Hardy stage fractions (Fig 5a, Supplementary Fig. 3), which represent sequential steps in early B cell development during which Pax5 transcription is activated. RT-qPCR demonstrated that while wild-type pro B-cells (Hardy B) robustly activated Pax5 transcription, this was severely compromised in Dnmt3a$^{W326R/+}$ pro-B cells (Fig. 5c). In contrast to normal pre-pro B (Hardy A) cell numbers for W326R/+ mice, there was a marked reduction in pro B and pre B cells (Hardy B-D), suggesting that differentiation was largely blocked at this transition (Fig. 5d), consistent with insufficient Pax5 expression in mutant cells. Furthermore, in line with a key role for Pax5 in promoting distal V-gene segment recombination[36,37], productive VDJ recombination was reduced, with remaining VDJ events strongly biased towards proximal V gene incorporation (Fig. 5e-f). Finally, while DNA hypermethylation was evident in Hardy A and B fractions, this methylation was notably absent from the majority of Dnmt3a$^{W326R/+}$ pre B, immature and mature B cells (Hardy fractions C -F; Fig. 5g,h), whereas hypermethylation remained at Pax5 in neutrophils and at other loci in B cells (Extended Data Fig. 9c-e). This indicates selective pressure against Pax5 DNA methylation for the cells that did progress to later differentiation stages consistent with cells with the least abrogated expression of Pax5

being able to survive the pro B to pre B transition." Taken together, we conclude that hypermethylation of the Pax5 gene significantly impairs its transcriptional activation at a critical development stage in early B cell development, leading to reduced lymphocyte number, providing a mechanistic explanation for the lymphopenia associated with HESJAS. "

Figure 5

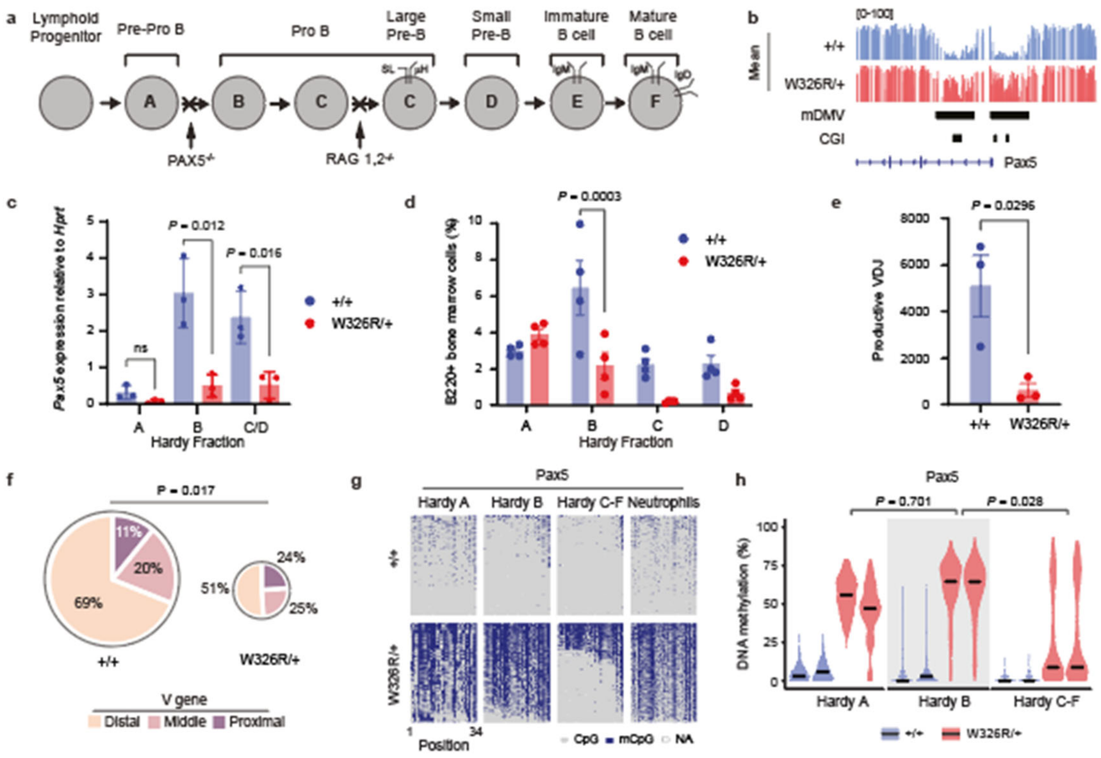

Figure 5. HESJAS B cell differentiation is delayed at the same stage as $Pax5^{-/-}$ bone marrow. a, Schematic of B cell differentiation stages (adapted from [93,94]). **b,** DMV hypermethylation at the 5' end and promoter region of *Pax5* in $Dnmt3a^{W326R/+}$ HSCs. Genome browser view, mean DNA methylation from *n* = 9 mice per genotype. chr4:44,685,675-44,729,761; EM-Seq. **c,** RT-qPCR analysis demonstrates reduced Pax5 mRNA expression levels in B-cell Hardy fractions B and C/D in 3-month old $Dnmt3a^{W326R/+}$ mice. Mean ± SD, unpaired t-tests. **d,** B-cell numbers are reduced from Hardy B stage onwards. Data points, 2-3 month old mice, mean ± SD, t-tests. **e,** Reduced productive VDJ recombination events. Data points, 2-3 month old mice, mean ± SD, t-tests. **f,** VDJ recombination is bias towards proximal V gene usage, chi-squared test. Percentage VDJ recombination events with proximal, middle and distal V genes. **g,h,** *Pax5* promoter hypermethylation is reduced at later stages of B cell differentiation. Targeted EM-PCR analysis of DNA methylation of a 562bp amplicon at the *Pax5* promoter region. **g,** Representative CpG methylation state matrices of sorted cell populations from individual mice, ages 2-3 months. Blue, mCpG; grey, CpG. Columns, individual CpGs numbered from start of amplification. Rows, individual sequencing reads. (Panels reproduced in EDF9 alongside replicate data at higher magnification.) **h,** Violin plot. Percentage DNA methylation of individual amplicons. Centre line, median. *P* value, two-sided t-test between population means for *n*=2 mice.

5. The claim that lineage-defining genes show reduced expression across pseudotime in mutant cells is interesting but required additional clarification. It is unclear whether this observation reflects a reduction in cell numbers progressing to late stages or a true defect in transcriptional activation of target genes.

We believe this is a true reduction in transcriptional activation, because our scRNAseq demonstrated that Fcgr3, Csf2ra, Ncam1 and Gpc1 transcription were reduced for individual cells that were at the same point in pseudotime (i.e. these cells had a transcriptional

phenotype that matched the same stage of differentiation as wild-type cells; see Fig. 4k and Extended Data Fig. 7f). We provide a model in Extended Data Fig. 7e for further explanation of how this distinguished between (the appearance of) decreased transcription due to less cellularity at later timepoints, and a decrease in individual cells that have reached these same advanced timepoints. Furthermore, our characterisation of Pax5 transcription during B cell development, provides additional evidence on this point, as qRT-PCR analysis of FACS-sorted B cells at well characterised differentiation stages defined by established cell surface markers, confirms that there is a defect in transcriptional activation .

6. Although the manuscript states that hypermethylation affects Polycomb domains and bivalent promoters, the gene-level analysis of methylation changes remains limited. A more systematic intersection between DMRs and curated lists of lineage-defining genes is missing.

Gene Ontology analysis of HSC hyperDMRs demonstrates enrichment for many transcription factors (Extended Data Fig. 6d), genes that have clear roles in lineage-definition. We now provide a list of these genes (Supplementary Table 6). Most of the genes listed will be required in other tissue contexts and not be activated during HSC development. Therefore from the haematopoesis literature[38-54] we have identified key transcription factors that are required for lineage differentiation, confirming that there are lineage-specific transcription factors that are targets of hypermethylation (Extended Data Fig. 9a). One of these genes is *Pax5*, a key determinant of B-cell development, that we have gone on to characterise functionally, as as outlined above in response 3.5 above.

We als include textual revisions in the results section to respond to the reviewer:

"HESJAS DMRs intersect with lineage-defining genes, with the highest ranking categories from Gene Ontology analysis containing many transcription factor genes (Extended Data Fig. 6d, Supplementary Table 6). Most of these transcription factors will not be activated along a specific differentiation trajectory. Indeed, the expectation would be that only one or at most a few would be expressed in a given lineage. We identified several candidate genes from the haematopoesis literature[38-54], mapping to different myeloid and lymphoid lineages (Extended Data Fig. 9a)."

1. Weeda, G. *et al.* Disruption of mouse ERCC1 results in a novel repair syndrome with growth failure, nuclear abnormalities and senescence. *Curr Biol* **7**, 427-39 (1997).
2. Mounkes, L.C., Kozlov, S., Hernandez, L., Sullivan, T. & Stewart, C.L. A progeroid syndrome in mice is caused by defects in A-type lamins. *Nature* **423**, 298-301 (2003).
3. Kunstyr, I. & Leuenberger, H.G. Gerontological data of C57BL/6J mice. I. Sex differences in survival curves. *J Gerontol* **30**, 157-62 (1975).
4. Lu, A.T. *et al.* DNA methylation GrimAge strongly predicts lifespan and healthspan. *Aging (Albany NY)* **11**, 303-327 (2019).
5. Levine, M.E. *et al.* An epigenetic biomarker of aging for lifespan and healthspan. *Aging (Albany NY)* **10**, 573-591 (2018).
6. Horvath, S. DNA methylation age of human tissues and cell types. *Genome Biol* **14**, R115 (2013).

7. Hannum, G. *et al.* Genome-wide methylation profiles reveal quantitative views of human aging rates. *Mol Cell* **49**, 359-367 (2013).
8. Meer, M.V., Podolskiy, D.I., Tyshkovskiy, A. & Gladyshev, V.N. A whole lifespan mouse multi-tissue DNA methylation clock. *Elife* **7**(2018).
9. Challen, G.A. *et al.* Dnmt3a is essential for hematopoietic stem cell differentiation. *Nat Genet* **44**, 23-31 (2011).
10. Jeong, M. *et al.* Loss of Dnmt3a Immortalizes Hematopoietic Stem Cells In Vivo. *Cell Rep* **23**, 1-10 (2018).
11. Crofts, S.J.C., Latorre-Crespo, E. & Chandra, T. DNA methylation rates scale with maximum lifespan across mammals. *Nat Aging* **4**, 27-32 (2024).
12. Weinberg, D.N. *et al.* Two competing mechanisms of DNMT3A recruitment regulate the dynamics of de novo DNA methylation at PRC1-targeted CpG islands. *Nat Genet* **53**, 794-800 (2021).
13. Gu, T. *et al.* The disordered N-terminal domain of DNMT3A recognizes H2AK119ub and is required for postnatal development. *Nat Genet* **54**, 625-636 (2022).
14. Alveal-Mellado, D. & Giménez-Llort, L. Thigmotaxis Helps Differentiate Normal and Pathological Ageing Processes in a Mouse model of Alzheimer's Disease. in *2nd International Electronic Conference on Brain Sciences* Vol. 8 (ed. Meriney, S.D.) 2 (Med. Sci. Forum 2022).
15. Mayagoitia, K. *et al.* Loss of APP in mice increases thigmotaxis and is associated with elevated brain expression of IL-13 and IP-10/CXCL10. *Physiol Behav* **240**, 113533 (2021).
16. Liu, X. *et al.* Resurrection of endogenous retroviruses during aging reinforces senescence. *Cell* **186**, 287-304 e26 (2023).
17. Lee, S. *et al.* Comparison between surrogate indexes of insulin sensitivity and resistance and hyperinsulinemic euglycemic clamp estimates in mice. *Am J Physiol Endocrinol Metab* **294**, E261-70 (2008).
18. Gray, S.L. *et al.* Leptin deficiency unmasks the deleterious effects of impaired peroxisome proliferator-activated receptor gamma function (P465L PPARgamma) in mice. *Diabetes* **55**, 2669-77 (2006).
19. Groeneveld, M.P., Huang-Doran, I. & Semple, R.K. Adiponectin and leptin in human severe insulin resistance - diagnostic utility and biological insights. *Biochimie* **94**, 2172-9 (2012).
20. Kwok, A. *et al.* Truncation of Pik3r1 causes severe insulin resistance uncoupled from obesity and dyslipidaemia by increased energy expenditure. *Mol Metab* **40**, 101020 (2020).
21. Semple, R.K. *et al.* Postreceptor insulin resistance contributes to human dyslipidemia and hepatic steatosis. *J Clin Invest* **119**, 315-22 (2009).
22. Virtue, S. & Vidal-Puig, A. GTTs and ITTs in mice: simple tests, complex answers. *Nat Metab* **3**, 883-886 (2021).
23. Zhou, W. *et al.* DNA methylation loss in late-replicating domains is linked to mitotic cell division. *Nat Genet* **50**, 591-602 (2018).
24. Teschendorff, A.E. A comparison of epigenetic mitotic-like clocks for cancer risk prediction. *Genome Med* **12**, 56 (2020).
25. Oshima, J., Martin, G.M. & Hisama, F.M. Werner Syndrome. in *GeneReviews((R))* (eds. Adam, M.P. *et al.*) (Seattle (WA), 1993).

26. Gordon, L.B., Brown, W.T. & Collins, F.S. Hutchinson-Gilford Progeria Syndrome. in *GeneReviews((R))* (eds. Adam, M.P. *et al.*) (Seattle (WA), 1993).
27. Heyn, P. *et al.* Gain-of-function DNMT3A mutations cause microcephalic dwarfism and hypermethylation of Polycomb-regulated regions. *Nature Genetics* **51**, 96-105 (2019).
28. Heyn-Sproul-Jackson Syndrome. 03/25/2024 edn MIM Number: 618724 (Johns Hopkins University, Baltimore, MD, Online Mendelian Inheritance in Man, OMIM®.).
29. Xavier Raj, I. *et al.* Non-canonical functions of DNMT3A in hematopoietic stem cells regulate telomerase activity and genome integrity. *Cell Stem Cell* **32**, 1251-1266 e8 (2025).
30. Duymich, C.E., Charlet, J., Yang, X., Jones, P.A. & Liang, G. DNMT3B isoforms without catalytic activity stimulate gene body methylation as accessory proteins in somatic cells. *Nat Commun* **7**, 11453 (2016).
31. Masalmeh, R.H.A. *et al.* De novo DNA methyltransferase activity in colorectal cancer is directed towards H3K36me3 marked CpG islands. *Nat Commun* **12**, 694 (2021).
32. Wisniewski, J.R., Hein, M.Y., Cox, J. & Mann, M. A "proteomic ruler" for protein copy number and concentration estimation without spike-in standards. *Mol Cell Proteomics* **13**, 3497-506 (2014).
33. Gretarsson, K.H. *et al.* Cancer-associated DNA hypermethylation of Polycomb targets requires DNMT3A dual recognition of histone H2AK119 ubiquitination and the nucleosome acidic patch. *Sci Adv* **10**, eadp0975 (2024).
34. Medvedovic, J., Ebert, A., Tagoh, H. & Busslinger, M. Pax5: a master regulator of B cell development and leukemogenesis. *Adv Immunol* **111**, 179-206 (2011).
35. Nutt, S.L., Urbanek, P., Rolink, A. & Busslinger, M. Essential functions of Pax5 (BSAP) in pro-B cell development: difference between fetal and adult B lymphopoiesis and reduced V-to-DJ recombination at the IgH locus. *Genes Dev* **11**, 476-91 (1997).
36. Fuxa, M. *et al.* Pax5 induces V-to-DJ rearrangements and locus contraction of the immunoglobulin heavy-chain gene. *Genes Dev* **18**, 411-22 (2004).
37. Hill, L. *et al.* Wapl repression by Pax5 promotes V gene recombination by Igh loop extrusion. *Nature* **584**, 142-147 (2020).
38. Buske, C. *et al.* Deregulated expression of HOXB4 enhances the primitive growth activity of human hematopoietic cells. *Blood* **100**, 862-8 (2002).
39. Calvanese, V. *et al.* MLLT3 governs human haematopoietic stem-cell self-renewal and engraftment. *Nature* **576**, 281-286 (2019).
40. Christodoulou, C. *et al.* Live-animal imaging of native haematopoietic stem and progenitor cells. *Nature* **578**, 278-283 (2020).
41. Cobaleda, C., Schebesta, A., Delogu, A. & Busslinger, M. Pax5: the guardian of B cell identity and function. *Nat Immunol* **8**, 463-70 (2007).
42. Feinberg, M.W. *et al.* The Kruppel-like factor KLF4 is a critical regulator of monocyte differentiation. *EMBO J* **26**, 4138-48 (2007).
43. Frelin, C. *et al.* GATA-3 regulates the self-renewal of long-term hematopoietic stem cells. *Nat Immunol* **14**, 1037-44 (2013).
44. Hock, H. *et al.* Intrinsic requirement for zinc finger transcription factor Gfi-1 in neutrophil differentiation. *Immunity* **18**, 109-20 (2003).
45. Laslo, P. *et al.* Multilineage transcriptional priming and determination of alternate hematopoietic cell fates. *Cell* **126**, 755-66 (2006).

46. Lawrence, H.J. *et al.* Loss of expression of the Hoxa-9 homeobox gene impairs the proliferation and repopulating ability of hematopoietic stem cells. *Blood* **106**, 3988-94 (2005).
47. Lawrence, S.M., Corriden, R. & Nizet, V. The Ontogeny of a Neutrophil: Mechanisms of Granulopoiesis and Homeostasis. *Microbiol Mol Biol Rev* **82**(2018).
48. Lehnertz, B. *et al.* HLF expression defines the human hematopoietic stem cell state. *Blood* **138**, 2642-2654 (2021).
49. Lin, Y.C. *et al.* A global network of transcription factors, involving E2A, EBF1 and Foxo1, that orchestrates B cell fate. *Nat Immunol* **11**, 635-43 (2010).
50. Sauvageau, G. *et al.* Overexpression of HOXB4 in hematopoietic cells causes the selective expansion of more primitive populations in vitro and in vivo. *Genes Dev* **9**, 1753-65 (1995).
51. Schonheit, J. *et al.* PU.1 level-directed chromatin structure remodeling at the Irf8 gene drives dendritic cell commitment. *Cell Rep* **3**, 1617-28 (2013).
52. Weber, B.N. *et al.* A critical role for TCF-1 in T-lineage specification and differentiation. *Nature* **476**, 63-8 (2011).
53. Xiang, P. *et al.* A knock-in mouse strain facilitates dynamic tracking and enrichment of MEIS1. *Blood Adv* **1**, 2225-2235 (2017).
54. Yui, M.A. & Rothenberg, E.V. Developmental gene networks: a triathlon on the course to T cell identity. *Nat Rev Immunol* **14**, 529-45 (2014).

NG-A68576R1 Sarni *et al* – Responses to Reviewer 3 comments

Reviewer 3:

The authors have addressed most of my comments and added very good additional experiments to this new version.

I find it very exciting that HESJAS shows a novel form of monogenic progeria driven by gain of function of DNMT3A. Therefore, I fully support the author's conclusions that this mutation in DNMT3A causes accelerated hypermethylation at Polycomb promoters and leads to premature ageing, which makes these findings relevant for broad audience.

We thank the reviewer for their appreciation of the revised manuscript and its overall conclusions.

That said, I still do not agree with the claims of causality laid out in the current version. The authors indicate that the measured hypermethylation of Pax5 in Dnmt3aW326R/+ pre-pro-B cells is the reason for the failed differentiation to pro-B cells (new Fig 5). While indeed Dnmt3aW326R/+ inhibits this differentiation, and indeed there is hypermethylation observed in these cells, there is no experiment to support that DNA methylation at the Pax5 promoter directly influences this outcome (or transcription of Pax5).

While it appears to us the most likely explanation for reduced *Pax5* transcription and lymphopenia, we accept the reviewer's opinion that we have not provided an experiment that definitively demonstrates *Pax5* promoter methylation directly alters *Pax5* transcription.

Responding to the reviewer's point, we have therefore revised the discussion to state:

"hypermethylation of Pax5, and reduced activation of this gene during B cell development, implicates it in Dnmt3a$^{W326R/+}$ B cell lymphopenia. Dnmt3a may also have non-catalytic functions[85], meaning that effects of HESJAS mutations might be independent from observed DNA methylation gains, so future experiments will be necessary to confirm that Pax5 methylation directly accounts for reduced lymphocyte number. Nevertheless, the strong selection against Pax5 hypermethylation in late stage differentiation and mature B cells (Fig. 5g,h) argues in favour of a direct effect of DNA hypermethylation."

To address Reviewer 3's concerns about causal statements in the manuscript, we have also made the following textual changes, avoiding using the term 'causal' in our manuscript. Specific textual amendments are outlined below.

While the results in the current form suggest correlations, the authors write: L103: "we causally implicate DNA methylation-mediated stem cell exhaustion in the aetiology"

Now edited to: *"we implicate DNA methylation-mediated stem cell dysfunction in the aetiology of medically important age-related haematological, bone and metabolic pathologies, that might be targetable by future therapies."*

L150: "methylation at the promoter of a key transcription factor, Pax5, is the likely cause of reduced cellular output in this lineage to account for lymphopenia one of the age-related pathologies observed. Together, these findings provide experimental evidence supporting a causal link between hypermethylation and physiological ageing."

Edited to: *"we show that DNA methylation at the promoter of Pax5, encoding a key transcription factor, is associated with reduced cellular output in this lineage to account for lymphopenia, one of the age-related pathologies observed. Together, these findings provide experimental evidence linking hypermethylation to physiological ageing."*

L395: "DNA hypermethylation of a key lineage transcription factor in adult stem cells leads to impaired differentiation, promoting an age-related phenotype"

Edited to: *"Taken together, we conclude that DNA hypermethylation of a key lineage transcription factor in adult stem cells is associated with impaired differentiation, promoting an age-related phenotype."*

L485: "demonstrate that DNA hypermethylation slows transcriptional activation dynamics during differentiation" … "implicates such hypermethylation as the cause, at least of this pathology."

Edited to: *"The dogma is that DNA promoter methylation is repressive, so DNA hypermethylation substituting for Polycomb may act as an alternative repressive mark. Our transcriptomic analyses indicate that DNA hypermethylation could then impair transcriptional activation dynamics during*

differentiation. Specifically, hypermethylation of Pax5, and reduced activation of this gene during B cell development, implicates it in Dnmt3a$^{W326R/+}$ B cell lymphopenia."

If the authors want to show a causal relationship, they need to show that indeed the presence of methylation is leading to transcriptional repression of Pax5 and subsequently differentiation block with functional/mechanistic assays. And/or identify and test a mechanism that could link methylation to repression, i.e. blocked transcription factor binding, recruitment of MBD proteins. I agree that such would be too much asked for this manuscript, which is already extensive, but without such mechanistic insights, the authors should rephrase and exchange "causality" with "association".

We thank the reviewer for these experimental suggestions which hope we can pursue in future work.
